# Supplementary material for: Synaptic transmission parallels neuromodulation in a central food-intake circuit
Source: eLife. 2016 Nov 15;5:e16799. doi: 10.7554/eLife.16799 (PMC5182061; doi:10.7554/eLife.16799)
Supplement: Supplementary file 1. — Reconstructions of (A) hugin-PC, (B) hugin-VNC, (C) hugin-RG, (D) hugin-PH neurons, (E) insulin-producing cells (IPCs), (F) DH44-producing cells, (G) DMS-producing cells, (H) antennal nerve (AN) sensory neurons as clustered in Figure 6, (I) abdominal nerve sensory neurons, (J) paired interneurons and (K) unpaired medial interneurons. A dorsal view of each cell is shown on the left, and a frontal view on the right. Neuron ids (e.g. #123456) are provided to allow comparison between PDF and Blender atlas. Outline of the nervous system and the ring gland are shown in grey and dark grey, respectively. Table shows number of synapses of given neurons onto (left) and from (right) the hugin neuron represented in that row. Neurons are displayed as corresponding pairs of the left/right hemisegment with the exception of sensory neurons and unpaired medial interneurons. DOI: http://dx.doi.org/10.7554/eLife.16799.021 [file elife-16799-supp1.pdf]

A hugin-PC

1.

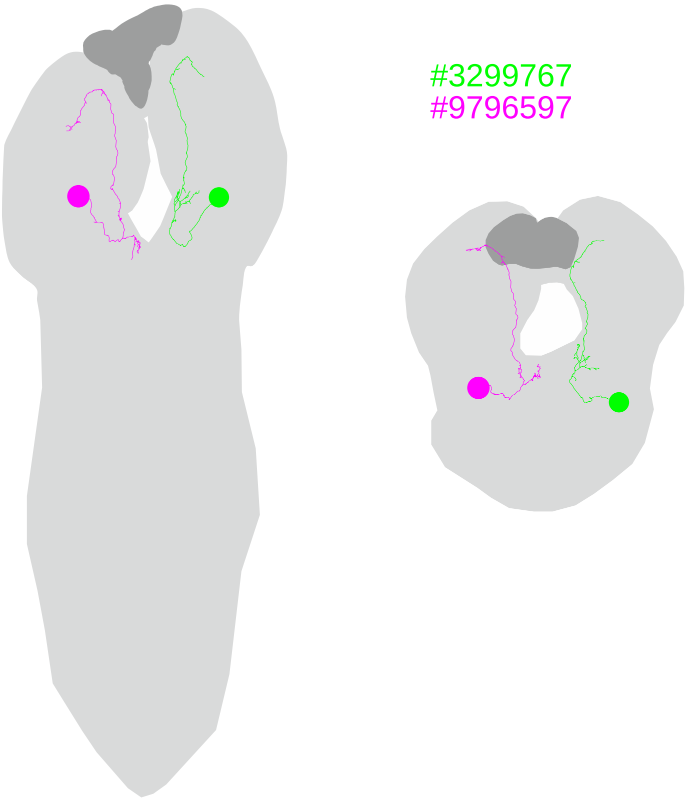

	synapses to		synapses from	
hugin-PC right 1	8	0	4	0
hugin-PC right 2	0	0	0	0
hugin-PC right 3	2	0	5	0
hugin-PC right 4	1	0	3	0
hugin-PC left 1	0	3	0	4
hugin-PC left 2	0	0	0	0
hugin-PC left 3	0	2	0	4
hugin-PC left 4	0	2	0	3
hugin-RG right 1	0	0	0	0
hugin-RG right 2	0	0	0	0
hugin-RG left 1	0	0	0	0
hugin-RG left 2	0	0	0	0
hugin-VNC right 1	0	1	0	0
hugin-VNC right 2	0	0	0	0
hugin-VNC left 1	0	0	0	0
hugin-VNC left 2	0	0	0	0
hugin-PH right 1	0	0	0	0
hugin-PH right 2	0	0	0	0
hugin-PH left 1	0	0	0	0
hugin-PH left 2	0	0	0	0

2.

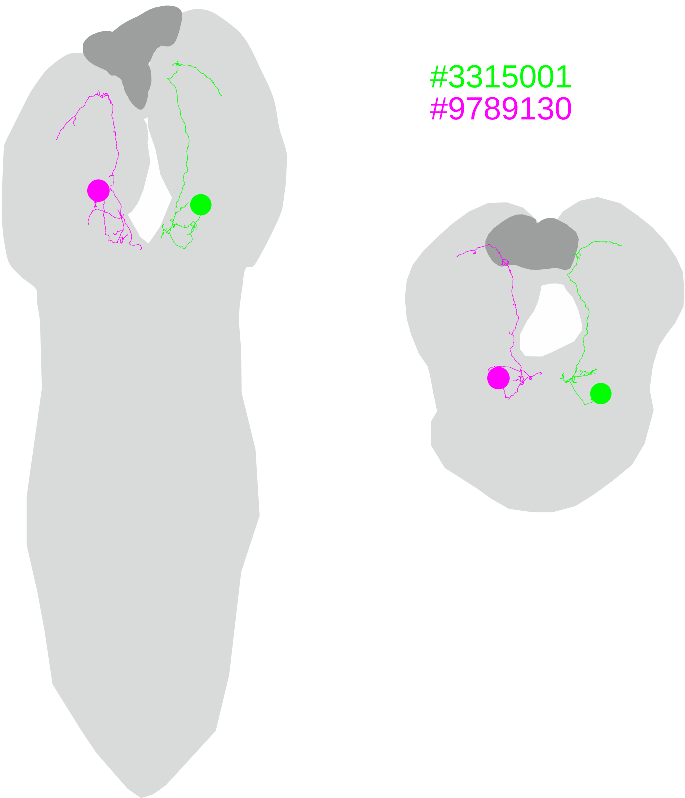

	synapses to		synapses from	
hugin-PC right 1	0	0	0	0
hugin-PC right 2	4	0	8	0
hugin-PC right 3	0	0	3	0
hugin-PC right 4	3	0	5	0
hugin-PC left 1	0	0	0	0
hugin-PC left 2	0	4	0	3
hugin-PC left 3	0	5	0	3
hugin-PC left 4	0	5	0	7
hugin-RG right 1	0	0	0	0
hugin-RG right 2	0	0	0	0
hugin-RG left 1	0	0	0	0
hugin-RG left 2	0	1	0	0
hugin-VNC right 1	0	0	0	0
hugin-VNC right 2	0	2	0	0
hugin-VNC left 1	0	0	0	0
hugin-VNC left 2	0	0	0	0
hugin-PH right 1	0	0	0	0
hugin-PH right 2	0	0	0	0
hugin-PH left 1	0	0	0	0
hugin-PH left 2	0	0	0	0

3.

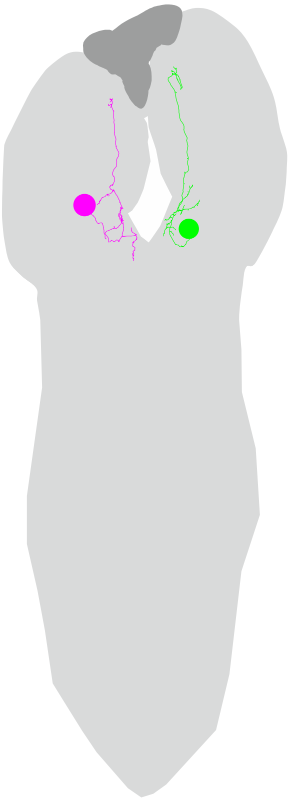

#9748579
#9805520

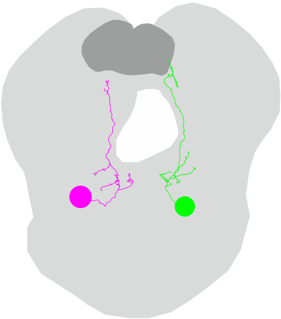

	synapses to		synapses from	
hugin-PC right 1	3	0	0	0
hugin-PC right 2	5	0	2	0
hugin-PC right 3	0	0	0	0
hugin-PC right 4	2	0	2	0
hugin-PC left 1	0	3	0	5
hugin-PC left 2	0	4	0	2
hugin-PC left 3	0	0	0	0
hugin-PC left 4	0	1	0	1
hugin-RG right 1	0	0	0	0
hugin-RG right 2	0	0	0	0
hugin-RG left 1	0	0	0	0
hugin-RG left 2	0	0	0	0
hugin-VNC right 1	0	0	0	0
hugin-VNC right 2	0	0	0	0
hugin-VNC left 1	0	0	0	0
hugin-VNC left 2	0	0	0	0
hugin-PH right 1	0	0	0	0
hugin-PH right 2	0	0	0	0
hugin-PH left 1	0	0	0	0
hugin-PH left 2	0	0	0	0

4.

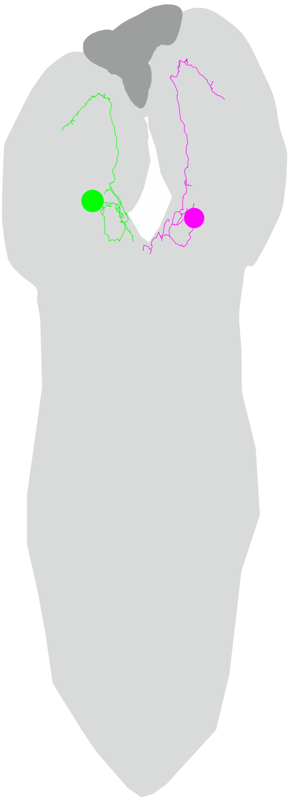

#9813902
#13581830

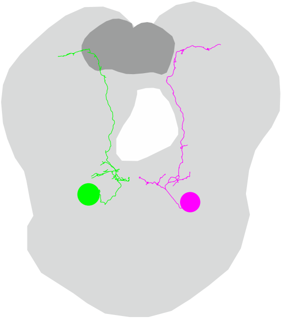

	synapses to		synapses from	
hugin-PC right 1	0	5	0	3
hugin-PC right 2	0	3	0	1
hugin-PC right 3	0	2	0	2
hugin-PC right 4	0	0	0	0
hugin-PC left 1	7	0	5	0
hugin-PC left 2	3	0	2	0
hugin-PC left 3	1	0	1	0
hugin-PC left 4	0	0	0	0
hugin-RG right 1	0	0	0	0
hugin-RG right 2	0	0	0	0
hugin-RG left 1	0	0	0	0
hugin-RG left 2	1	0	0	0
hugin-VNC right 1	0	0	0	0
hugin-VNC right 2	0	0	0	0
hugin-VNC left 1	0	0	0	0
hugin-VNC left 2	0	0	0	0
hugin-PH right 1	0	0	0	0
hugin-PH right 2	0	0	0	0
hugin-PH left 1	0	0	0	0
hugin-PH left 2	0	0	0	0

B hugin-VNC

1.

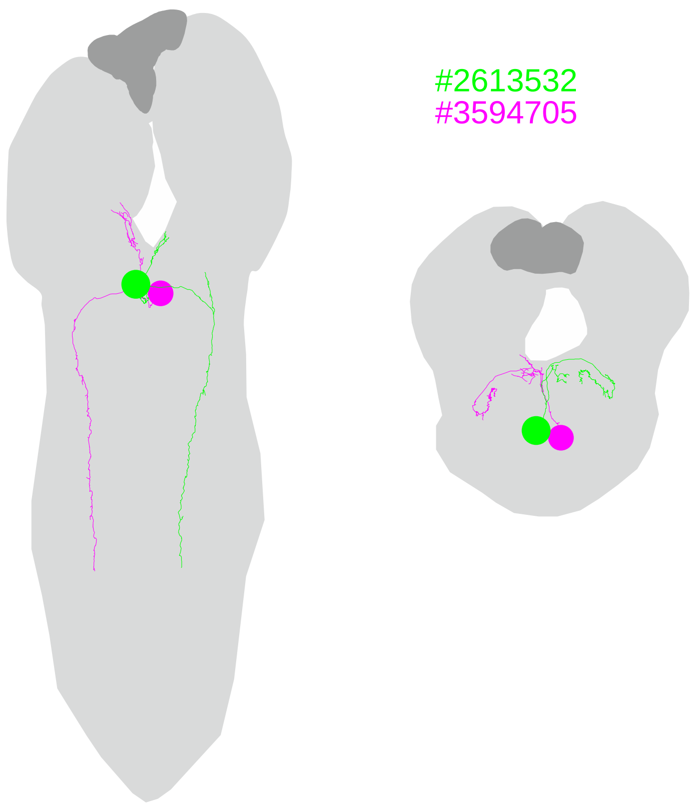

	synapses to		synapses from	
hugin-PC right 1	0	0	0	0
hugin-PC right 2	0	0	0	0
hugin-PC right 3	0	0	0	0
hugin-PC right 4	0	0	0	0
hugin-PC left 1	0	0	0	2
hugin-PC left 2	0	0	0	0
hugin-PC left 3	0	0	0	0
hugin-PC left 4	0	0	0	0
hugin-RG right 1	0	0	0	0
hugin-RG right 2	0	0	0	0
hugin-RG left 1	0	0	0	0
hugin-RG left 2	0	0	0	0
hugin-VNC right 1	0	2	0	5
hugin-VNC right 2	0	0	0	0
hugin-VNC left 1	7	0	6	0
hugin-VNC left 2	0	0	0	0
hugin-PH right 1	0	0	0	0
hugin-PH right 2	0	0	0	0
hugin-PH left 1	0	0	0	0
hugin-PH left 2	0	0	0	0

2.

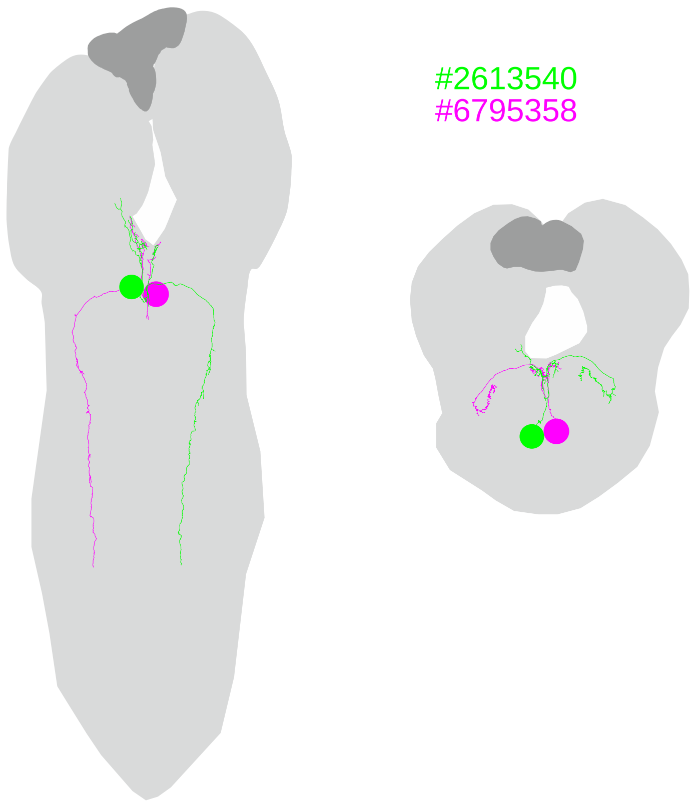

	synapses to		synapses from	
hugin-PC right 1	0	0	0	0
hugin-PC right 2	0	0	0	0
hugin-PC right 3	0	0	0	0
hugin-PC right 4	0	0	0	0
hugin-PC left 1	0	0	0	0
hugin-PC left 2	0	0	0	1
hugin-PC left 3	0	0	0	0
hugin-PC left 4	0	0	0	0
hugin-RG right 1	0	0	0	0
hugin-RG right 2	0	0	0	0
hugin-RG left 1	0	0	0	0
hugin-RG left 2	0	0	0	0
hugin-VNC right 1	0	0	0	0
hugin-VNC right 2	0	5	0	2
hugin-VNC left 1	0	0	0	0
hugin-VNC left 2	6	0	7	0
hugin-PH right 1	0	0	0	0
hugin-PH right 2	0	0	0	0
hugin-PH left 1	0	0	0	0
hugin-PH left 2	0	0	0	0

C hugin-RG

1.

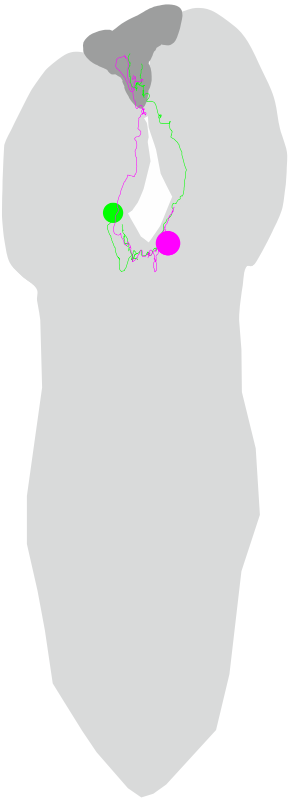

#2138427
#5038703

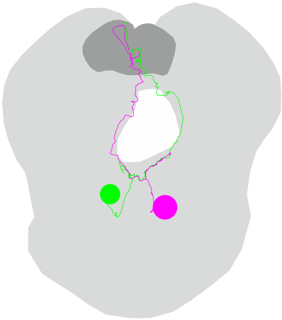

	synapses to		synapses from	
hugin-PC right 1	0	0	0	0
hugin-PC right 2	0	0	0	0
hugin-PC right 3	0	0	0	0
hugin-PC right 4	0	0	0	0
hugin-PC left 1	0	0	0	0
hugin-PC left 2	0	0	0	0
hugin-PC left 3	0	0	0	0
hugin-PC left 4	0	0	0	0
hugin-RG right 1	0	0	0	0
hugin-RG right 2	0	0	0	0
hugin-RG left 1	0	0	0	0
hugin-RG left 2	0	0	0	0
hugin-VNC right 1	0	0	0	0
hugin-VNC right 2	0	0	0	0
hugin-VNC left 1	0	0	0	0
hugin-VNC left 2	0	0	0	0
hugin-PH right 1	0	0	0	0
hugin-PH right 2	0	0	0	0
hugin-PH left 1	0	0	0	0
hugin-PH left 2	0	0	0	0

2.

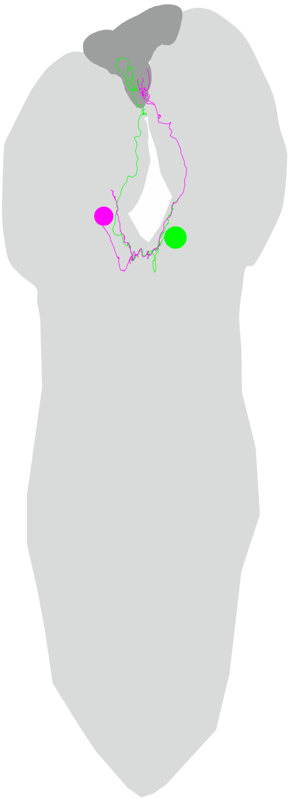

#5601924
#5615961

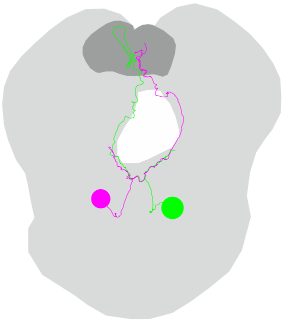

	synapses to		synapses from	
hugin-PC right 1	0	0	0	0
hugin-PC right 2	0	0	0	0
hugin-PC right 3	0	0	0	0
hugin-PC right 4	0	0	0	0
hugin-PC left 1	0	0	0	1
hugin-PC left 2	0	0	0	0
hugin-PC left 3	0	0	0	0
hugin-PC left 4	0	0	0	1
hugin-RG right 1	0	0	0	0
hugin-RG right 2	0	0	0	0
hugin-RG left 1	0	0	0	0
hugin-RG left 2	0	0	0	0
hugin-VNC right 1	0	0	0	0
hugin-VNC right 2	0	0	0	0
hugin-VNC left 1	0	0	0	0
hugin-VNC left 2	0	0	0	0
hugin-PH right 1	0	0	0	0
hugin-PH right 2	0	0	0	0
hugin-PH left 1	0	0	0	0
hugin-PH left 2	0	0	0	0

D hugin-PH

1.

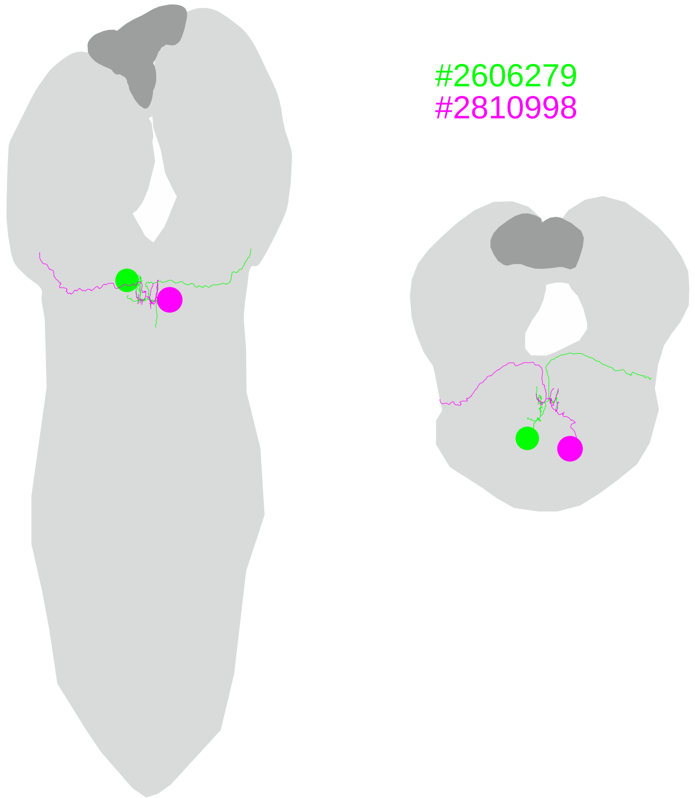

	synapses to		synapses from	
hugin-PC right 1	0	0	0	0
hugin-PC right 2	0	0	0	0
hugin-PC right 3	0	0	0	0
hugin-PC right 4	0	0	0	0
hugin-PC left 1	0	0	0	0
hugin-PC left 2	0	0	0	0
hugin-PC left 3	0	0	0	0
hugin-PC left 4	0	0	0	0
hugin-RG right 1	0	0	0	0
hugin-RG right 2	0	0	0	0
hugin-RG left 1	0	0	0	0
hugin-RG left 2	0	0	0	0
hugin-VNC right 1	0	0	0	0
hugin-VNC right 2	0	0	0	0
hugin-VNC left 1	0	0	0	0
hugin-VNC left 2	0	0	0	0
hugin-PH right 1	0	0	0	0
hugin-PH right 2	0	0	0	0
hugin-PH left 1	0	0	0	1
hugin-PH left 2	0	0	0	0

2.

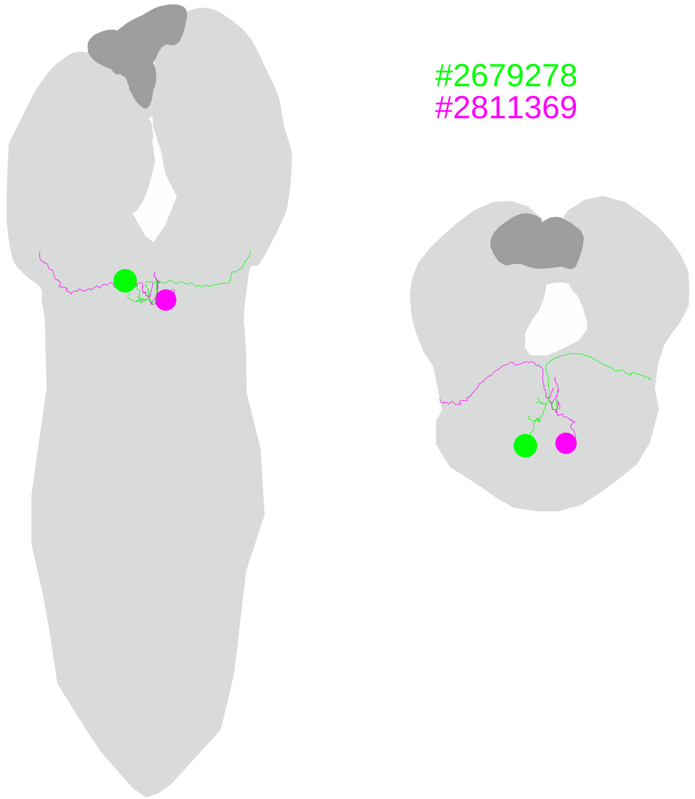

	synapses to		synapses from	
hugin-PC right 1	0	0	0	0
hugin-PC right 2	0	0	0	0
hugin-PC right 3	0	0	0	0
hugin-PC right 4	0	0	0	0
hugin-PC left 1	0	0	0	0
hugin-PC left 2	0	0	0	0
hugin-PC left 3	0	0	0	0
hugin-PC left 4	0	0	0	0
hugin-RG right 1	0	0	0	0
hugin-RG right 2	0	0	0	0
hugin-RG left 1	0	0	0	0
hugin-RG left 2	0	0	0	0
hugin-VNC right 1	0	0	0	0
hugin-VNC right 2	0	0	0	0
hugin-VNC left 1	0	0	0	0
hugin-VNC left 2	0	0	0	0
hugin-PH right 1	0	0	0	0
hugin-PH right 2	1	0	0	0
hugin-PH left 1	0	0	0	0
hugin-PH left 2	0	0	0	0

E Insulin-producing cells (IPCs)

1.

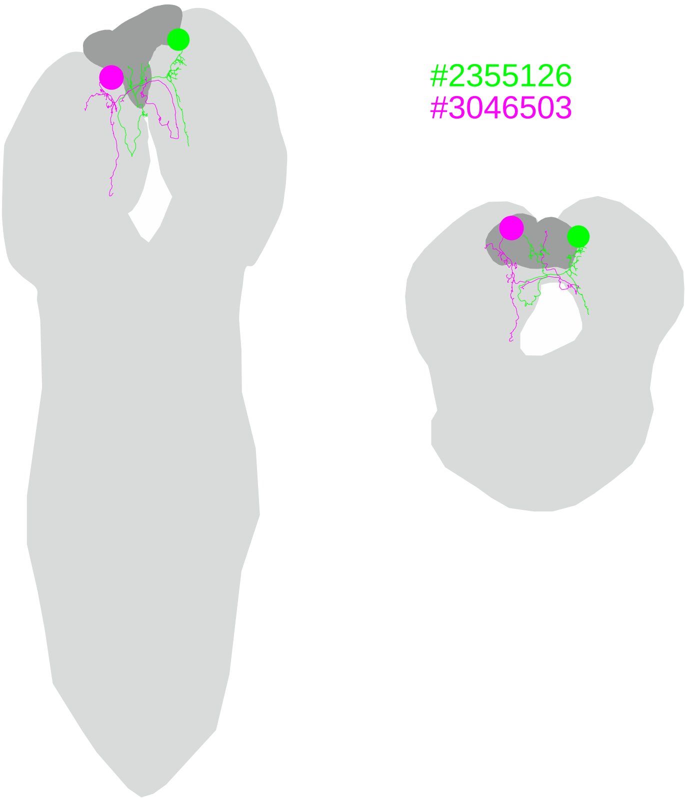

	synapses to		synapses from	
hugin-PC right 1	0	0	2	0
hugin-PC right 2	0	0	6	0
hugin-PC right 3	0	0	2	0
hugin-PC right 4	0	0	0	0
hugin-PC left 1	0	0	0	2
hugin-PC left 2	0	0	0	0
hugin-PC left 3	0	0	0	2
hugin-PC left 4	0	0	0	2
hugin-RG right 1	0	0	0	0
hugin-RG right 2	0	0	0	0
hugin-RG left 1	0	0	0	0
hugin-RG left 2	0	0	0	0
hugin-VNC right 1	0	0	0	0
hugin-VNC right 2	0	0	0	0
hugin-VNC left 1	0	0	0	0
hugin-VNC left 2	0	0	0	0
hugin-PH right 1	0	0	0	0
hugin-PH right 2	0	0	0	0
hugin-PH left 1	0	0	0	0
hugin-PH left 2	0	0	0	0

2.

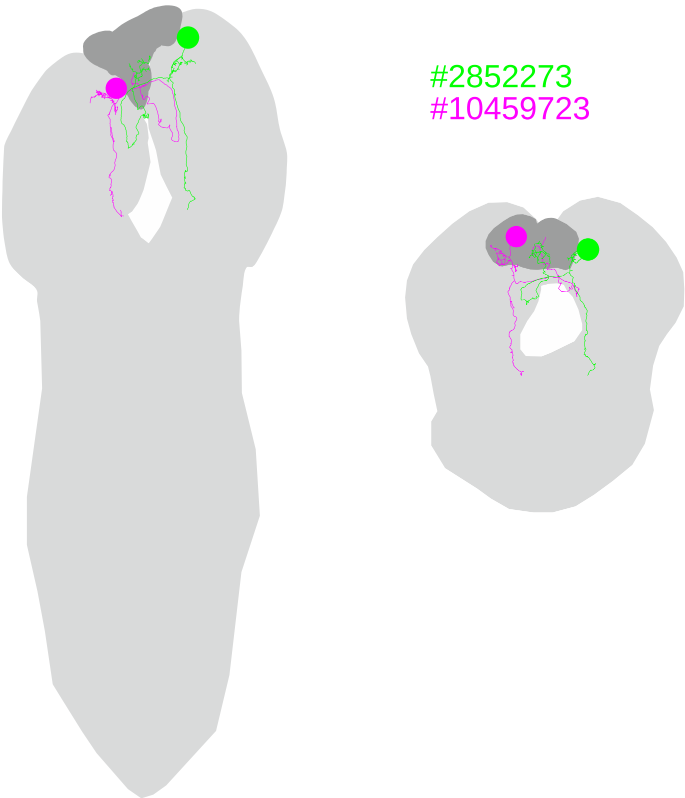

	synapses to		synapses from	
hugin-PC right 1	0	0	3	0
hugin-PC right 2	0	0	5	0
hugin-PC right 3	0	0	1	0
hugin-PC right 4	0	0	1	0
hugin-PC left 1	0	0	0	2
hugin-PC left 2	0	0	0	1
hugin-PC left 3	0	0	0	0
hugin-PC left 4	0	0	0	3
hugin-RG right 1	0	0	1	0
hugin-RG right 2	0	0	0	0
hugin-RG left 1	0	0	0	0
hugin-RG left 2	0	0	0	0
hugin-VNC right 1	0	0	0	0
hugin-VNC right 2	0	0	0	0
hugin-VNC left 1	0	0	0	0
hugin-VNC left 2	0	0	0	0
hugin-PH right 1	0	0	0	0
hugin-PH right 2	0	0	0	0
hugin-PH left 1	0	0	0	0
hugin-PH left 2	0	0	0	0

3.

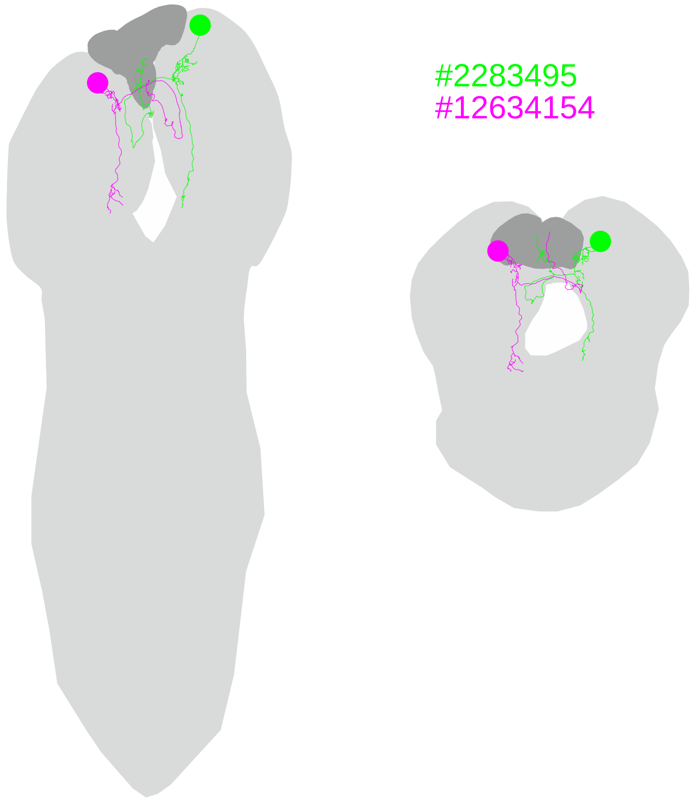

	synapses to		synapses from	
hugin-PC right 1	0	0	4	0
hugin-PC right 2	0	0	5	0
hugin-PC right 3	0	0	3	0
hugin-PC right 4	0	0	1	0
hugin-PC left 1	0	0	0	0
hugin-PC left 2	0	0	0	5
hugin-PC left 3	0	0	0	3
hugin-PC left 4	0	0	0	3
hugin-RG right 1	0	0	0	0
hugin-RG right 2	0	0	1	0
hugin-RG left 1	0	0	0	0
hugin-RG left 2	0	0	0	0
hugin-VNC right 1	0	0	0	0
hugin-VNC right 2	0	0	0	0
hugin-VNC left 1	0	0	0	0
hugin-VNC left 2	0	0	0	0
hugin-PH right 1	0	0	0	0
hugin-PH right 2	0	0	0	0
hugin-PH left 1	0	0	0	0
hugin-PH left 2	0	0	0	0

4.

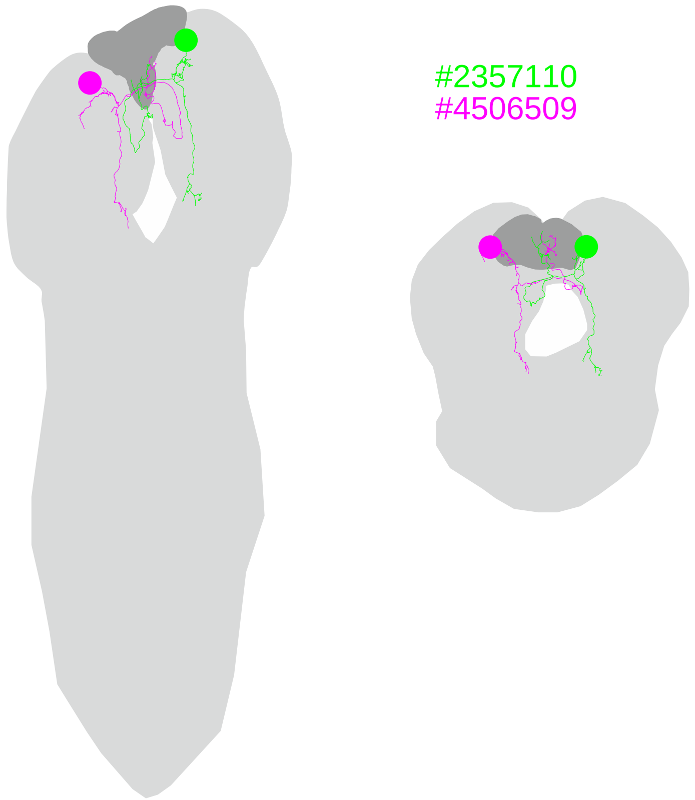

	synapses to		synapses from	
hugin-PC right 1	0	0	3	0
hugin-PC right 2	0	0	3	0
hugin-PC right 3	0	0	0	0
hugin-PC right 4	0	0	1	0
hugin-PC left 1	0	0	0	9
hugin-PC left 2	0	0	0	1
hugin-PC left 3	0	0	0	1
hugin-PC left 4	0	0	0	2
hugin-RG right 1	0	0	0	0
hugin-RG right 2	0	0	0	0
hugin-RG left 1	0	0	0	0
hugin-RG left 2	0	0	0	0
hugin-VNC right 1	0	0	0	0
hugin-VNC right 2	0	0	0	0
hugin-VNC left 1	0	0	0	0
hugin-VNC left 2	0	0	0	0
hugin-PH right 1	0	0	0	0
hugin-PH right 2	0	0	0	0
hugin-PH left 1	0	0	0	0
hugin-PH left 2	0	0	0	0

5.

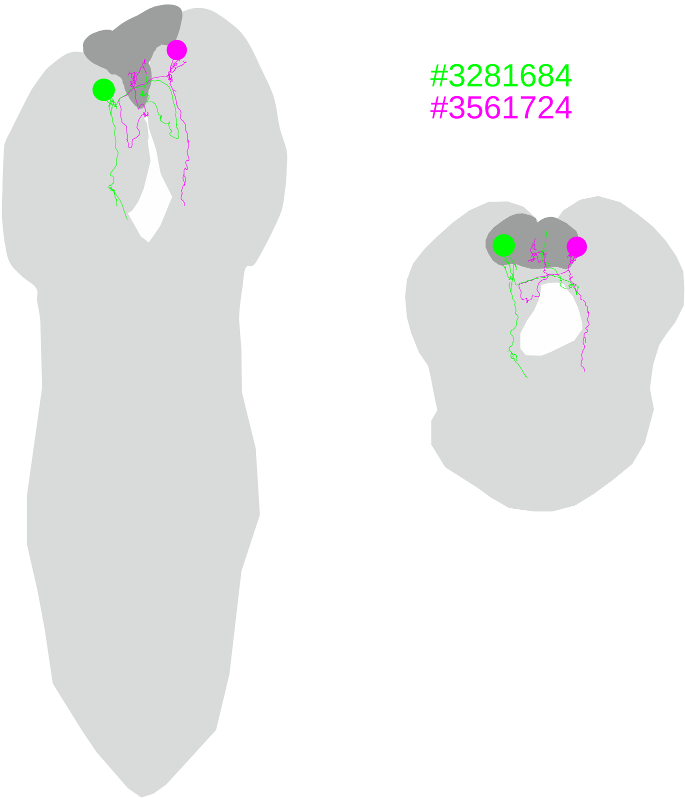

	synapses to		synapses from	
hugin-PC right 1	0	0	0	1
hugin-PC right 2	0	0	0	4
hugin-PC right 3	0	0	0	5
hugin-PC right 4	0	0	0	0
hugin-PC left 1	0	0	3	0
hugin-PC left 2	0	0	3	0
hugin-PC left 3	0	0	3	0
hugin-PC left 4	0	0	2	0
hugin-RG right 1	0	0	0	0
hugin-RG right 2	0	0	0	0
hugin-RG left 1	0	0	0	0
hugin-RG left 2	0	0	0	0
hugin-VNC right 1	0	0	0	0
hugin-VNC right 2	0	0	0	0
hugin-VNC left 1	0	0	0	0
hugin-VNC left 2	0	0	0	0
hugin-PH right 1	0	0	0	0
hugin-PH right 2	0	0	0	0
hugin-PH left 1	0	0	0	0
hugin-PH left 2	0	0	0	0

6.

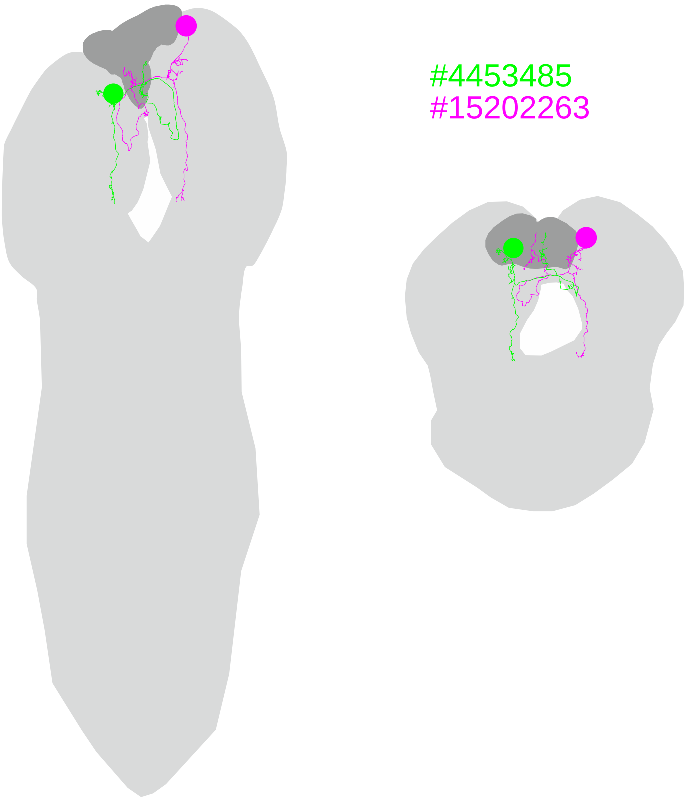

	synapses to		synapses from	
hugin-PC right 1	0	0	0	7
hugin-PC right 2	0	0	0	1
hugin-PC right 3	0	0	0	1
hugin-PC right 4	0	0	0	1
hugin-PC left 1	0	0	4	0
hugin-PC left 2	0	0	3	0
hugin-PC left 3	0	0	3	0
hugin-PC left 4	0	0	0	0
hugin-RG right 1	0	0	0	0
hugin-RG right 2	0	0	0	0
hugin-RG left 1	0	0	0	0
hugin-RG left 2	0	0	1	0
hugin-VNC right 1	0	0	0	0
hugin-VNC right 2	0	0	0	0
hugin-VNC left 1	0	0	0	0
hugin-VNC left 2	0	0	0	0
hugin-PH right 1	0	0	0	0
hugin-PH right 2	0	0	0	0
hugin-PH left 1	0	0	0	0
hugin-PH left 2	0	0	0	0

7.

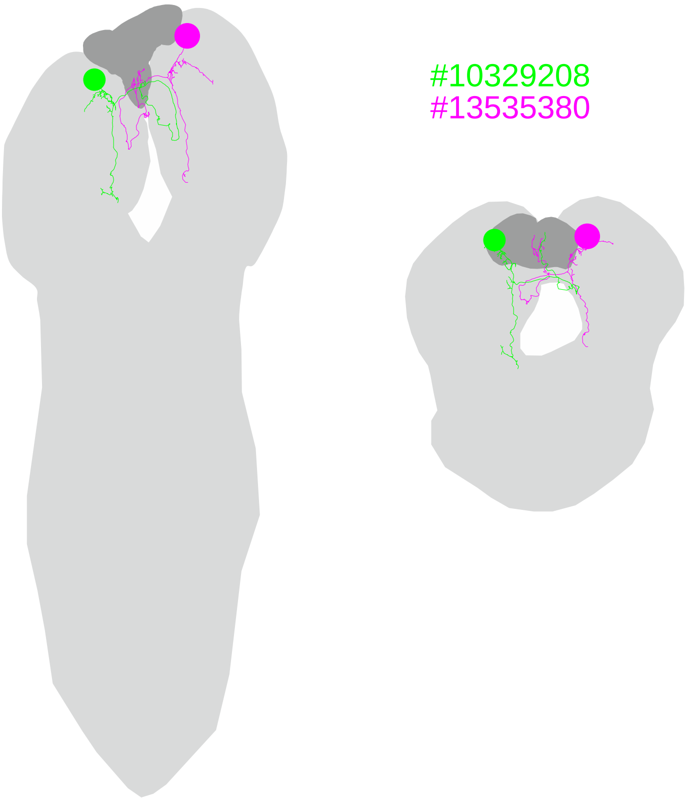

	synapses to		synapses from	
hugin-PC right 1	0	1	0	7
hugin-PC right 2	0	0	0	3
hugin-PC right 3	0	0	0	3
hugin-PC right 4	0	0	0	1
hugin-PC left 1	0	0	7	0
hugin-PC left 2	0	0	2	0
hugin-PC left 3	0	0	4	0
hugin-PC left 4	0	0	1	0
hugin-RG right 1	0	0	0	0
hugin-RG right 2	0	0	0	0
hugin-RG left 1	0	0	0	0
hugin-RG left 2	0	0	0	0
hugin-VNC right 1	0	0	0	0
hugin-VNC right 2	0	0	0	0
hugin-VNC left 1	0	0	0	0
hugin-VNC left 2	0	0	0	0
hugin-PH right 1	0	0	0	0
hugin-PH right 2	0	0	0	0
hugin-PH left 1	0	0	0	0
hugin-PH left 2	0	0	0	0

F DH44-producing mNSCs

1.

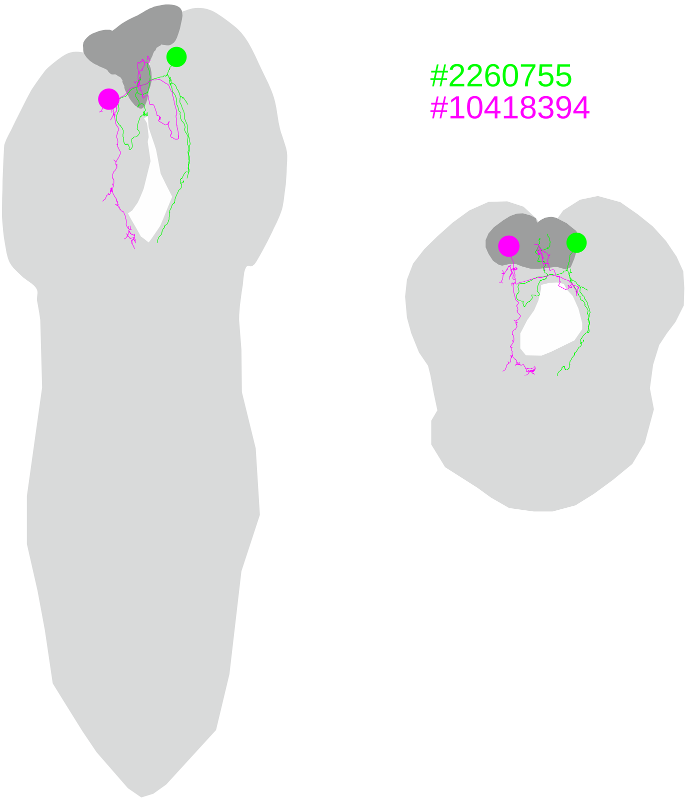

	synapses to		synapses from	
hugin-PC right 1	0	0	0	0
hugin-PC right 2	0	0	1	0
hugin-PC right 3	0	0	1	0
hugin-PC right 4	0	0	0	0
hugin-PC left 1	0	0	0	1
hugin-PC left 2	0	0	0	0
hugin-PC left 3	0	0	0	2
hugin-PC left 4	0	0	0	0
hugin-RG right 1	0	0	0	0
hugin-RG right 2	0	0	0	0
hugin-RG left 1	0	0	0	0
hugin-RG left 2	0	0	0	0
hugin-VNC right 1	0	0	0	0
hugin-VNC right 2	0	0	0	0
hugin-VNC left 1	0	0	0	0
hugin-VNC left 2	0	0	0	0
hugin-PH right 1	0	0	0	0
hugin-PH right 2	0	0	0	0
hugin-PH left 1	0	0	0	0
hugin-PH left 2	0	0	0	0

2.

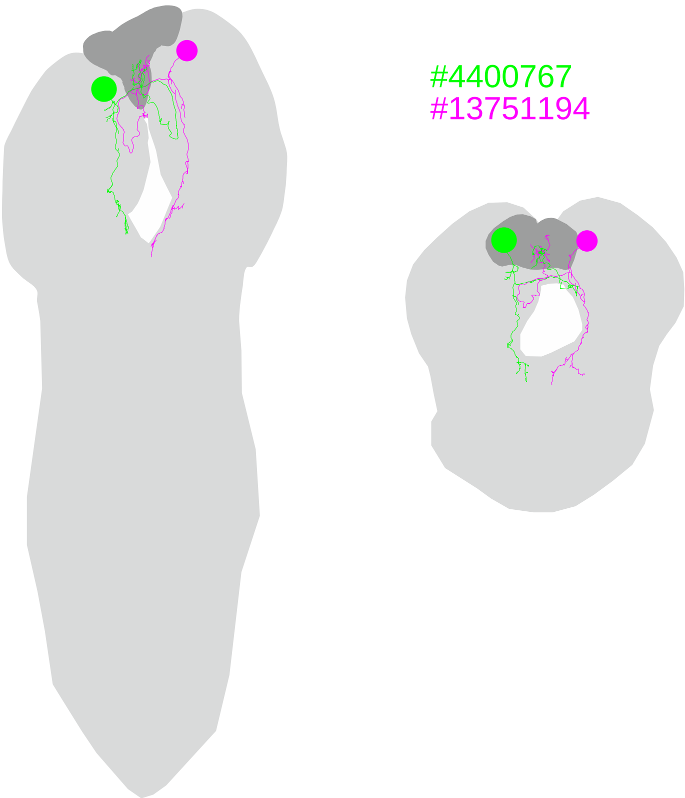

	synapses to		synapses from	
hugin-PC right 1	0	0	0	0
hugin-PC right 2	0	0	0	0
hugin-PC right 3	0	0	0	0
hugin-PC right 4	0	0	0	0
hugin-PC left 1	0	0	0	0
hugin-PC left 2	0	0	0	0
hugin-PC left 3	0	0	1	0
hugin-PC left 4	0	0	0	0
hugin-RG right 1	0	0	0	0
hugin-RG right 2	0	0	0	1
hugin-RG left 1	0	0	0	0
hugin-RG left 2	0	0	0	0
hugin-VNC right 1	0	0	0	0
hugin-VNC right 2	0	0	0	0
hugin-VNC left 1	0	0	0	0
hugin-VNC left 2	0	0	0	0
hugin-PH right 1	0	0	0	0
hugin-PH right 2	0	0	0	0
hugin-PH left 1	0	0	0	0
hugin-PH left 2	0	0	0	0

3.

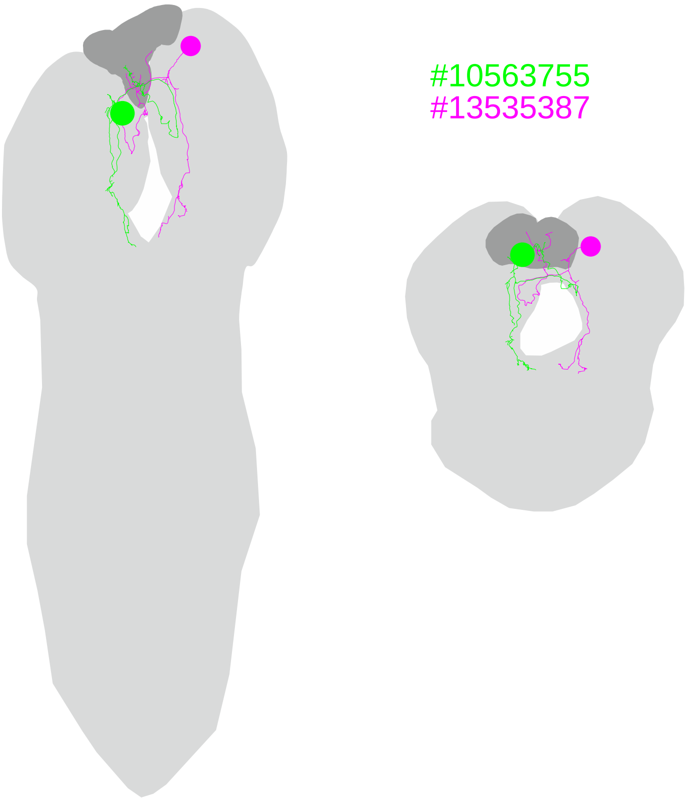

	synapses to		synapses from	
hugin-PC right 1	0	0	0	0
hugin-PC right 2	0	0	0	0
hugin-PC right 3	0	0	0	0
hugin-PC right 4	0	0	0	0
hugin-PC left 1	0	0	0	0
hugin-PC left 2	0	0	0	0
hugin-PC left 3	0	0	0	0
hugin-PC left 4	0	0	0	0
hugin-RG right 1	0	0	0	0
hugin-RG right 2	0	0	0	0
hugin-RG left 1	0	0	0	0
hugin-RG left 2	0	0	0	0
hugin-VNC right 1	0	0	0	0
hugin-VNC right 2	0	0	0	0
hugin-VNC left 1	0	0	0	0
hugin-VNC left 2	0	0	0	0
hugin-PH right 1	0	0	0	0
hugin-PH right 2	0	0	0	0
hugin-PH left 1	0	0	0	0
hugin-PH left 2	0	0	0	0

G DMS-producing mNSCs

1.

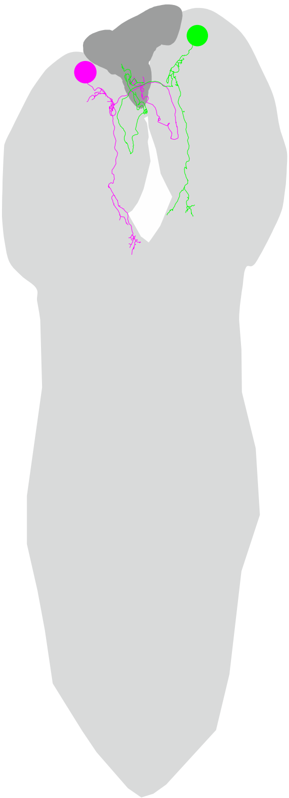

#2659386
#4523584

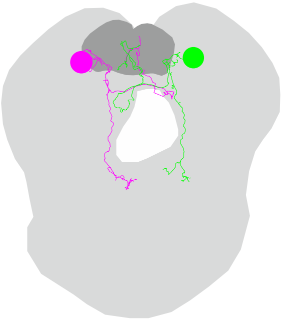

	synapses to		synapses from	
hugin-PC right 1	0	0	0	0
hugin-PC right 2	0	0	0	0
hugin-PC right 3	0	0	0	0
hugin-PC right 4	0	0	1	0
hugin-PC left 1	0	0	0	1
hugin-PC left 2	0	0	0	3
hugin-PC left 3	0	0	0	0
hugin-PC left 4	0	0	0	3
hugin-RG right 1	0	0	0	0
hugin-RG right 2	0	0	0	0
hugin-RG left 1	0	0	0	0
hugin-RG left 2	0	0	0	0
hugin-VNC right 1	0	0	0	0
hugin-VNC right 2	0	0	0	0
hugin-VNC left 1	0	0	0	0
hugin-VNC left 2	0	0	0	0
hugin-PH right 1	0	0	0	0
hugin-PH right 2	0	0	0	0
hugin-PH left 1	0	0	0	0
hugin-PH left 2	0	0	0	0

2.

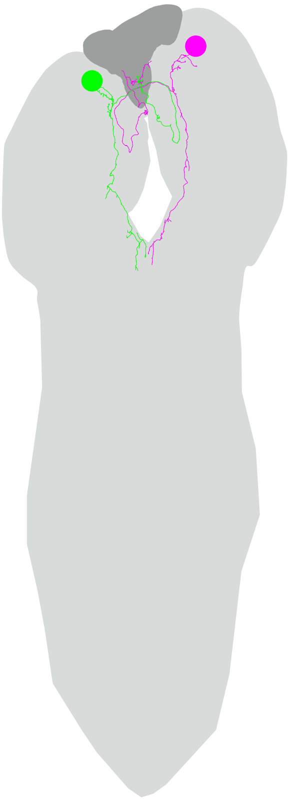

#4523803
#13793212

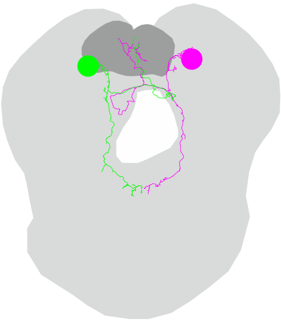

	synapses to		synapses from	
hugin-PC right 1	0	0	0	0
hugin-PC right 2	0	0	0	0
hugin-PC right 3	0	0	0	0
hugin-PC right 4	0	0	0	0
hugin-PC left 1	0	0	1	0
hugin-PC left 2	0	0	0	0
hugin-PC left 3	0	0	2	0
hugin-PC left 4	0	0	0	0
hugin-RG right 1	0	0	0	0
hugin-RG right 2	0	0	0	0
hugin-RG left 1	0	0	0	0
hugin-RG left 2	0	0	0	0
hugin-VNC right 1	0	0	0	0
hugin-VNC right 2	0	0	0	0
hugin-VNC left 1	0	0	0	0
hugin-VNC left 2	0	0	0	0
hugin-PH right 1	0	0	0	0
hugin-PH right 2	0	0	0	0
hugin-PH left 1	0	0	0	0
hugin-PH left 2	0	0	0	0

H AN sensory neurons - C1

1.

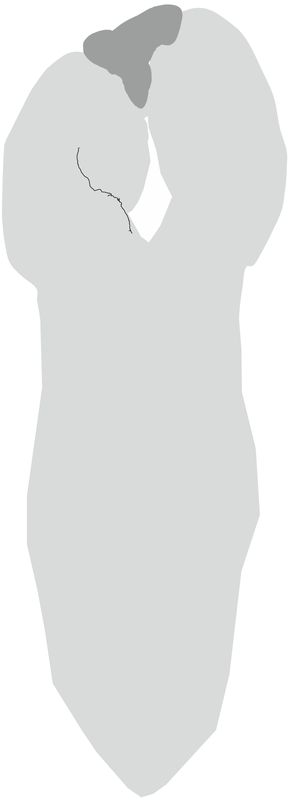

#1377586

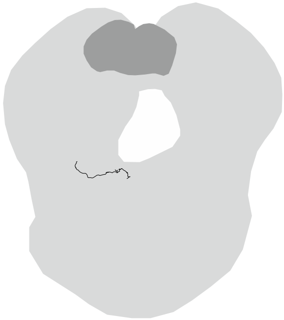

	synapses to	synapses from
hugin-PC right 1	0	0
hugin-PC right 2	0	0
hugin-PC right 3	0	0
hugin-PC right 4	0	0
hugin-PC left 1	0	0
hugin-PC left 2	0	0
hugin-PC left 3	0	0
hugin-PC left 4	0	0
hugin-RG right 1	0	0
hugin-RG right 2	0	0
hugin-RG left 1	0	0
hugin-RG left 2	0	0
hugin-VNC right 1	1	0
hugin-VNC right 2	2	0
hugin-VNC left 1	0	0
hugin-VNC left 2	0	0
hugin-PH right 1	0	0
hugin-PH right 2	0	0
hugin-PH left 1	0	0
hugin-PH left 2	0	0

2.

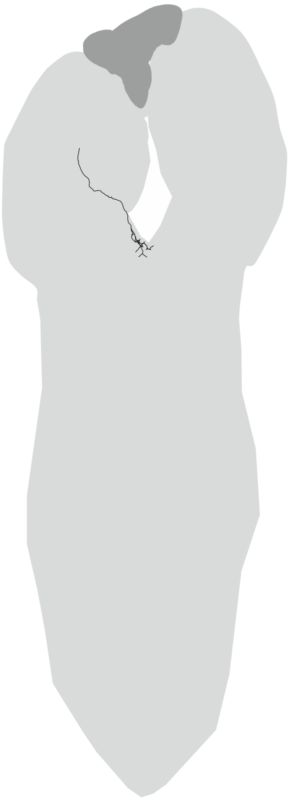

#15769419

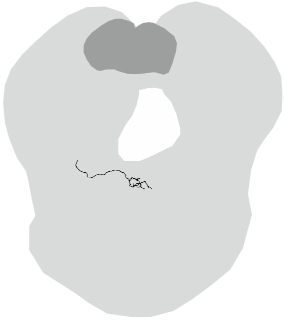

	synapses to	synapses from
hugin-PC right 1	0	0
hugin-PC right 2	0	0
hugin-PC right 3	0	0
hugin-PC right 4	0	0
hugin-PC left 1	0	0
hugin-PC left 2	0	0
hugin-PC left 3	1	0
hugin-PC left 4	0	0
hugin-RG right 1	0	0
hugin-RG right 2	0	0
hugin-RG left 1	0	0
hugin-RG left 2	0	0
hugin-VNC right 1	2	0
hugin-VNC right 2	0	0
hugin-VNC left 1	2	0
hugin-VNC left 2	0	0
hugin-PH right 1	0	0
hugin-PH right 2	0	0
hugin-PH left 1	0	0
hugin-PH left 2	0	0

3.

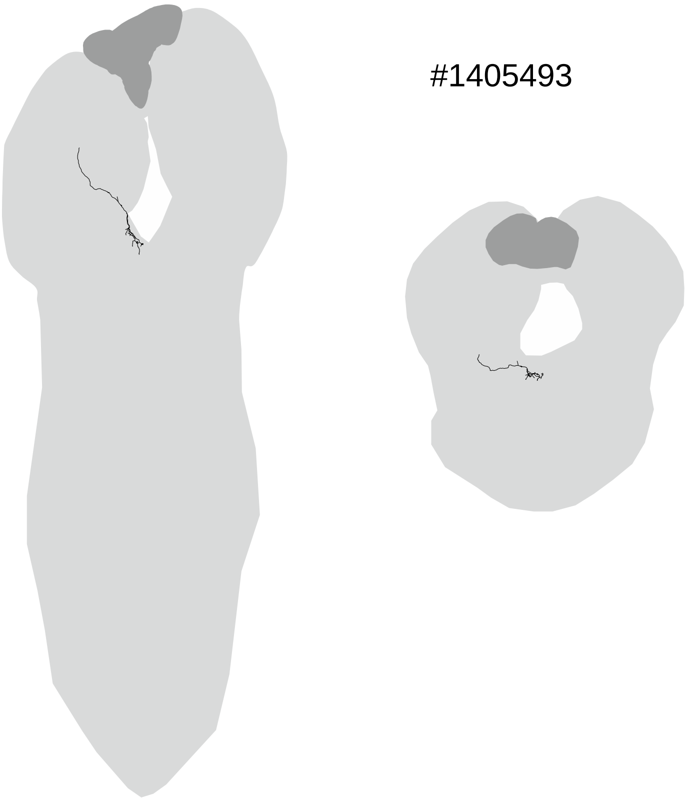

#1405493

	synapses to	synapses from
hugin-PC right 1	0	0
hugin-PC right 2	0	0
hugin-PC right 3	0	0
hugin-PC right 4	0	0
hugin-PC left 1	1	0
hugin-PC left 2	1	0
hugin-PC left 3	0	0
hugin-PC left 4	3	0
hugin-RG right 1	0	0
hugin-RG right 2	0	0
hugin-RG left 1	0	0
hugin-RG left 2	0	0
hugin-VNC right 1	3	0
hugin-VNC right 2	0	0
hugin-VNC left 1	1	0
hugin-VNC left 2	0	0
hugin-PH right 1	0	0
hugin-PH right 2	0	0
hugin-PH left 1	0	0
hugin-PH left 2	0	0

4.

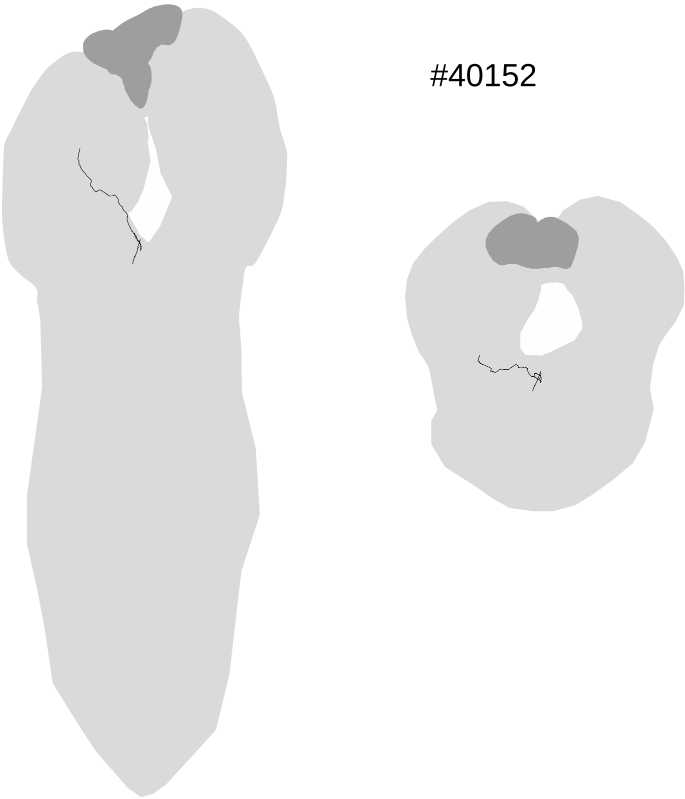

#40152

	synapses to	synapses from
hugin-PC right 1	0	0
hugin-PC right 2	0	0
hugin-PC right 3	0	0
hugin-PC right 4	0	0
hugin-PC left 1	2	0
hugin-PC left 2	2	0
hugin-PC left 3	2	0
hugin-PC left 4	0	0
hugin-RG right 1	0	0
hugin-RG right 2	0	0
hugin-RG left 1	0	0
hugin-RG left 2	0	0
hugin-VNC right 1	0	0
hugin-VNC right 2	1	0
hugin-VNC left 1	1	0
hugin-VNC left 2	0	0
hugin-PH right 1	0	0
hugin-PH right 2	0	0
hugin-PH left 1	0	0
hugin-PH left 2	0	0

5.

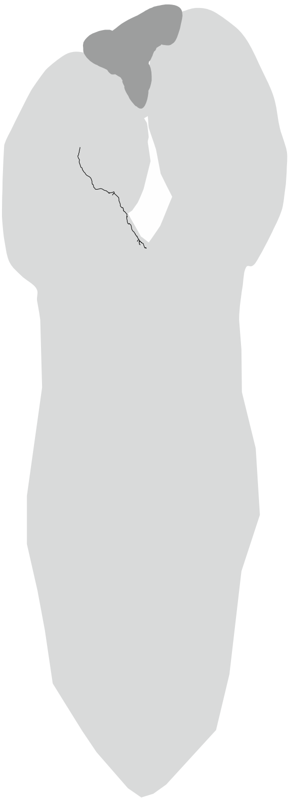

#1395971

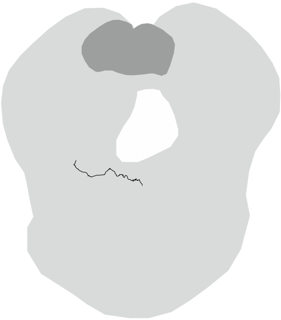

	synapses to	synapses from
hugin-PC right 1	0	0
hugin-PC right 2	0	0
hugin-PC right 3	0	0
hugin-PC right 4	0	0
hugin-PC left 1	0	0
hugin-PC left 2	1	0
hugin-PC left 3	2	0
hugin-PC left 4	0	0
hugin-RG right 1	0	0
hugin-RG right 2	0	0
hugin-RG left 1	0	0
hugin-RG left 2	0	0
hugin-VNC right 1	2	0
hugin-VNC right 2	6	0
hugin-VNC left 1	2	0
hugin-VNC left 2	0	0
hugin-PH right 1	0	0
hugin-PH right 2	0	0
hugin-PH left 1	0	0
hugin-PH left 2	0	0

6.

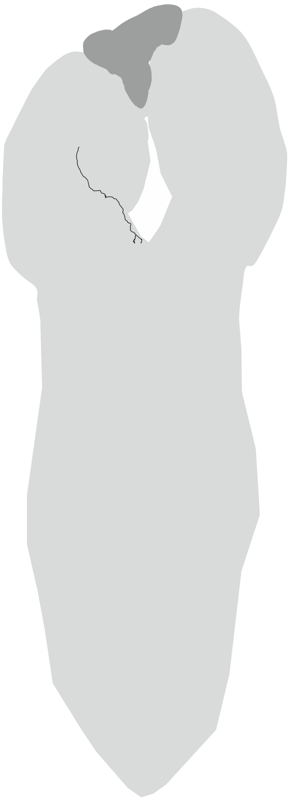

#9838294

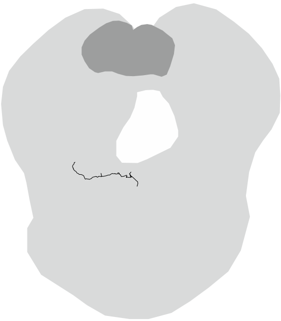

	synapses to	synapses from
hugin-PC right 1	0	0
hugin-PC right 2	0	0
hugin-PC right 3	0	0
hugin-PC right 4	0	0
hugin-PC left 1	0	0
hugin-PC left 2	0	0
hugin-PC left 3	0	0
hugin-PC left 4	0	1
hugin-RG right 1	0	0
hugin-RG right 2	0	0
hugin-RG left 1	0	0
hugin-RG left 2	0	0
hugin-VNC right 1	2	0
hugin-VNC right 2	0	0
hugin-VNC left 1	1	0
hugin-VNC left 2	0	0
hugin-PH right 1	0	0
hugin-PH right 2	0	0
hugin-PH left 1	0	0
hugin-PH left 2	0	0

7.

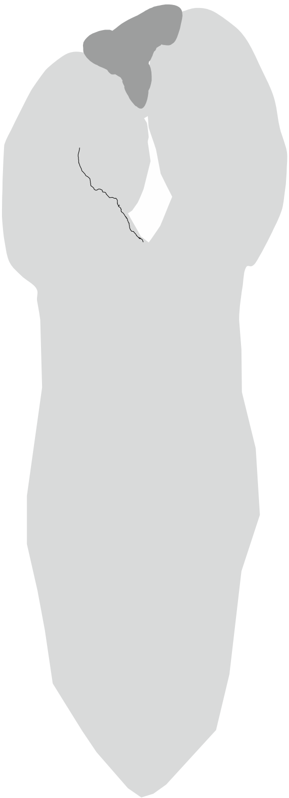

#1406463

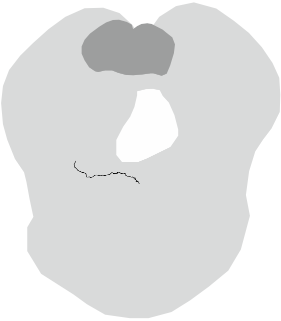

	synapses to	synapses from
hugin-PC right 1	0	0
hugin-PC right 2	0	0
hugin-PC right 3	0	0
hugin-PC right 4	0	0
hugin-PC left 1	0	0
hugin-PC left 2	0	0
hugin-PC left 3	0	0
hugin-PC left 4	1	0
hugin-RG right 1	0	0
hugin-RG right 2	0	0
hugin-RG left 1	0	0
hugin-RG left 2	0	0
hugin-VNC right 1	1	0
hugin-VNC right 2	0	0
hugin-VNC left 1	0	0
hugin-VNC left 2	0	0
hugin-PH right 1	0	0
hugin-PH right 2	0	0
hugin-PH left 1	0	0
hugin-PH left 2	0	0

8.

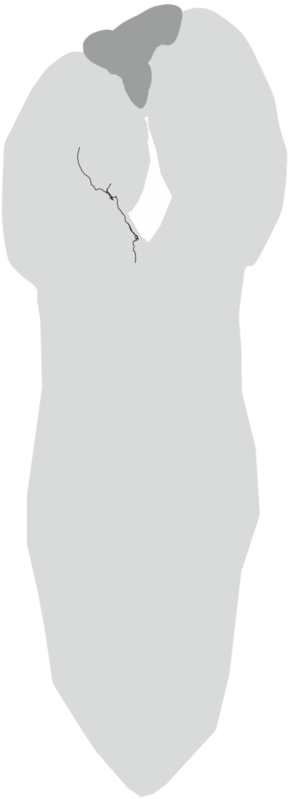

#1398253

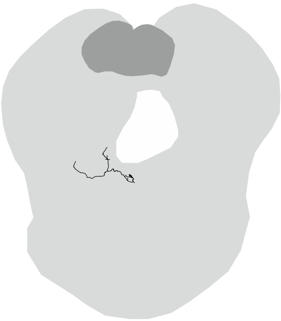

	synapses to	synapses from
hugin-PC right 1	0	0
hugin-PC right 2	0	0
hugin-PC right 3	0	0
hugin-PC right 4	0	0
hugin-PC left 1	0	0
hugin-PC left 2	0	0
hugin-PC left 3	0	0
hugin-PC left 4	0	0
hugin-RG right 1	0	0
hugin-RG right 2	0	0
hugin-RG left 1	0	0
hugin-RG left 2	0	0
hugin-VNC right 1	2	1
hugin-VNC right 2	0	0
hugin-VNC left 1	1	0
hugin-VNC left 2	0	0
hugin-PH right 1	0	0
hugin-PH right 2	0	0
hugin-PH left 1	0	0
hugin-PH left 2	0	0

9.

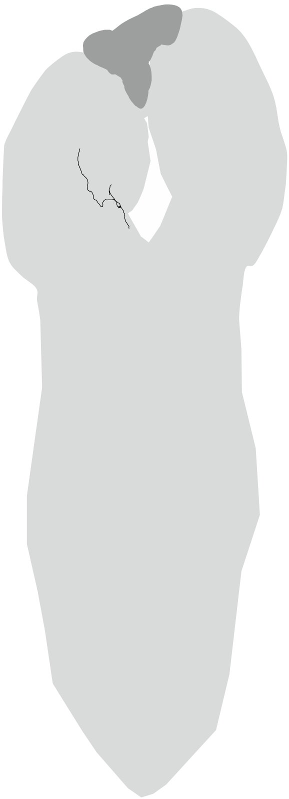

#15770032

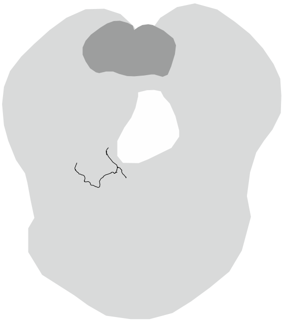

	synapses to	synapses from
hugin-PC right 1	0	0
hugin-PC right 2	0	0
hugin-PC right 3	0	0
hugin-PC right 4	0	0
hugin-PC left 1	0	0
hugin-PC left 2	0	0
hugin-PC left 3	0	0
hugin-PC left 4	0	0
hugin-RG right 1	0	0
hugin-RG right 2	0	0
hugin-RG left 1	0	0
hugin-RG left 2	0	0
hugin-VNC right 1	0	0
hugin-VNC right 2	2	0
hugin-VNC left 1	0	0
hugin-VNC left 2	0	0
hugin-PH right 1	0	0
hugin-PH right 2	0	0
hugin-PH left 1	0	0
hugin-PH left 2	0	0

10.

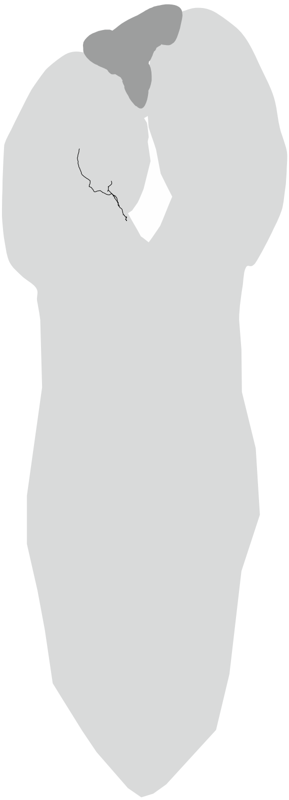

#1367171

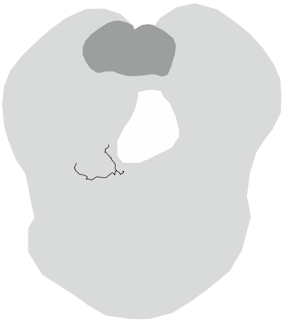

	synapses to	synapses from
hugin-PC right 1	0	0
hugin-PC right 2	0	0
hugin-PC right 3	0	0
hugin-PC right 4	0	0
hugin-PC left 1	0	0
hugin-PC left 2	0	0
hugin-PC left 3	0	0
hugin-PC left 4	0	0
hugin-RG right 1	0	0
hugin-RG right 2	0	0
hugin-RG left 1	0	0
hugin-RG left 2	0	0
hugin-VNC right 1	3	0
hugin-VNC right 2	1	0
hugin-VNC left 1	3	0
hugin-VNC left 2	0	0
hugin-PH right 1	0	0
hugin-PH right 2	0	0
hugin-PH left 1	0	0
hugin-PH left 2	0	0

11.

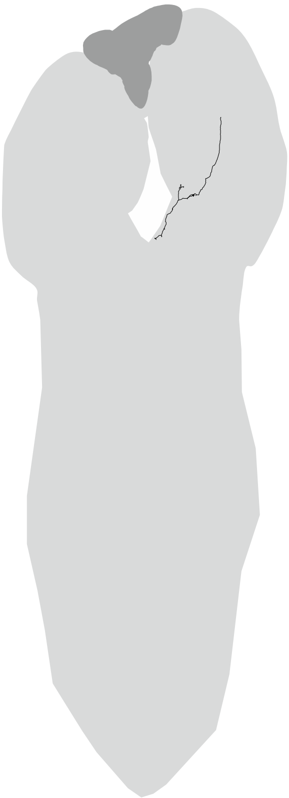

#15562137

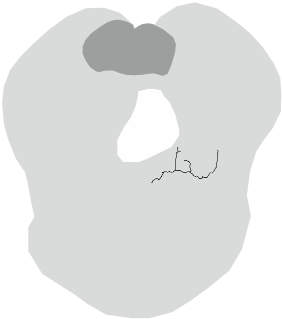

	synapses to	synapses from
hugin-PC right 1	0	0
hugin-PC right 2	0	0
hugin-PC right 3	1	0
hugin-PC right 4	0	0
hugin-PC left 1	0	0
hugin-PC left 2	0	0
hugin-PC left 3	0	0
hugin-PC left 4	0	0
hugin-RG right 1	0	0
hugin-RG right 2	0	0
hugin-RG left 1	0	0
hugin-RG left 2	0	0
hugin-VNC right 1	0	0
hugin-VNC right 2	0	0
hugin-VNC left 1	0	0
hugin-VNC left 2	1	0
hugin-PH right 1	0	0
hugin-PH right 2	0	0
hugin-PH left 1	0	0
hugin-PH left 2	0	0

12.

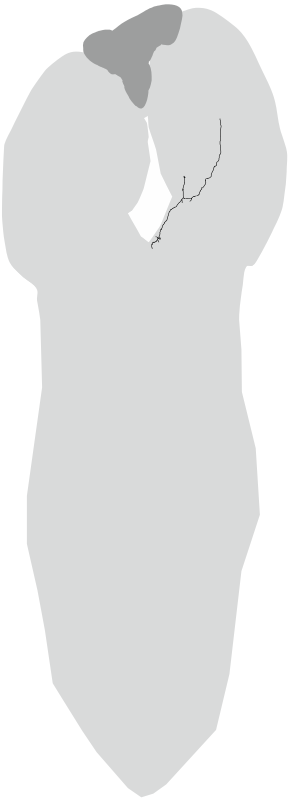

#15574231

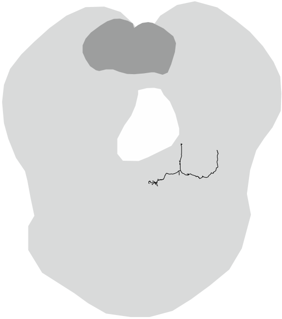

	synapses to	synapses from
hugin-PC right 1	1	1
hugin-PC right 2	5	0
hugin-PC right 3	0	0
hugin-PC right 4	2	0
hugin-PC left 1	0	0
hugin-PC left 2	0	0
hugin-PC left 3	0	0
hugin-PC left 4	0	0
hugin-RG right 1	0	0
hugin-RG right 2	0	0
hugin-RG left 1	0	0
hugin-RG left 2	0	0
hugin-VNC right 1	0	0
hugin-VNC right 2	0	0
hugin-VNC left 1	0	0
hugin-VNC left 2	0	0
hugin-PH right 1	0	0
hugin-PH right 2	0	0
hugin-PH left 1	0	0
hugin-PH left 2	0	0

13.

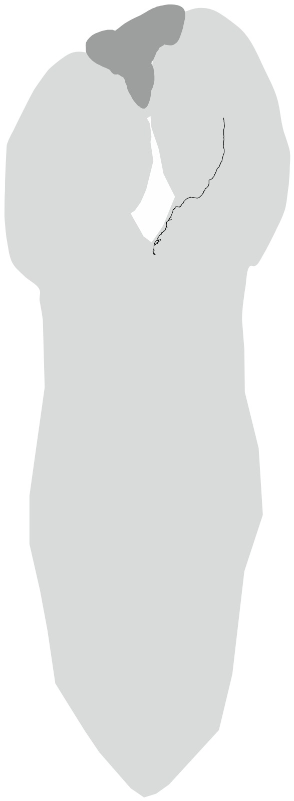

#15573396

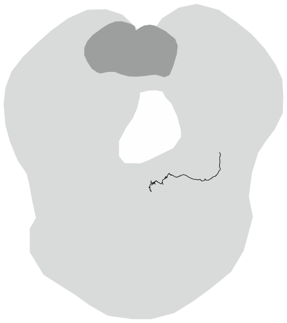

	synapses to	synapses from
hugin-PC right 1	3	0
hugin-PC right 2	0	0
hugin-PC right 3	0	0
hugin-PC right 4	0	0
hugin-PC left 1	0	0
hugin-PC left 2	0	0
hugin-PC left 3	0	0
hugin-PC left 4	0	0
hugin-RG right 1	0	0
hugin-RG right 2	0	0
hugin-RG left 1	0	0
hugin-RG left 2	0	0
hugin-VNC right 1	0	0
hugin-VNC right 2	0	0
hugin-VNC left 1	0	0
hugin-VNC left 2	2	0
hugin-PH right 1	0	0
hugin-PH right 2	0	0
hugin-PH left 1	0	0
hugin-PH left 2	0	0

14.

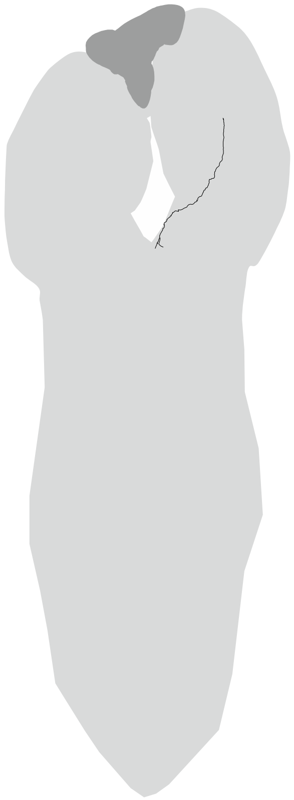

#15573450

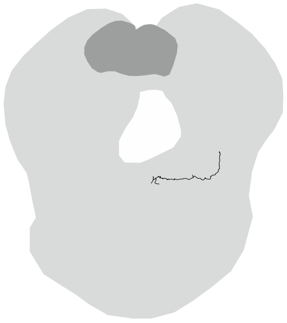

	synapses to	synapses from
hugin-PC right 1	0	0
hugin-PC right 2	0	0
hugin-PC right 3	0	0
hugin-PC right 4	0	0
hugin-PC left 1	0	0
hugin-PC left 2	0	0
hugin-PC left 3	0	0
hugin-PC left 4	0	0
hugin-RG right 1	0	0
hugin-RG right 2	0	0
hugin-RG left 1	0	0
hugin-RG left 2	0	0
hugin-VNC right 1	0	0
hugin-VNC right 2	0	0
hugin-VNC left 1	0	0
hugin-VNC left 2	2	0
hugin-PH right 1	0	0
hugin-PH right 2	0	0
hugin-PH left 1	0	0
hugin-PH left 2	0	0

AN sensory neurons - C2

1.

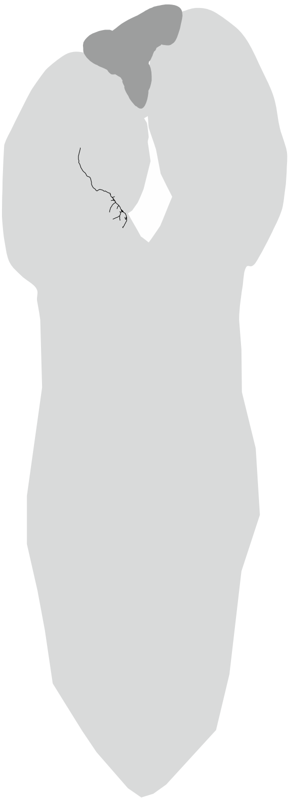

#1375101

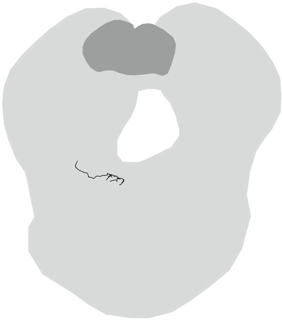

	synapses to	synapses from
hugin-PC right 1	0	0
hugin-PC right 2	0	0
hugin-PC right 3	0	0
hugin-PC right 4	0	0
hugin-PC left 1	0	0
hugin-PC left 2	0	0
hugin-PC left 3	0	0
hugin-PC left 4	2	0
hugin-RG right 1	0	0
hugin-RG right 2	0	0
hugin-RG left 1	0	0
hugin-RG left 2	0	0
hugin-VNC right 1	0	0
hugin-VNC right 2	0	0
hugin-VNC left 1	0	0
hugin-VNC left 2	0	0
hugin-PH right 1	0	0
hugin-PH right 2	0	0
hugin-PH left 1	0	0
hugin-PH left 2	0	0

2.

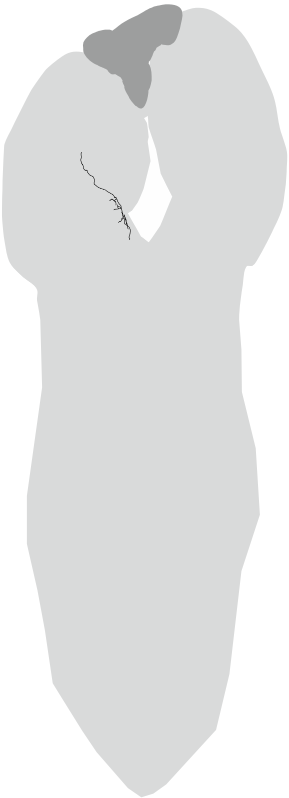

#1435466

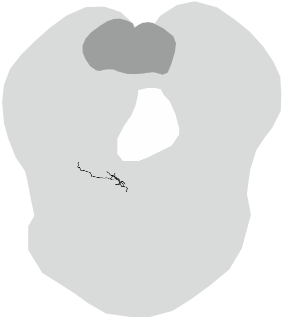

	synapses to	synapses from
hugin-PC right 1	0	0
hugin-PC right 2	0	0
hugin-PC right 3	0	0
hugin-PC right 4	0	0
hugin-PC left 1	3	0
hugin-PC left 2	1	0
hugin-PC left 3	2	0
hugin-PC left 4	0	0
hugin-RG right 1	0	0
hugin-RG right 2	0	0
hugin-RG left 1	0	0
hugin-RG left 2	0	0
hugin-VNC right 1	0	0
hugin-VNC right 2	1	0
hugin-VNC left 1	0	0
hugin-VNC left 2	0	0
hugin-PH right 1	0	0
hugin-PH right 2	0	0
hugin-PH left 1	0	0
hugin-PH left 2	0	0

3.

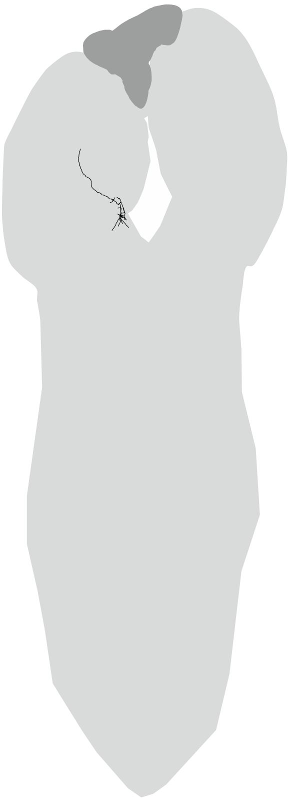

#15769680

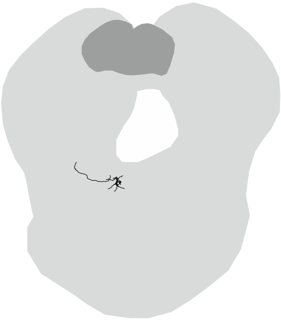

	synapses to	synapses from
hugin-PC right 1	0	0
hugin-PC right 2	0	0
hugin-PC right 3	0	0
hugin-PC right 4	0	0
hugin-PC left 1	1	0
hugin-PC left 2	0	0
hugin-PC left 3	2	0
hugin-PC left 4	2	0
hugin-RG right 1	0	0
hugin-RG right 2	0	0
hugin-RG left 1	0	0
hugin-RG left 2	0	0
hugin-VNC right 1	0	0
hugin-VNC right 2	0	0
hugin-VNC left 1	0	0
hugin-VNC left 2	0	0
hugin-PH right 1	0	0
hugin-PH right 2	0	0
hugin-PH left 1	0	0
hugin-PH left 2	0	0

4.

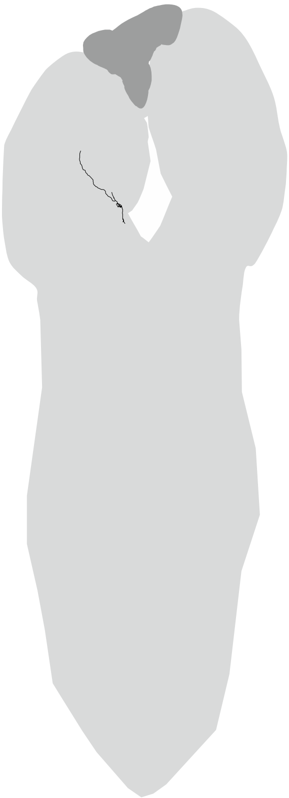

#1429969

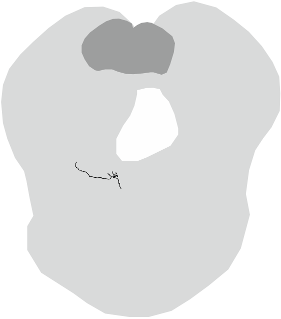

	synapses to	synapses from
hugin-PC right 1	0	0
hugin-PC right 2	0	0
hugin-PC right 3	0	0
hugin-PC right 4	0	0
hugin-PC left 1	0	0
hugin-PC left 2	1	0
hugin-PC left 3	2	0
hugin-PC left 4	1	0
hugin-RG right 1	0	0
hugin-RG right 2	0	0
hugin-RG left 1	0	0
hugin-RG left 2	0	0
hugin-VNC right 1	0	0
hugin-VNC right 2	0	0
hugin-VNC left 1	0	0
hugin-VNC left 2	0	0
hugin-PH right 1	0	0
hugin-PH right 2	0	0
hugin-PH left 1	0	0
hugin-PH left 2	0	0

5.

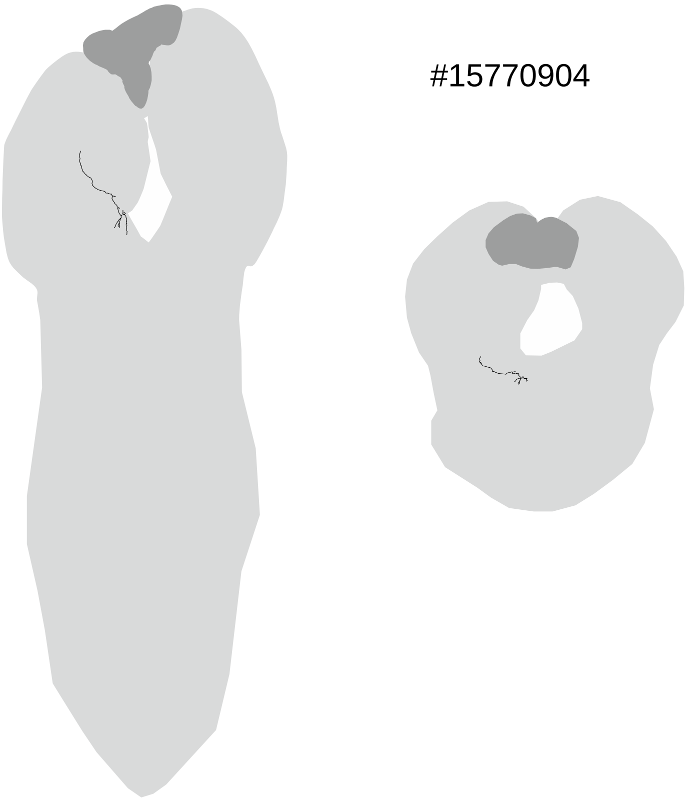

#15770904

	synapses to	synapses from
hugin-PC right 1	0	0
hugin-PC right 2	0	0
hugin-PC right 3	0	0
hugin-PC right 4	0	0
hugin-PC left 1	1	0
hugin-PC left 2	0	0
hugin-PC left 3	2	0
hugin-PC left 4	1	0
hugin-RG right 1	0	0
hugin-RG right 2	0	0
hugin-RG left 1	0	0
hugin-RG left 2	0	0
hugin-VNC right 1	0	0
hugin-VNC right 2	0	0
hugin-VNC left 1	0	0
hugin-VNC left 2	0	0
hugin-PH right 1	0	0
hugin-PH right 2	0	0
hugin-PH left 1	0	0
hugin-PH left 2	0	0

6.

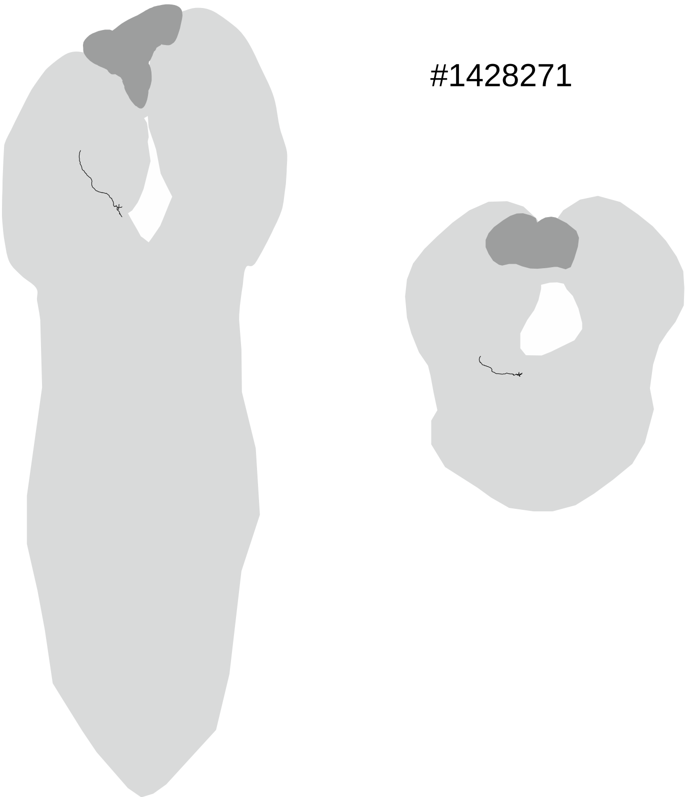

#1428271

	synapses to	synapses from
hugin-PC right 1	0	0
hugin-PC right 2	0	0
hugin-PC right 3	0	0
hugin-PC right 4	0	0
hugin-PC left 1	4	0
hugin-PC left 2	0	0
hugin-PC left 3	0	0
hugin-PC left 4	1	0
hugin-RG right 1	0	0
hugin-RG right 2	0	0
hugin-RG left 1	0	0
hugin-RG left 2	0	0
hugin-VNC right 1	0	0
hugin-VNC right 2	1	0
hugin-VNC left 1	0	0
hugin-VNC left 2	0	0
hugin-PH right 1	0	0
hugin-PH right 2	0	0
hugin-PH left 1	0	0
hugin-PH left 2	0	0

7.

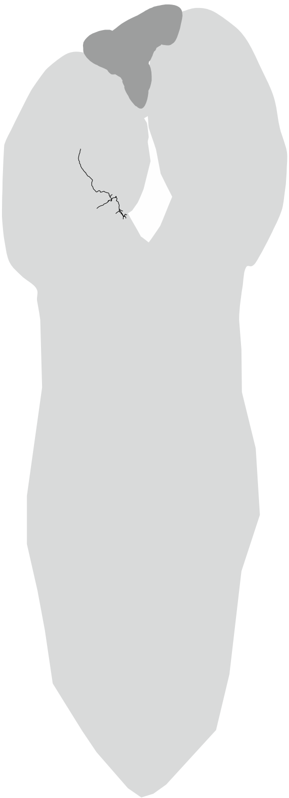

#1414206

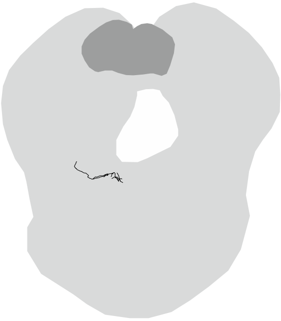

	synapses to	synapses from
hugin-PC right 1	0	0
hugin-PC right 2	0	0
hugin-PC right 3	0	0
hugin-PC right 4	0	0
hugin-PC left 1	0	0
hugin-PC left 2	0	0
hugin-PC left 3	0	0
hugin-PC left 4	0	2
hugin-RG right 1	0	0
hugin-RG right 2	0	0
hugin-RG left 1	0	0
hugin-RG left 2	0	0
hugin-VNC right 1	0	0
hugin-VNC right 2	2	0
hugin-VNC left 1	0	0
hugin-VNC left 2	0	0
hugin-PH right 1	0	0
hugin-PH right 2	0	0
hugin-PH left 1	0	0
hugin-PH left 2	0	0

8.

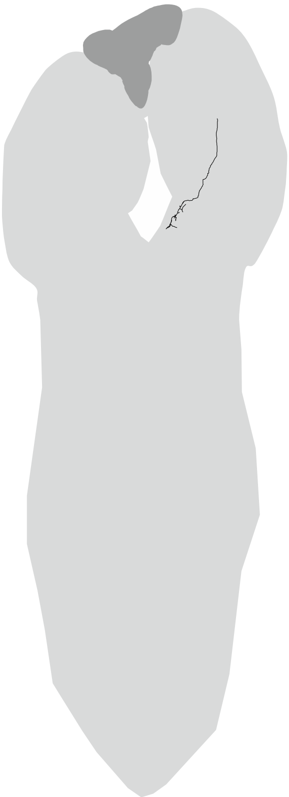

#15575327

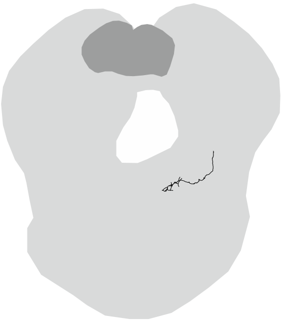

	synapses to	synapses from
hugin-PC right 1	3	0
hugin-PC right 2	2	0
hugin-PC right 3	1	0
hugin-PC right 4	0	0
hugin-PC left 1	0	0
hugin-PC left 2	0	0
hugin-PC left 3	0	0
hugin-PC left 4	0	0
hugin-RG right 1	0	0
hugin-RG right 2	0	0
hugin-RG left 1	0	0
hugin-RG left 2	0	0
hugin-VNC right 1	0	0
hugin-VNC right 2	0	0
hugin-VNC left 1	0	0
hugin-VNC left 2	0	0
hugin-PH right 1	0	0
hugin-PH right 2	0	0
hugin-PH left 1	0	0
hugin-PH left 2	0	0

9.

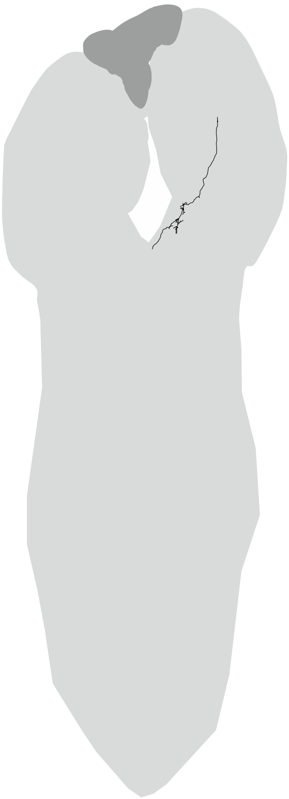

#15564706

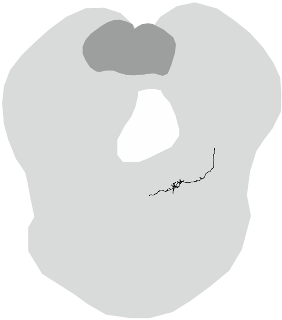

	synapses to	synapses from
hugin-PC right 1	0	0
hugin-PC right 2	1	0
hugin-PC right 3	1	0
hugin-PC right 4	1	0
hugin-PC left 1	0	0
hugin-PC left 2	0	0
hugin-PC left 3	0	0
hugin-PC left 4	0	0
hugin-RG right 1	0	0
hugin-RG right 2	0	0
hugin-RG left 1	0	0
hugin-RG left 2	0	0
hugin-VNC right 1	0	0
hugin-VNC right 2	0	0
hugin-VNC left 1	0	0
hugin-VNC left 2	0	0
hugin-PH right 1	0	0
hugin-PH right 2	0	0
hugin-PH left 1	0	0
hugin-PH left 2	0	0

10.

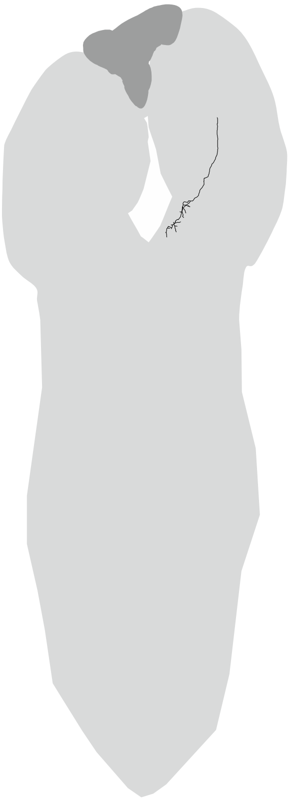

#15564782

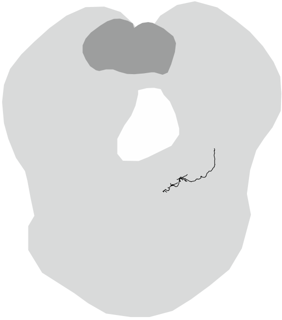

	synapses to	synapses from
hugin-PC right 1	3	0
hugin-PC right 2	0	0
hugin-PC right 3	0	0
hugin-PC right 4	0	0
hugin-PC left 1	0	0
hugin-PC left 2	0	0
hugin-PC left 3	0	0
hugin-PC left 4	0	0
hugin-RG right 1	0	0
hugin-RG right 2	0	0
hugin-RG left 1	0	0
hugin-RG left 2	0	0
hugin-VNC right 1	0	0
hugin-VNC right 2	0	0
hugin-VNC left 1	0	0
hugin-VNC left 2	0	0
hugin-PH right 1	0	0
hugin-PH right 2	0	0
hugin-PH left 1	0	0
hugin-PH left 2	0	0

11.

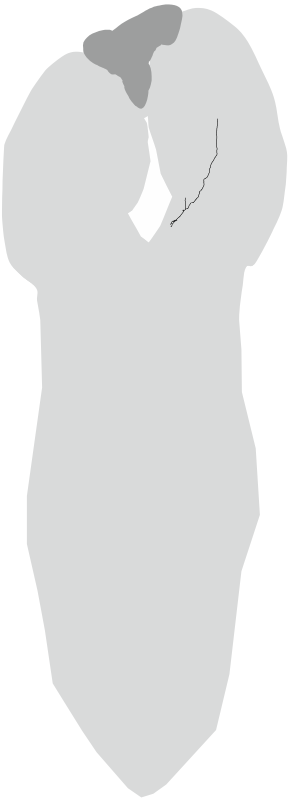

#15572414

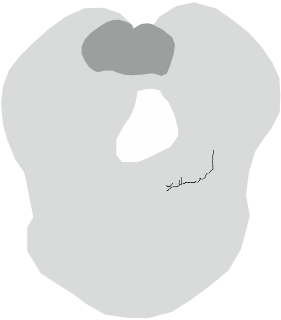

	synapses to	synapses from
hugin-PC right 1	1	0
hugin-PC right 2	1	0
hugin-PC right 3	0	0
hugin-PC right 4	0	0
hugin-PC left 1	0	0
hugin-PC left 2	0	0
hugin-PC left 3	0	0
hugin-PC left 4	0	0
hugin-RG right 1	0	0
hugin-RG right 2	0	0
hugin-RG left 1	0	0
hugin-RG left 2	0	0
hugin-VNC right 1	0	0
hugin-VNC right 2	0	0
hugin-VNC left 1	0	0
hugin-VNC left 2	0	0
hugin-PH right 1	0	0
hugin-PH right 2	0	0
hugin-PH left 1	0	0
hugin-PH left 2	0	0

12.

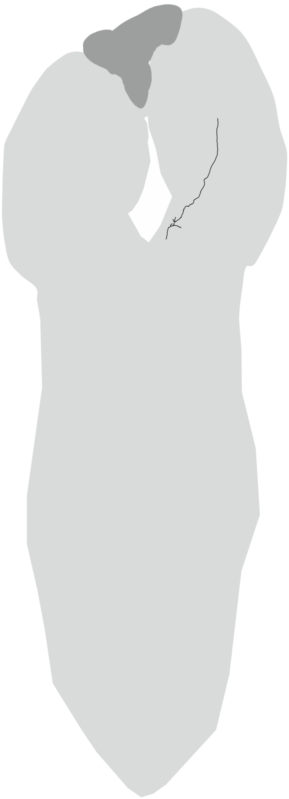

#15564807

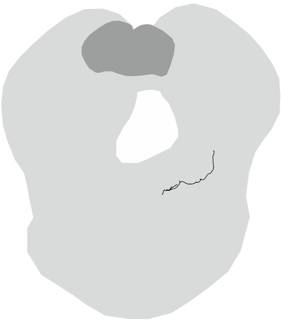

	synapses to	synapses from
hugin-PC right 1	2	0
hugin-PC right 2	1	0
hugin-PC right 3	3	0
hugin-PC right 4	0	0
hugin-PC left 1	0	0
hugin-PC left 2	0	0
hugin-PC left 3	0	0
hugin-PC left 4	0	0
hugin-RG right 1	0	0
hugin-RG right 2	0	0
hugin-RG left 1	0	0
hugin-RG left 2	0	0
hugin-VNC right 1	0	0
hugin-VNC right 2	0	0
hugin-VNC left 1	0	0
hugin-VNC left 2	0	0
hugin-PH right 1	0	0
hugin-PH right 2	0	0
hugin-PH left 1	0	0
hugin-PH left 2	0	0

13.

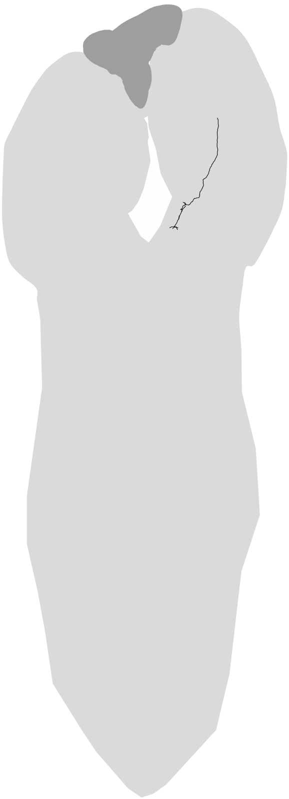

#15543068

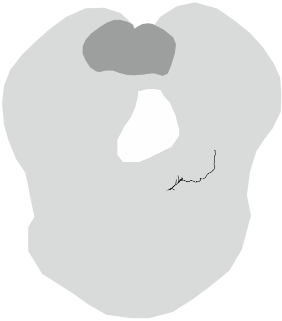

	synapses to	synapses from
hugin-PC right 1	1	0
hugin-PC right 2	0	0
hugin-PC right 3	2	0
hugin-PC right 4	1	0
hugin-PC left 1	0	0
hugin-PC left 2	0	0
hugin-PC left 3	0	0
hugin-PC left 4	0	0
hugin-RG right 1	0	0
hugin-RG right 2	0	0
hugin-RG left 1	0	0
hugin-RG left 2	0	0
hugin-VNC right 1	0	0
hugin-VNC right 2	0	0
hugin-VNC left 1	0	0
hugin-VNC left 2	0	0
hugin-PH right 1	0	0
hugin-PH right 2	0	0
hugin-PH left 1	0	0
hugin-PH left 2	0	0

14.

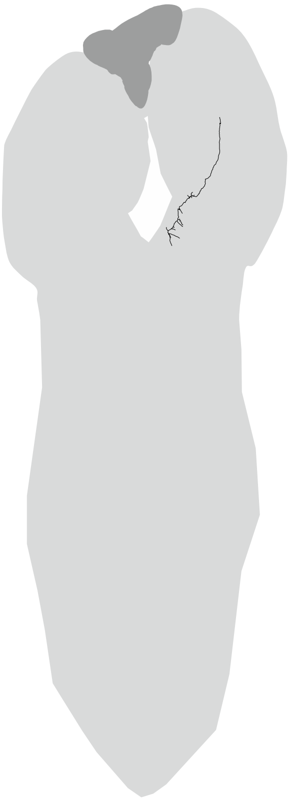

#15574328

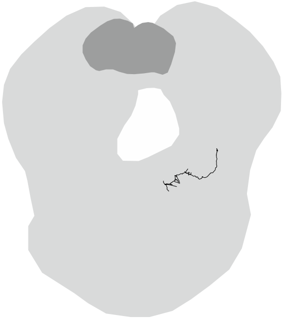

	synapses to	synapses from
hugin-PC right 1	3	1
hugin-PC right 2	1	0
hugin-PC right 3	0	0
hugin-PC right 4	3	1
hugin-PC left 1	0	0
hugin-PC left 2	0	0
hugin-PC left 3	0	0
hugin-PC left 4	0	0
hugin-RG right 1	0	0
hugin-RG right 2	0	0
hugin-RG left 1	0	0
hugin-RG left 2	0	0
hugin-VNC right 1	0	0
hugin-VNC right 2	0	0
hugin-VNC left 1	0	0
hugin-VNC left 2	0	0
hugin-PH right 1	0	0
hugin-PH right 2	0	0
hugin-PH left 1	0	0
hugin-PH left 2	0	0

15.

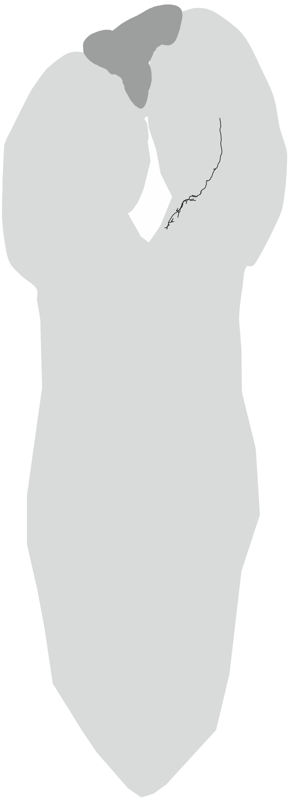

#6883831

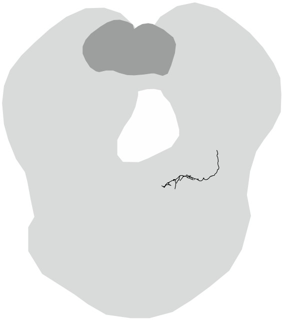

	synapses to	synapses from
hugin-PC right 1	2	2
hugin-PC right 2	0	0
hugin-PC right 3	0	0
hugin-PC right 4	0	0
hugin-PC left 1	0	0
hugin-PC left 2	0	0
hugin-PC left 3	0	0
hugin-PC left 4	0	0
hugin-RG right 1	0	0
hugin-RG right 2	0	0
hugin-RG left 1	0	0
hugin-RG left 2	0	0
hugin-VNC right 1	0	0
hugin-VNC right 2	0	0
hugin-VNC left 1	0	0
hugin-VNC left 2	0	0
hugin-PH right 1	0	0
hugin-PH right 2	0	0
hugin-PH left 1	0	0
hugin-PH left 2	0	0

16.

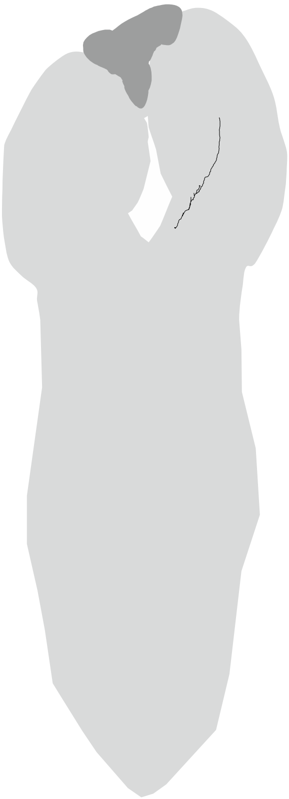

#9716858

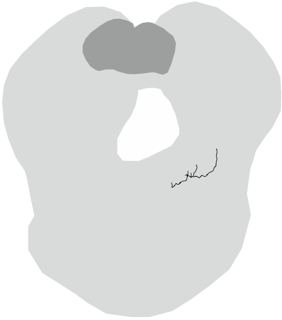

	synapses to	synapses from
hugin-PC right 1	0	0
hugin-PC right 2	3	0
hugin-PC right 3	0	0
hugin-PC right 4	0	0
hugin-PC left 1	0	0
hugin-PC left 2	0	0
hugin-PC left 3	0	0
hugin-PC left 4	0	0
hugin-RG right 1	0	0
hugin-RG right 2	0	0
hugin-RG left 1	0	0
hugin-RG left 2	0	0
hugin-VNC right 1	0	0
hugin-VNC right 2	0	0
hugin-VNC left 1	0	0
hugin-VNC left 2	0	0
hugin-PH right 1	0	0
hugin-PH right 2	0	0
hugin-PH left 1	0	0
hugin-PH left 2	0	0

17.

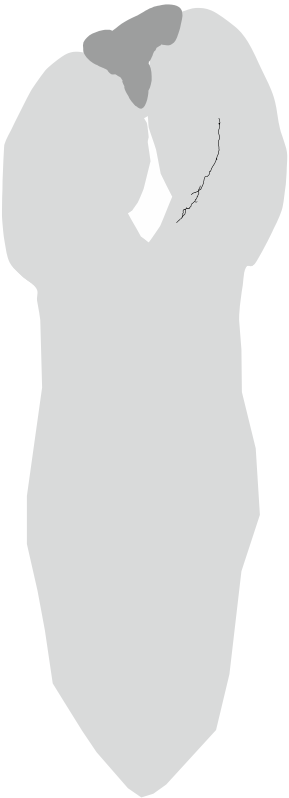

#9748269

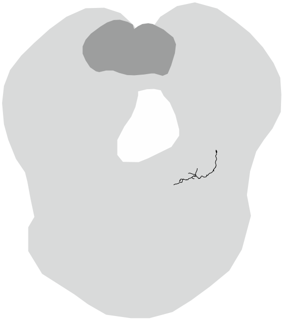

	synapses to	synapses from
hugin-PC right 1	0	0
hugin-PC right 2	0	0
hugin-PC right 3	0	0
hugin-PC right 4	2	0
hugin-PC left 1	0	0
hugin-PC left 2	0	0
hugin-PC left 3	0	0
hugin-PC left 4	0	0
hugin-RG right 1	0	0
hugin-RG right 2	0	0
hugin-RG left 1	0	0
hugin-RG left 2	0	0
hugin-VNC right 1	0	0
hugin-VNC right 2	0	0
hugin-VNC left 1	0	0
hugin-VNC left 2	0	0
hugin-PH right 1	0	0
hugin-PH right 2	0	0
hugin-PH left 1	0	0
hugin-PH left 2	0	0

18.

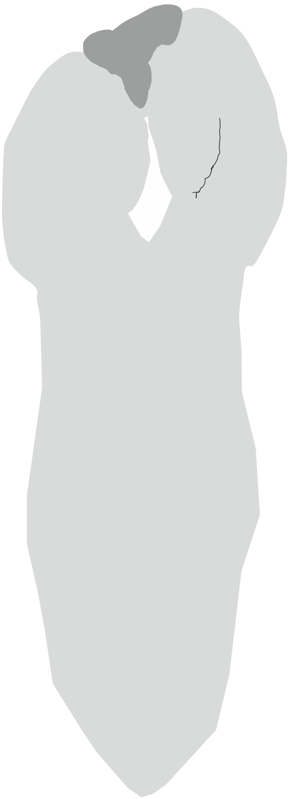

#16442344

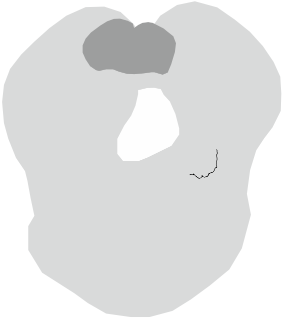

	synapses to	synapses from
hugin-PC right 1	0	0
hugin-PC right 2	2	0
hugin-PC right 3	0	0
hugin-PC right 4	0	0
hugin-PC left 1	0	0
hugin-PC left 2	0	0
hugin-PC left 3	0	0
hugin-PC left 4	0	0
hugin-RG right 1	0	0
hugin-RG right 2	0	0
hugin-RG left 1	0	0
hugin-RG left 2	0	0
hugin-VNC right 1	0	0
hugin-VNC right 2	0	0
hugin-VNC left 1	0	0
hugin-VNC left 2	0	0
hugin-PH right 1	0	0
hugin-PH right 2	0	0
hugin-PH left 1	0	0
hugin-PH left 2	0	0

AN sensory neurons - C3

1.

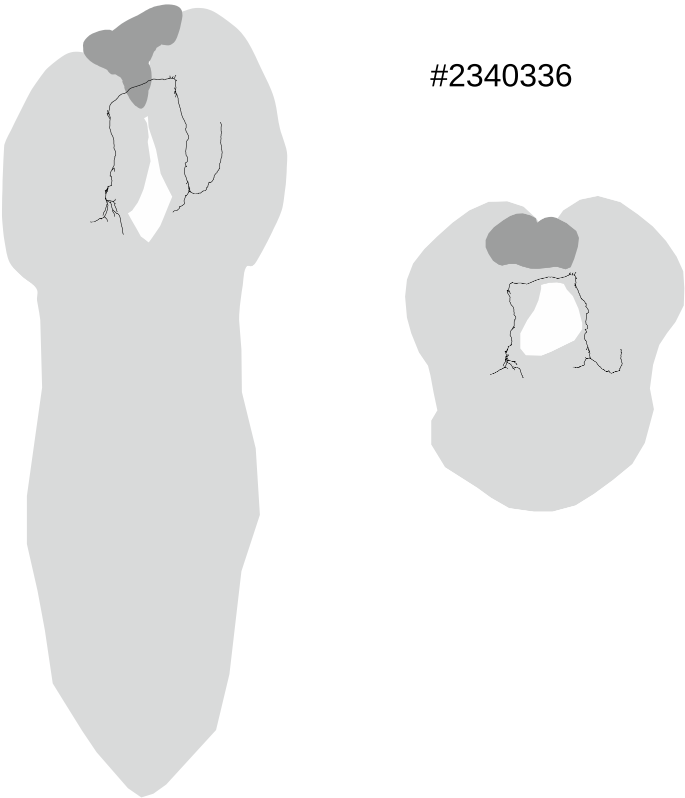

#2340336

	synapses to	synapses from
hugin-PC right 1	1	1
hugin-PC right 2	1	0
hugin-PC right 3	1	0
hugin-PC right 4	1	0
hugin-PC left 1	0	0
hugin-PC left 2	1	0
hugin-PC left 3	1	0
hugin-PC left 4	0	0
hugin-RG right 1	0	0
hugin-RG right 2	0	0
hugin-RG left 1	0	0
hugin-RG left 2	0	0
hugin-VNC right 1	0	0
hugin-VNC right 2	0	0
hugin-VNC left 1	0	0
hugin-VNC left 2	0	0
hugin-PH right 1	0	0
hugin-PH right 2	0	0
hugin-PH left 1	0	0
hugin-PH left 2	0	0

2.

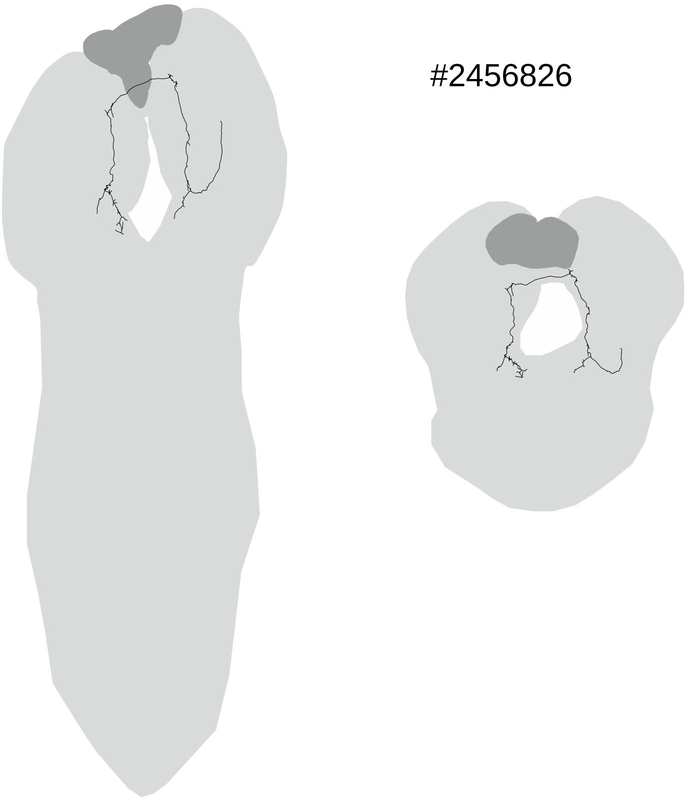

#2456826

	synapses to	synapses from
hugin-PC right 1	1	0
hugin-PC right 2	1	0
hugin-PC right 3	4	0
hugin-PC right 4	1	0
hugin-PC left 1	0	0
hugin-PC left 2	1	0
hugin-PC left 3	0	1
hugin-PC left 4	2	0
hugin-RG right 1	0	0
hugin-RG right 2	0	0
hugin-RG left 1	0	0
hugin-RG left 2	0	0
hugin-VNC right 1	0	0
hugin-VNC right 2	0	0
hugin-VNC left 1	0	0
hugin-VNC left 2	0	0
hugin-PH right 1	0	0
hugin-PH right 2	0	0
hugin-PH left 1	0	0
hugin-PH left 2	0	0

3.

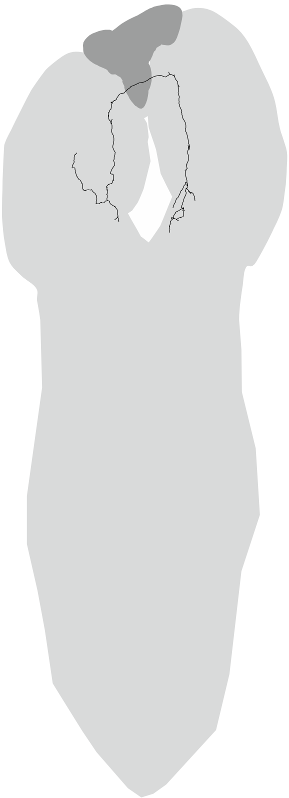

#13744425

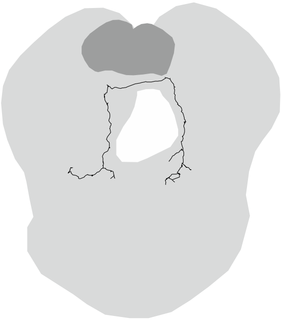

	synapses to	synapses from
hugin-PC right 1	0	2
hugin-PC right 2	1	1
hugin-PC right 3	0	0
hugin-PC right 4	0	0
hugin-PC left 1	2	0
hugin-PC left 2	0	0
hugin-PC left 3	2	0
hugin-PC left 4	1	0
hugin-RG right 1	0	0
hugin-RG right 2	0	0
hugin-RG left 1	0	0
hugin-RG left 2	0	0
hugin-VNC right 1	0	0
hugin-VNC right 2	0	0
hugin-VNC left 1	0	0
hugin-VNC left 2	0	0
hugin-PH right 1	0	0
hugin-PH right 2	0	0
hugin-PH left 1	0	0
hugin-PH left 2	0	0

4.

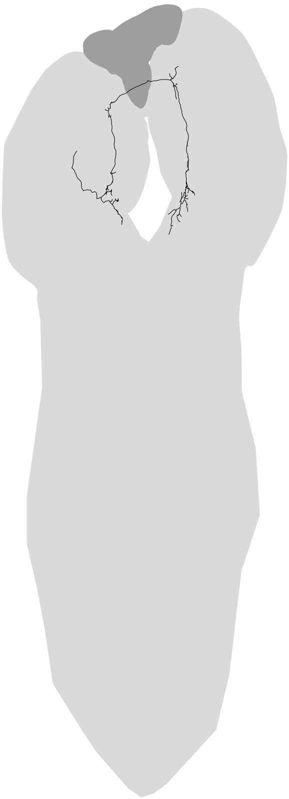

#2284021

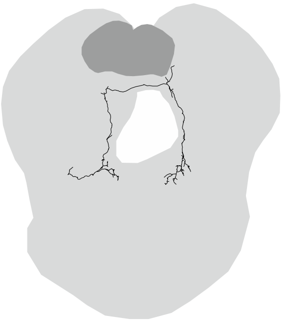

	synapses to	synapses from
hugin-PC right 1	1	1
hugin-PC right 2	0	1
hugin-PC right 3	1	0
hugin-PC right 4	2	0
hugin-PC left 1	1	0
hugin-PC left 2	0	0
hugin-PC left 3	1	1
hugin-PC left 4	7	0
hugin-RG right 1	0	0
hugin-RG right 2	0	0
hugin-RG left 1	0	0
hugin-RG left 2	0	0
hugin-VNC right 1	0	0
hugin-VNC right 2	0	0
hugin-VNC left 1	0	0
hugin-VNC left 2	0	0
hugin-PH right 1	0	0
hugin-PH right 2	0	0
hugin-PH left 1	0	0
hugin-PH left 2	0	0

AN sensory neurons - C4

1.

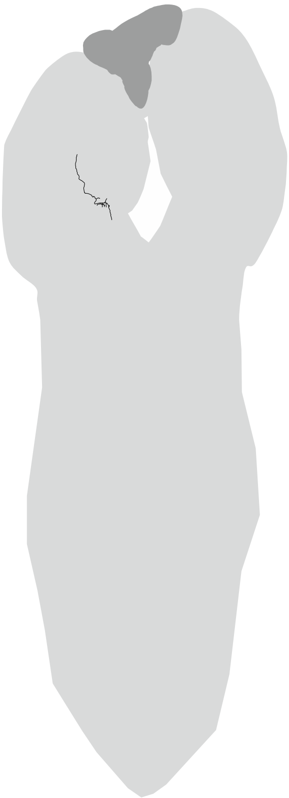

#15998753

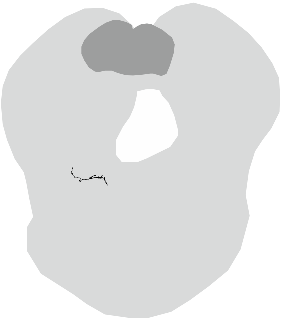

	synapses to	synapses from
hugin-PC right 1	0	0
hugin-PC right 2	0	0
hugin-PC right 3	0	0
hugin-PC right 4	0	0
hugin-PC left 1	3	0
hugin-PC left 2	0	0
hugin-PC left 3	0	0
hugin-PC left 4	2	0
hugin-RG right 1	0	0
hugin-RG right 2	0	0
hugin-RG left 1	0	0
hugin-RG left 2	0	0
hugin-VNC right 1	0	0
hugin-VNC right 2	0	0
hugin-VNC left 1	0	0
hugin-VNC left 2	0	0
hugin-PH right 1	0	0
hugin-PH right 2	0	0
hugin-PH left 1	0	0
hugin-PH left 2	0	0

2.

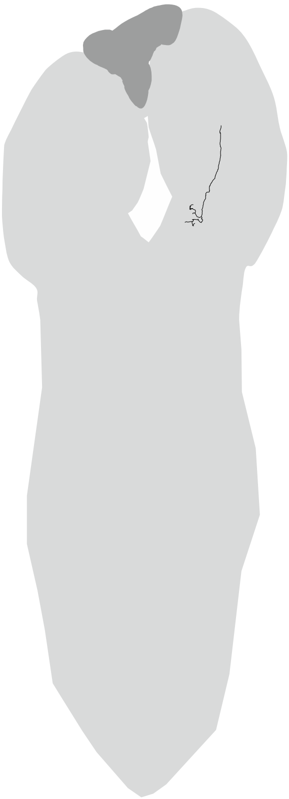

#15532105

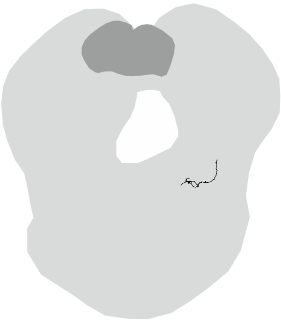

	synapses to	synapses from
hugin-PC right 1	0	0
hugin-PC right 2	0	0
hugin-PC right 3	0	0
hugin-PC right 4	3	0
hugin-PC left 1	0	0
hugin-PC left 2	0	0
hugin-PC left 3	0	0
hugin-PC left 4	0	0
hugin-RG right 1	0	0
hugin-RG right 2	0	0
hugin-RG left 1	0	0
hugin-RG left 2	0	0
hugin-VNC right 1	0	0
hugin-VNC right 2	0	0
hugin-VNC left 1	0	0
hugin-VNC left 2	0	0
hugin-PH right 1	0	0
hugin-PH right 2	0	0
hugin-PH left 1	0	0
hugin-PH left 2	0	0

3.

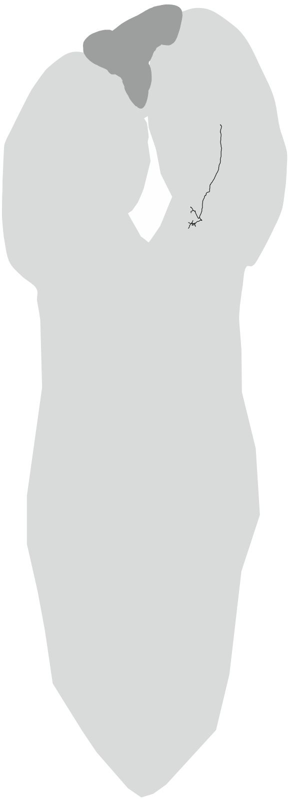

#3192952

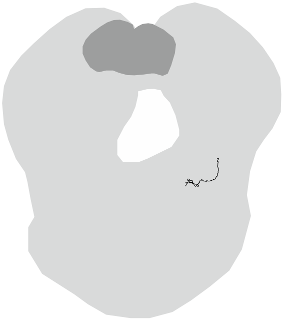

	synapses to	synapses from
hugin-PC right 1	0	0
hugin-PC right 2	0	0
hugin-PC right 3	0	0
hugin-PC right 4	2	0
hugin-PC left 1	0	0
hugin-PC left 2	0	0
hugin-PC left 3	0	0
hugin-PC left 4	0	0
hugin-RG right 1	0	0
hugin-RG right 2	0	0
hugin-RG left 1	0	0
hugin-RG left 2	0	0
hugin-VNC right 1	0	0
hugin-VNC right 2	0	0
hugin-VNC left 1	0	0
hugin-VNC left 2	0	0
hugin-PH right 1	0	0
hugin-PH right 2	0	0
hugin-PH left 1	0	0
hugin-PH left 2	0	0

AN sensory neurons - C5

1.

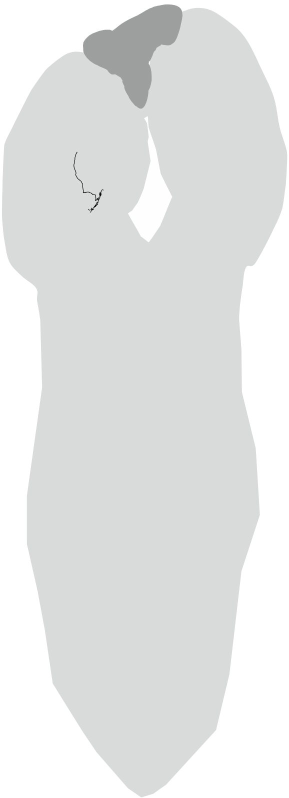

#1706716

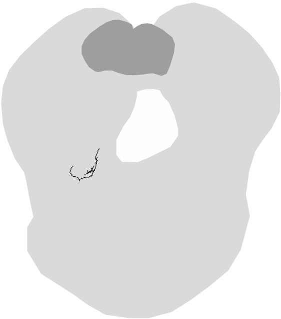

	synapses to	synapses from
hugin-PC right 1	0	0
hugin-PC right 2	0	0
hugin-PC right 3	0	0
hugin-PC right 4	0	0
hugin-PC left 1	2	0
hugin-PC left 2	0	0
hugin-PC left 3	0	0
hugin-PC left 4	0	0
hugin-RG right 1	0	0
hugin-RG right 2	0	0
hugin-RG left 1	0	0
hugin-RG left 2	0	0
hugin-VNC right 1	0	0
hugin-VNC right 2	0	0
hugin-VNC left 1	0	0
hugin-VNC left 2	0	0
hugin-PH right 1	0	0
hugin-PH right 2	0	0
hugin-PH left 1	0	0
hugin-PH left 2	0	0

2.

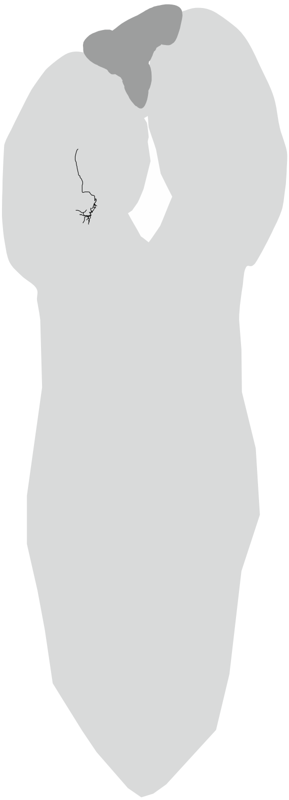

#15995929

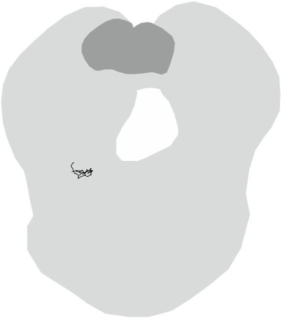

	synapses to	synapses from
hugin-PC right 1	0	0
hugin-PC right 2	0	0
hugin-PC right 3	0	0
hugin-PC right 4	0	0
hugin-PC left 1	3	0
hugin-PC left 2	0	0
hugin-PC left 3	0	0
hugin-PC left 4	0	0
hugin-RG right 1	0	0
hugin-RG right 2	0	0
hugin-RG left 1	0	0
hugin-RG left 2	0	0
hugin-VNC right 1	0	0
hugin-VNC right 2	0	0
hugin-VNC left 1	0	0
hugin-VNC left 2	0	0
hugin-PH right 1	0	0
hugin-PH right 2	0	0
hugin-PH left 1	0	0
hugin-PH left 2	0	0

3.

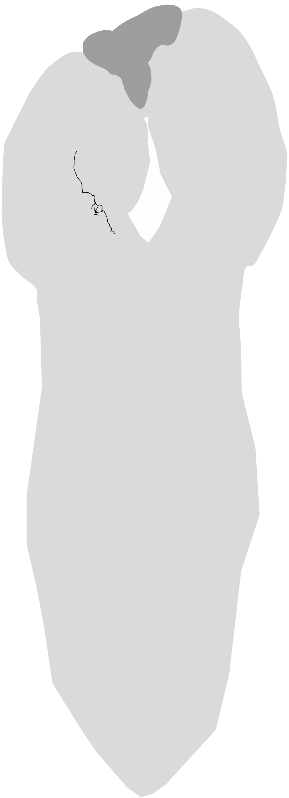

#9122616

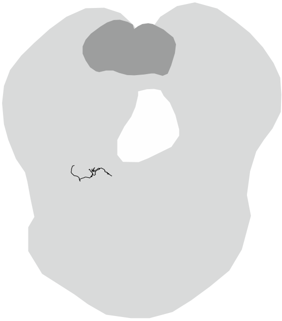

	synapses to	synapses from
hugin-PC right 1	0	0
hugin-PC right 2	0	0
hugin-PC right 3	0	0
hugin-PC right 4	0	0
hugin-PC left 1	3	0
hugin-PC left 2	0	0
hugin-PC left 3	0	0
hugin-PC left 4	0	0
hugin-RG right 1	0	0
hugin-RG right 2	0	0
hugin-RG left 1	0	0
hugin-RG left 2	0	0
hugin-VNC right 1	0	0
hugin-VNC right 2	0	0
hugin-VNC left 1	0	0
hugin-VNC left 2	0	0
hugin-PH right 1	0	0
hugin-PH right 2	0	0
hugin-PH left 1	0	0
hugin-PH left 2	0	0

4.

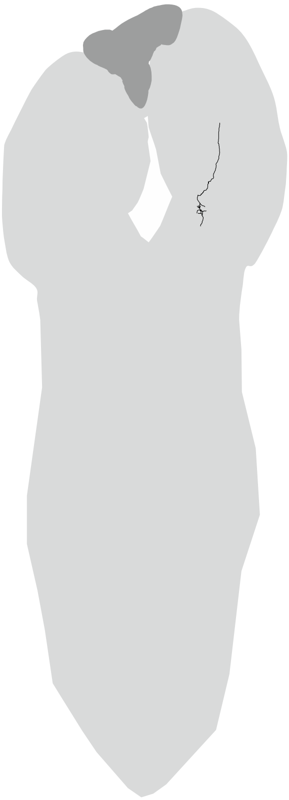

#15541976

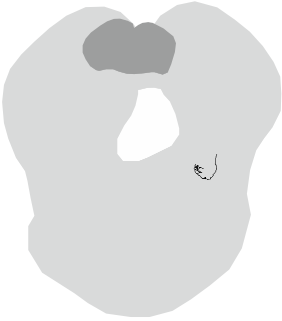

	synapses to	synapses from
hugin-PC right 1	0	0
hugin-PC right 2	0	0
hugin-PC right 3	2	0
hugin-PC right 4	0	0
hugin-PC left 1	0	0
hugin-PC left 2	0	0
hugin-PC left 3	0	0
hugin-PC left 4	0	0
hugin-RG right 1	0	0
hugin-RG right 2	0	0
hugin-RG left 1	0	0
hugin-RG left 2	0	0
hugin-VNC right 1	0	0
hugin-VNC right 2	0	0
hugin-VNC left 1	0	0
hugin-VNC left 2	0	0
hugin-PH right 1	0	0
hugin-PH right 2	0	0
hugin-PH left 1	0	0
hugin-PH left 2	0	0

5.

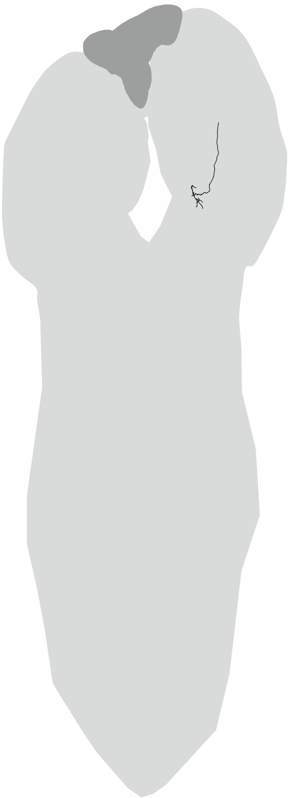

#15506094

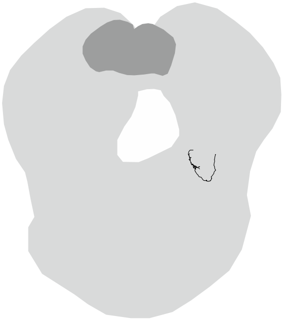

	synapses to	synapses from
hugin-PC right 1	0	0
hugin-PC right 2	0	0
hugin-PC right 3	6	0
hugin-PC right 4	0	0
hugin-PC left 1	0	0
hugin-PC left 2	0	0
hugin-PC left 3	0	0
hugin-PC left 4	0	0
hugin-RG right 1	0	0
hugin-RG right 2	0	0
hugin-RG left 1	0	0
hugin-RG left 2	0	0
hugin-VNC right 1	0	0
hugin-VNC right 2	0	0
hugin-VNC left 1	0	0
hugin-VNC left 2	0	0
hugin-PH right 1	0	0
hugin-PH right 2	0	0
hugin-PH left 1	0	0
hugin-PH left 2	0	0

AN sensory neurons - C6

1.

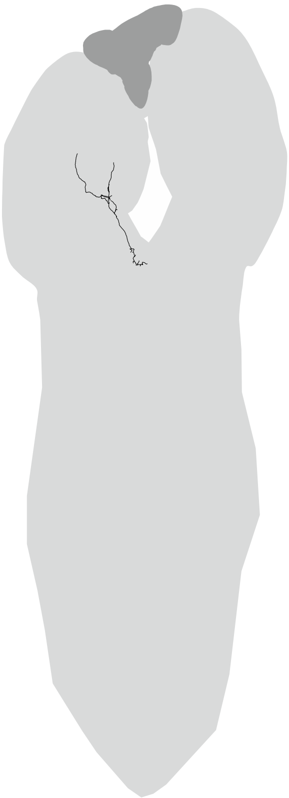

#15997666

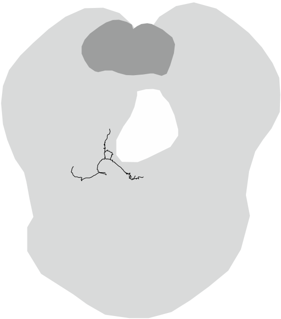

	synapses to	synapses from
hugin-PC right 1	0	0
hugin-PC right 2	0	0
hugin-PC right 3	0	0
hugin-PC right 4	0	0
hugin-PC left 1	0	0
hugin-PC left 2	0	0
hugin-PC left 3	1	0
hugin-PC left 4	0	0
hugin-RG right 1	1	0
hugin-RG right 2	0	0
hugin-RG left 1	2	0
hugin-RG left 2	0	0
hugin-VNC right 1	0	0
hugin-VNC right 2	0	0
hugin-VNC left 1	0	0
hugin-VNC left 2	0	0
hugin-PH right 1	0	0
hugin-PH right 2	0	0
hugin-PH left 1	0	0
hugin-PH left 2	0	0

2.

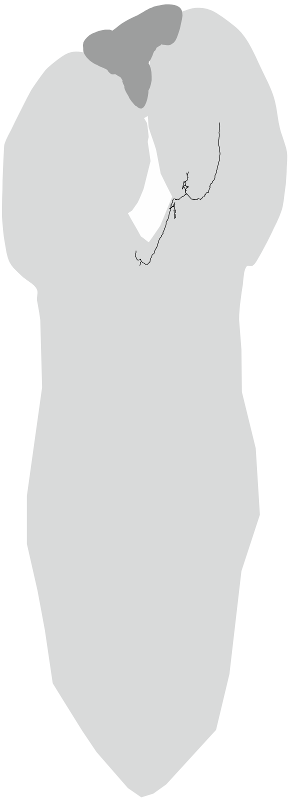

#15541944

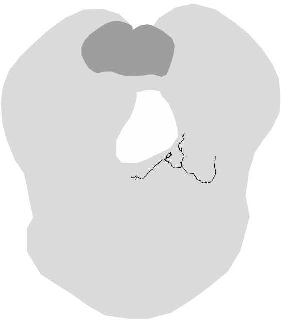

	synapses to	synapses from
hugin-PC right 1	0	0
hugin-PC right 2	0	0
hugin-PC right 3	0	0
hugin-PC right 4	0	0
hugin-PC left 1	0	0
hugin-PC left 2	0	0
hugin-PC left 3	0	0
hugin-PC left 4	0	0
hugin-RG right 1	0	0
hugin-RG right 2	0	0
hugin-RG left 1	0	0
hugin-RG left 2	1	0
hugin-VNC right 1	0	0
hugin-VNC right 2	1	0
hugin-VNC left 1	0	0
hugin-VNC left 2	0	0
hugin-PH right 1	0	0
hugin-PH right 2	0	0
hugin-PH left 1	0	0
hugin-PH left 2	0	0

I Abdominal sensory neurons

1.

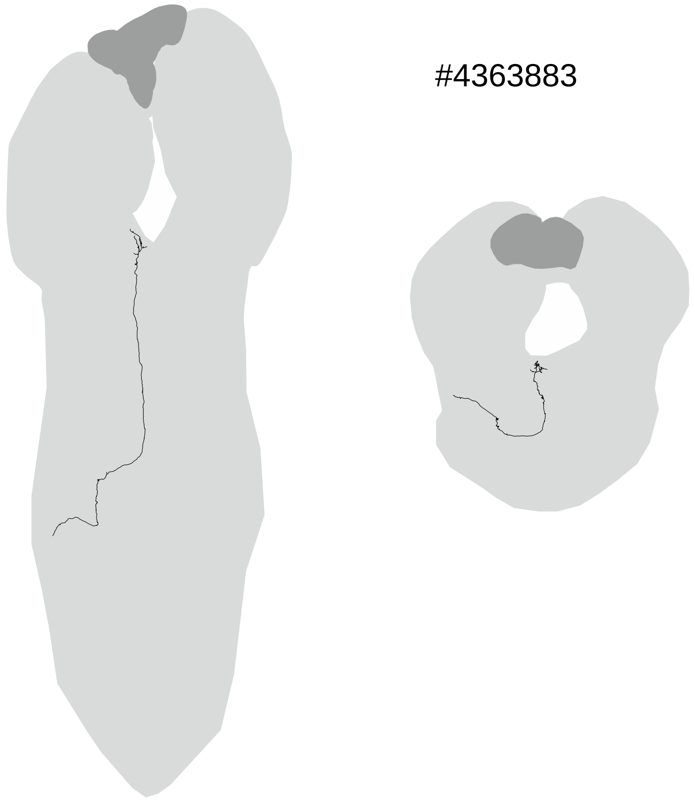

	synapses to	synapses from
hugin-PC right 1	0	0
hugin-PC right 2	0	0
hugin-PC right 3	0	0
hugin-PC right 4	0	0
hugin-PC left 1	0	0
hugin-PC left 2	0	0
hugin-PC left 3	0	0
hugin-PC left 4	0	0
hugin-RG right 1	0	0
hugin-RG right 2	0	0
hugin-RG left 1	0	0
hugin-RG left 2	0	0
hugin-VNC right 1	3	0
hugin-VNC right 2	2	0
hugin-VNC left 1	4	0
hugin-VNC left 2	0	0
hugin-PH right 1	0	0
hugin-PH right 2	0	0
hugin-PH left 1	0	0
hugin-PH left 2	0	0

2.

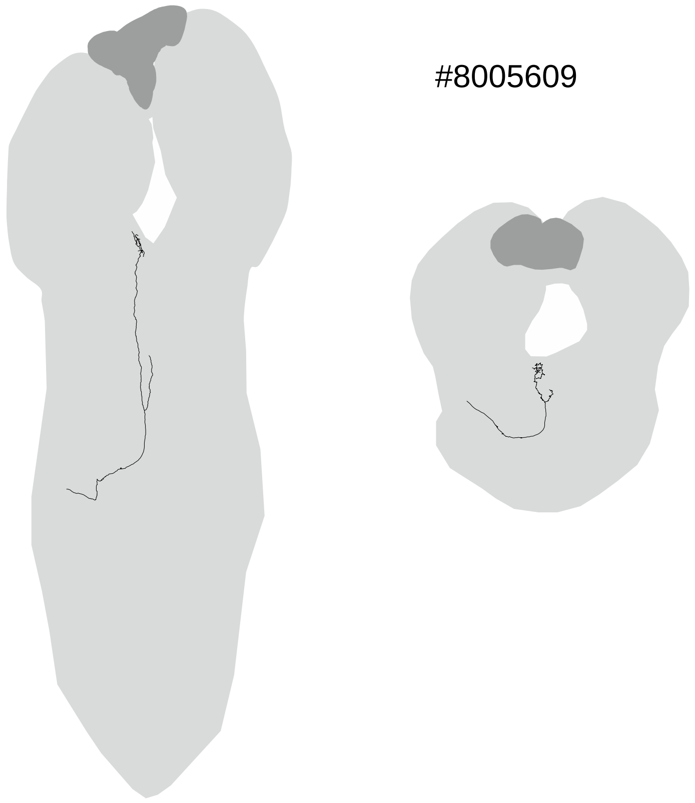

	synapses to	synapses from
hugin-PC right 1	0	0
hugin-PC right 2	0	0
hugin-PC right 3	0	0
hugin-PC right 4	0	0
hugin-PC left 1	0	0
hugin-PC left 2	0	0
hugin-PC left 3	0	0
hugin-PC left 4	0	0
hugin-RG right 1	0	0
hugin-RG right 2	0	0
hugin-RG left 1	0	0
hugin-RG left 2	0	0
hugin-VNC right 1	2	0
hugin-VNC right 2	4	0
hugin-VNC left 1	3	0
hugin-VNC left 2	0	0
hugin-PH right 1	0	0
hugin-PH right 2	0	0
hugin-PH left 1	0	0
hugin-PH left 2	0	0

3.

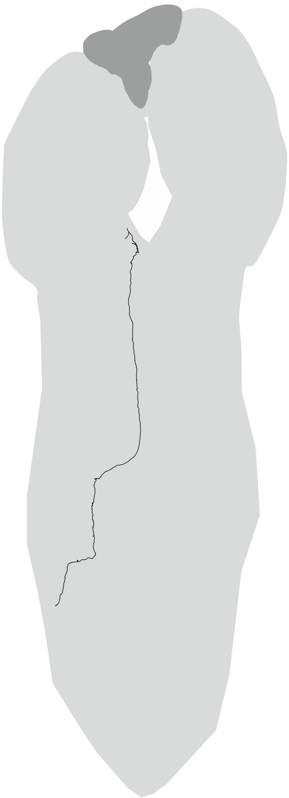

#9835169

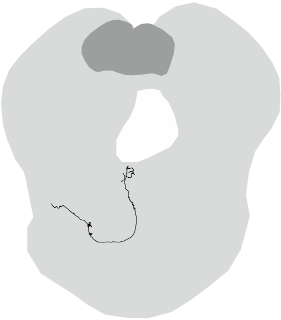

	synapses to	synapses from
hugin-PC right 1	0	0
hugin-PC right 2	0	0
hugin-PC right 3	0	0
hugin-PC right 4	0	0
hugin-PC left 1	0	0
hugin-PC left 2	0	0
hugin-PC left 3	0	0
hugin-PC left 4	1	0
hugin-RG right 1	0	0
hugin-RG right 2	0	0
hugin-RG left 1	0	0
hugin-RG left 2	0	0
hugin-VNC right 1	1	0
hugin-VNC right 2	2	0
hugin-VNC left 1	0	0
hugin-VNC left 2	0	0
hugin-PH right 1	0	0
hugin-PH right 2	0	0
hugin-PH left 1	0	0
hugin-PH left 2	0	0

4.

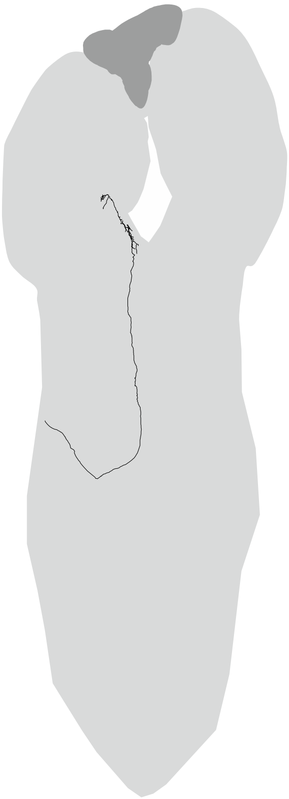

#11874130

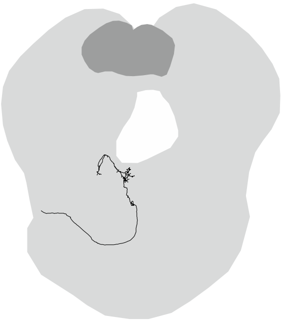

	synapses to	synapses from
hugin-PC right 1	0	0
hugin-PC right 2	0	0
hugin-PC right 3	0	0
hugin-PC right 4	0	0
hugin-PC left 1	0	0
hugin-PC left 2	0	0
hugin-PC left 3	1	0
hugin-PC left 4	0	0
hugin-RG right 1	0	0
hugin-RG right 2	0	0
hugin-RG left 1	0	0
hugin-RG left 2	0	0
hugin-VNC right 1	1	0
hugin-VNC right 2	3	0
hugin-VNC left 1	2	0
hugin-VNC left 2	0	0
hugin-PH right 1	0	0
hugin-PH right 2	0	0
hugin-PH left 1	0	0
hugin-PH left 2	0	0

5.

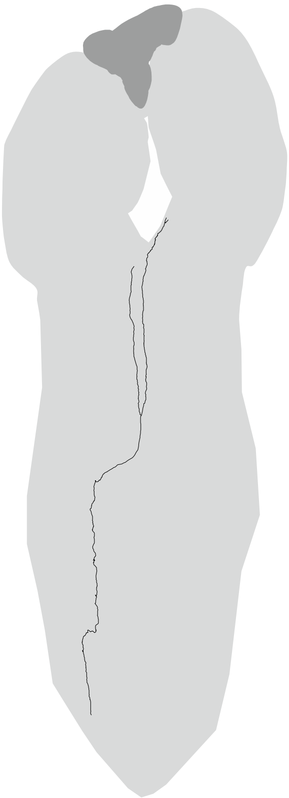

#7340629

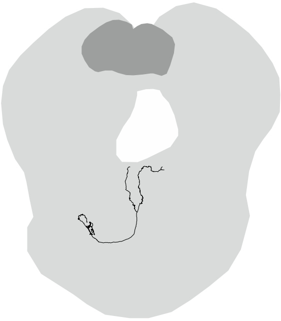

	synapses to	synapses from
hugin-PC right 1	0	0
hugin-PC right 2	0	0
hugin-PC right 3	0	0
hugin-PC right 4	0	0
hugin-PC left 1	0	0
hugin-PC left 2	0	0
hugin-PC left 3	0	0
hugin-PC left 4	0	0
hugin-RG right 1	0	0
hugin-RG right 2	0	0
hugin-RG left 1	0	0
hugin-RG left 2	0	0
hugin-VNC right 1	1	0
hugin-VNC right 2	0	0
hugin-VNC left 1	1	0
hugin-VNC left 2	2	0
hugin-PH right 1	0	0
hugin-PH right 2	0	0
hugin-PH left 1	0	0
hugin-PH left 2	0	0

6.

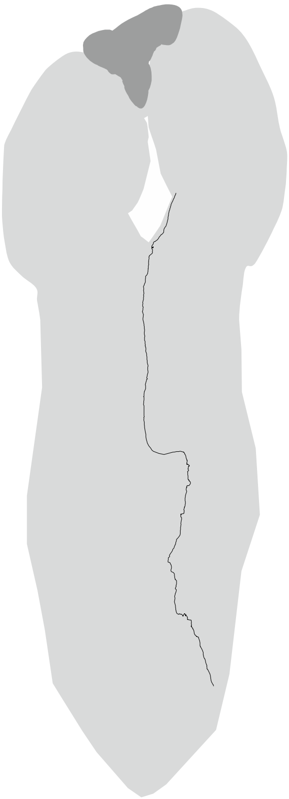

#7331555

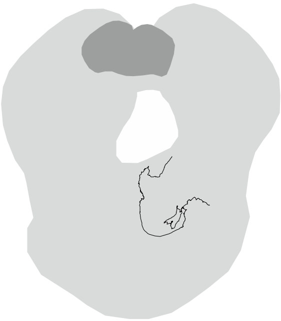

	synapses to	synapses from
hugin-PC right 1	0	0
hugin-PC right 2	0	0
hugin-PC right 3	0	0
hugin-PC right 4	0	0
hugin-PC left 1	0	0
hugin-PC left 2	0	0
hugin-PC left 3	0	0
hugin-PC left 4	0	0
hugin-RG right 1	0	0
hugin-RG right 2	0	0
hugin-RG left 1	0	0
hugin-RG left 2	0	0
hugin-VNC right 1	4	0
hugin-VNC right 2	0	0
hugin-VNC left 1	0	0
hugin-VNC left 2	4	0
hugin-PH right 1	0	0
hugin-PH right 2	0	0
hugin-PH left 1	0	0
hugin-PH left 2	0	0

7.

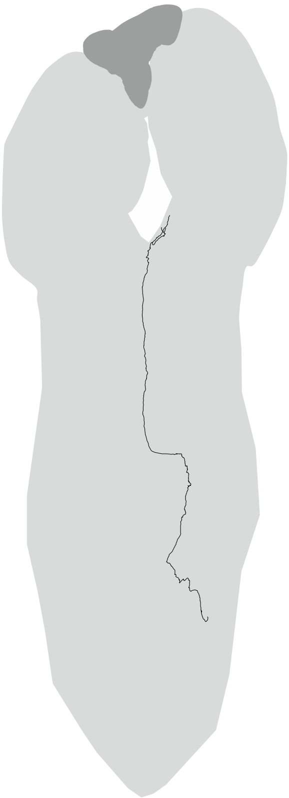

#11291344

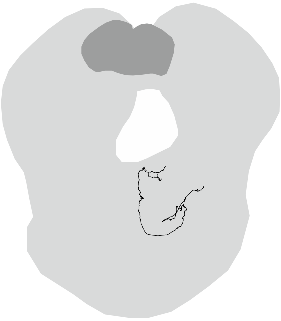

	synapses to	synapses from
hugin-PC right 1	0	0
hugin-PC right 2	0	0
hugin-PC right 3	0	0
hugin-PC right 4	0	0
hugin-PC left 1	0	0
hugin-PC left 2	0	0
hugin-PC left 3	0	0
hugin-PC left 4	0	0
hugin-RG right 1	0	0
hugin-RG right 2	0	0
hugin-RG left 1	0	0
hugin-RG left 2	0	0
hugin-VNC right 1	3	0
hugin-VNC right 2	0	0
hugin-VNC left 1	3	0
hugin-VNC left 2	4	0
hugin-PH right 1	0	0
hugin-PH right 2	0	0
hugin-PH left 1	0	0
hugin-PH left 2	0	0

8.

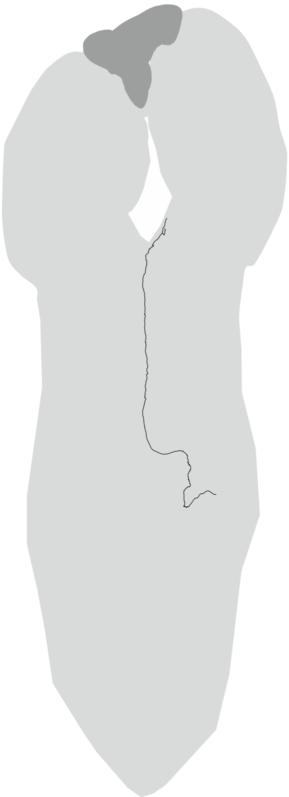

#5382876

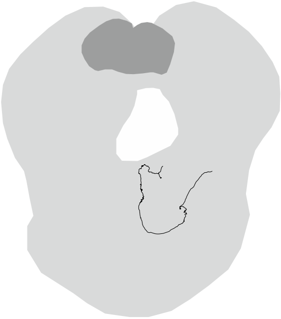

	synapses to	synapses from
hugin-PC right 1	0	0
hugin-PC right 2	0	0
hugin-PC right 3	0	0
hugin-PC right 4	0	0
hugin-PC left 1	0	0
hugin-PC left 2	0	0
hugin-PC left 3	0	0
hugin-PC left 4	0	0
hugin-RG right 1	0	0
hugin-RG right 2	0	0
hugin-RG left 1	0	0
hugin-RG left 2	0	0
hugin-VNC right 1	2	0
hugin-VNC right 2	0	0
hugin-VNC left 1	2	0
hugin-VNC left 2	3	0
hugin-PH right 1	0	0
hugin-PH right 2	0	0
hugin-PH left 1	0	0
hugin-PH left 2	0	0

9.

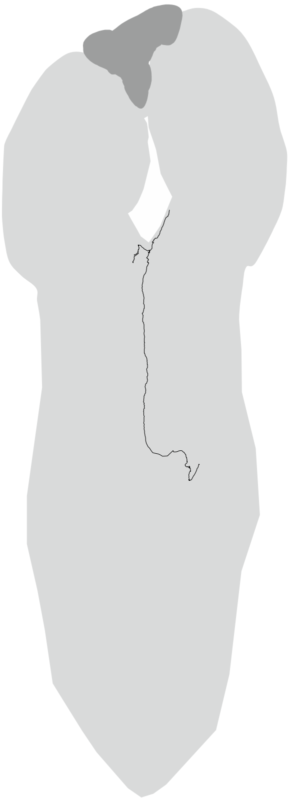

#10852875

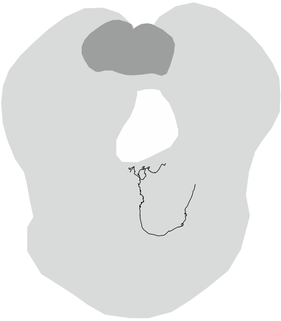

	synapses to	synapses from
hugin-PC right 1	0	0
hugin-PC right 2	0	0
hugin-PC right 3	0	0
hugin-PC right 4	0	0
hugin-PC left 1	0	0
hugin-PC left 2	0	0
hugin-PC left 3	0	0
hugin-PC left 4	0	0
hugin-RG right 1	0	0
hugin-RG right 2	1	0
hugin-RG left 1	0	0
hugin-RG left 2	0	0
hugin-VNC right 1	6	0
hugin-VNC right 2	0	0
hugin-VNC left 1	0	0
hugin-VNC left 2	0	0
hugin-PH right 1	0	0
hugin-PH right 2	0	0
hugin-PH left 1	0	0
hugin-PH left 2	0	0

10.

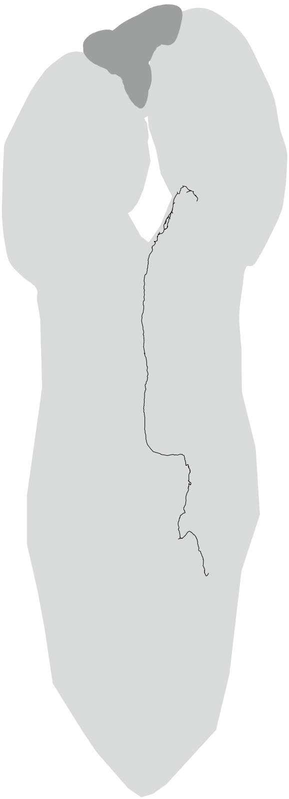

#9503203

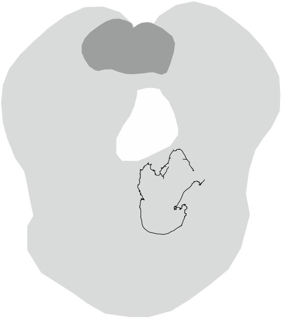

	synapses to	synapses from
hugin-PC right 1	0	0
hugin-PC right 2	1	0
hugin-PC right 3	0	0
hugin-PC right 4	0	0
hugin-PC left 1	0	0
hugin-PC left 2	0	0
hugin-PC left 3	0	0
hugin-PC left 4	0	0
hugin-RG right 1	0	0
hugin-RG right 2	0	0
hugin-RG left 1	0	0
hugin-RG left 2	0	0
hugin-VNC right 1	1	0
hugin-VNC right 2	0	0
hugin-VNC left 1	2	0
hugin-VNC left 2	0	0
hugin-PH right 1	0	0
hugin-PH right 2	0	0
hugin-PH left 1	0	0
hugin-PH left 2	0	0

11.

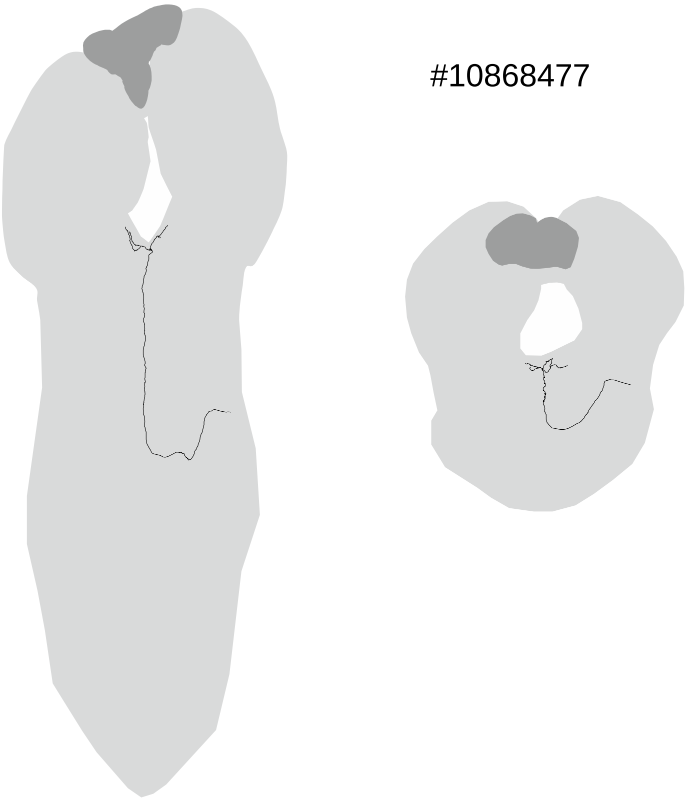

#10868477

	synapses to	synapses from
hugin-PC right 1	0	0
hugin-PC right 2	0	0
hugin-PC right 3	0	0
hugin-PC right 4	0	0
hugin-PC left 1	0	0
hugin-PC left 2	0	0
hugin-PC left 3	0	0
hugin-PC left 4	0	0
hugin-RG right 1	0	0
hugin-RG right 2	0	0
hugin-RG left 1	0	0
hugin-RG left 2	0	0
hugin-VNC right 1	2	0
hugin-VNC right 2	0	0
hugin-VNC left 1	1	0
hugin-VNC left 2	1	0
hugin-PH right 1	0	0
hugin-PH right 2	0	0
hugin-PH left 1	0	0
hugin-PH left 2	0	0

12.

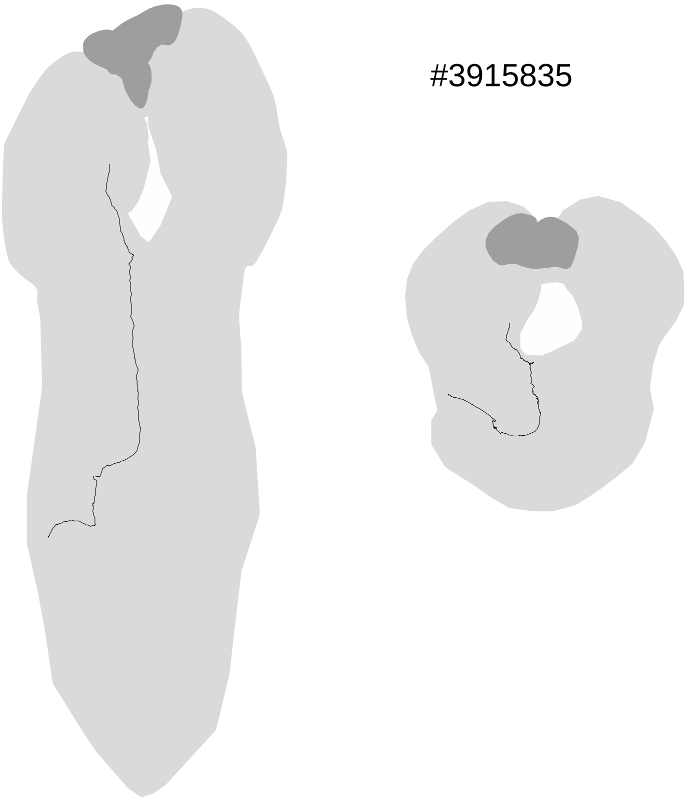

#3915835

	synapses to	synapses from
hugin-PC right 1	0	0
hugin-PC right 2	0	0
hugin-PC right 3	0	0
hugin-PC right 4	0	0
hugin-PC left 1	0	0
hugin-PC left 2	0	0
hugin-PC left 3	0	0
hugin-PC left 4	0	0
hugin-RG right 1	0	0
hugin-RG right 2	0	0
hugin-RG left 1	0	0
hugin-RG left 2	1	0
hugin-VNC right 1	0	0
hugin-VNC right 2	0	0
hugin-VNC left 1	0	0
hugin-VNC left 2	0	0
hugin-PH right 1	0	0
hugin-PH right 2	0	0
hugin-PH left 1	0	0
hugin-PH left 2	0	0

13.

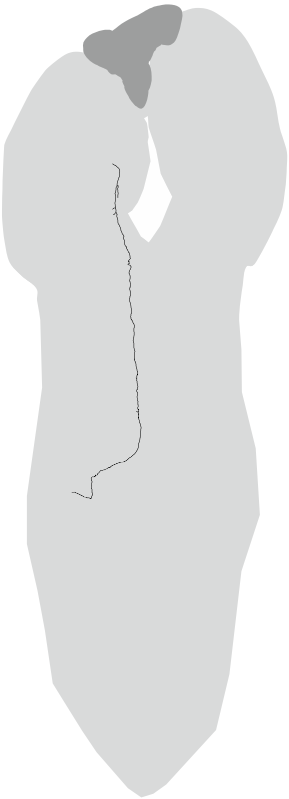

#5617164

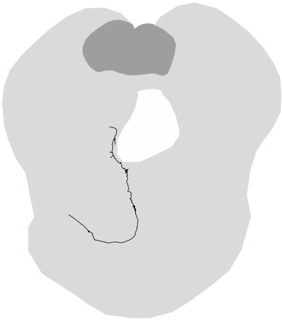

	synapses to	synapses from
hugin-PC right 1	0	0
hugin-PC right 2	0	0
hugin-PC right 3	0	0
hugin-PC right 4	0	0
hugin-PC left 1	0	0
hugin-PC left 2	0	0
hugin-PC left 3	0	0
hugin-PC left 4	0	0
hugin-RG right 1	0	0
hugin-RG right 2	0	0
hugin-RG left 1	0	0
hugin-RG left 2	1	0
hugin-VNC right 1	0	0
hugin-VNC right 2	0	0
hugin-VNC left 1	0	0
hugin-VNC left 2	0	0
hugin-PH right 1	0	0
hugin-PH right 2	0	0
hugin-PH left 1	0	0
hugin-PH left 2	0	0

14.

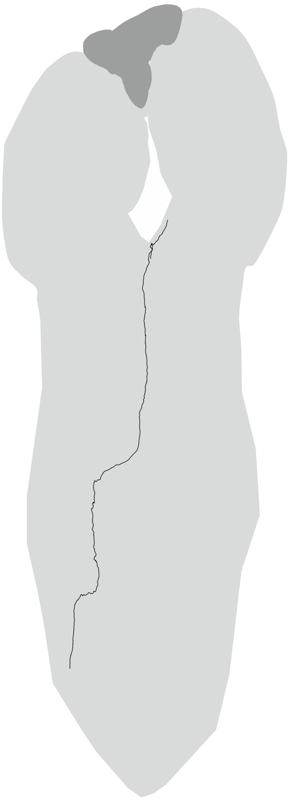

#3795424

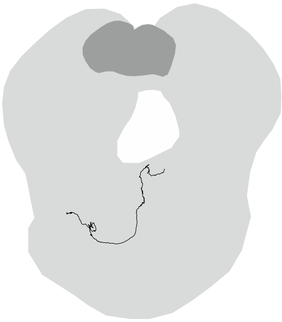

	synapses to	synapses from
hugin-PC right 1	0	0
hugin-PC right 2	0	0
hugin-PC right 3	0	0
hugin-PC right 4	0	0
hugin-PC left 1	0	0
hugin-PC left 2	0	0
hugin-PC left 3	0	0
hugin-PC left 4	0	0
hugin-RG right 1	0	0
hugin-RG right 2	0	0
hugin-RG left 1	0	0
hugin-RG left 2	0	0
hugin-VNC right 1	1	0
hugin-VNC right 2	0	0
hugin-VNC left 1	0	0
hugin-VNC left 2	0	0
hugin-PH right 1	0	0
hugin-PH right 2	0	0
hugin-PH left 1	0	0
hugin-PH left 2	0	0

J Interneurons

1.

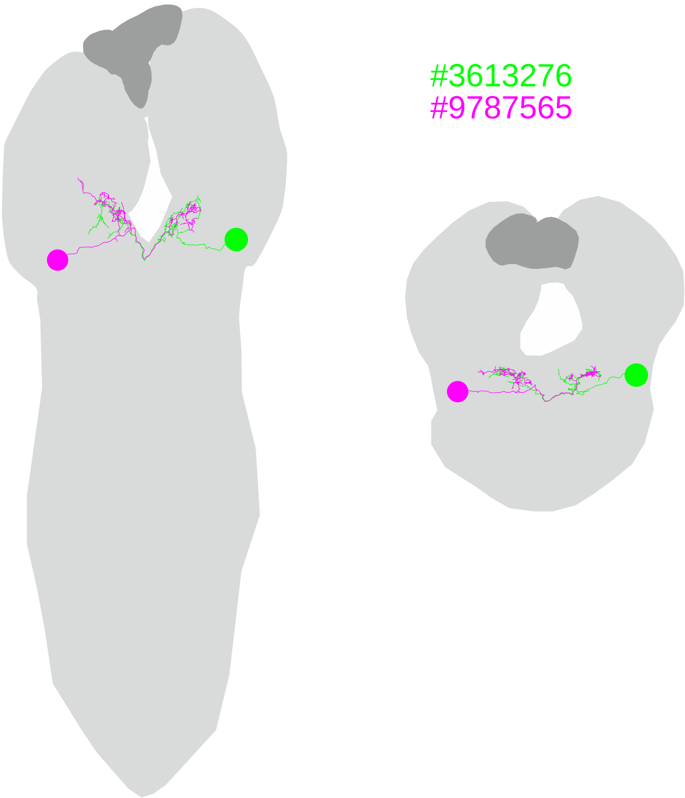

	synapses to		synapses from	
hugin-PC right 1	0	5	2	0
hugin-PC right 2	0	4	0	1
hugin-PC right 3	0	2	0	1
hugin-PC right 4	0	10	1	1
hugin-PC left 1	4	0	0	0
hugin-PC left 2	3	0	0	0
hugin-PC left 3	4	0	1	0
hugin-PC left 4	9	1	2	0
hugin-RG right 1	0	0	0	0
hugin-RG right 2	0	0	0	0
hugin-RG left 1	0	0	0	0
hugin-RG left 2	0	0	0	0
hugin-VNC right 1	0	0	0	0
hugin-VNC right 2	0	0	0	0
hugin-VNC left 1	0	0	0	0
hugin-VNC left 2	0	0	0	0
hugin-PH right 1	0	0	0	0
hugin-PH right 2	0	0	0	0
hugin-PH left 1	0	0	0	0
hugin-PH left 2	0	0	0	0

2.

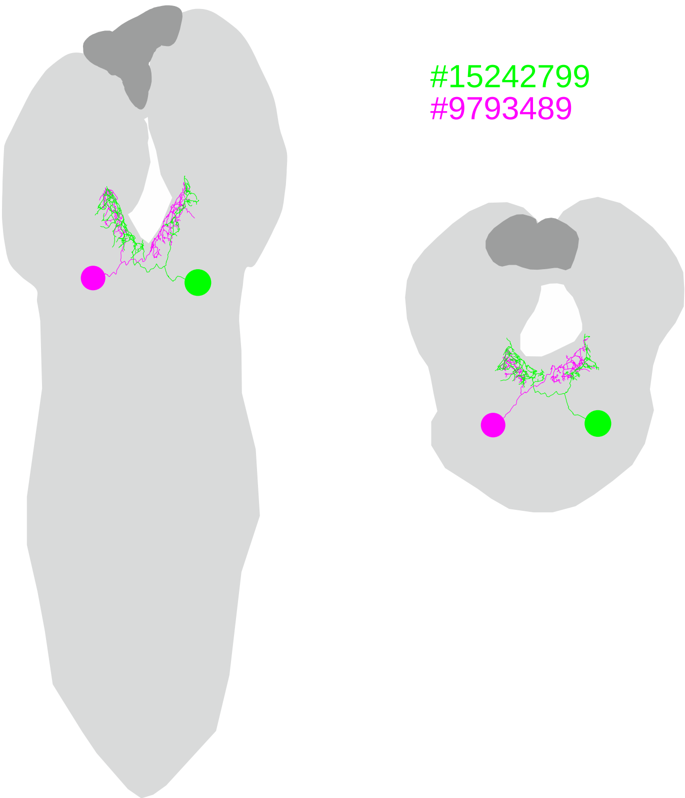

	synapses to		synapses from	
hugin-PC right 1	0	0	0	0
hugin-PC right 2	0	0	2	1
hugin-PC right 3	1	0	0	0
hugin-PC right 4	0	0	1	1
hugin-PC left 1	1	0	3	2
hugin-PC left 2	0	0	1	1
hugin-PC left 3	1	1	0	3
hugin-PC left 4	0	1	0	1
hugin-RG right 1	0	0	0	0
hugin-RG right 2	0	0	0	0
hugin-RG left 1	0	0	0	0
hugin-RG left 2	0	0	0	0
hugin-VNC right 1	0	0	0	0
hugin-VNC right 2	0	0	0	0
hugin-VNC left 1	0	0	0	0
hugin-VNC left 2	0	0	0	0
hugin-PH right 1	0	0	0	0
hugin-PH right 2	0	0	0	0
hugin-PH left 1	0	0	0	0
hugin-PH left 2	0	0	0	0

3.

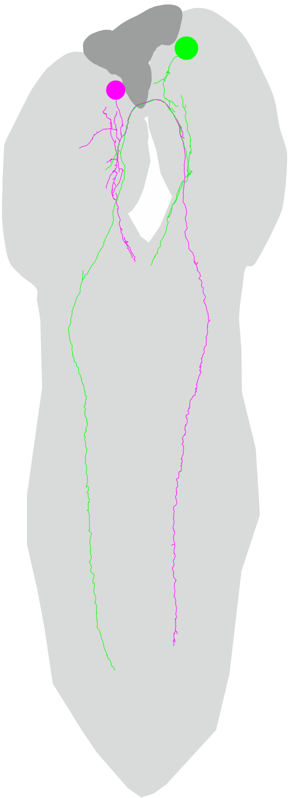

#3598000
#10716080

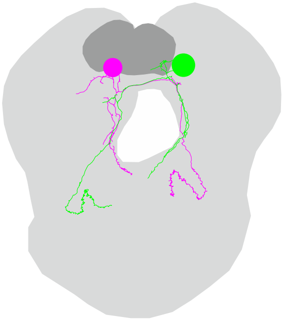

	synapses to		synapses from	
hugin-PC right 1	0	0	0	0
hugin-PC right 2	0	0	0	0
hugin-PC right 3	0	0	0	0
hugin-PC right 4	0	0	0	0
hugin-PC left 1	0	0	0	0
hugin-PC left 2	0	0	0	0
hugin-PC left 3	0	0	0	0
hugin-PC left 4	0	0	0	0
hugin-RG right 1	0	0	0	0
hugin-RG right 2	0	0	0	0
hugin-RG left 1	0	0	0	0
hugin-RG left 2	0	0	0	0
hugin-VNC right 1	4	0	0	0
hugin-VNC right 2	5	0	0	0
hugin-VNC left 1	0	5	0	0
hugin-VNC left 2	0	3	0	0
hugin-PH right 1	0	0	0	0
hugin-PH right 2	0	0	0	0
hugin-PH left 1	0	0	0	0
hugin-PH left 2	0	0	0	0

4.

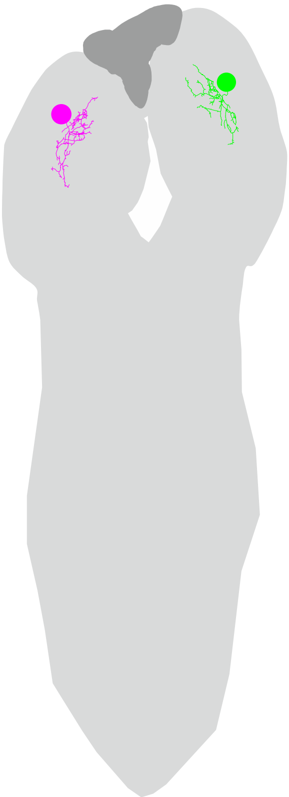

#2935198
#4785242

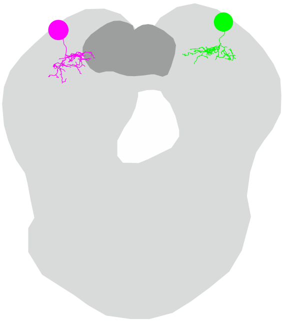

	synapses to		synapses from	
hugin-PC right 1	0	0	0	0
hugin-PC right 2	1	0	0	0
hugin-PC right 3	0	0	0	0
hugin-PC right 4	6	0	2	0
hugin-PC left 1	0	2	0	0
hugin-PC left 2	0	3	0	1
hugin-PC left 3	0	0	0	0
hugin-PC left 4	0	2	0	0
hugin-RG right 1	0	0	0	0
hugin-RG right 2	0	0	0	0
hugin-RG left 1	0	0	0	0
hugin-RG left 2	0	0	0	0
hugin-VNC right 1	0	0	0	0
hugin-VNC right 2	0	0	0	0
hugin-VNC left 1	0	0	0	0
hugin-VNC left 2	0	0	0	0
hugin-PH right 1	0	0	0	0
hugin-PH right 2	0	0	0	0
hugin-PH left 1	0	0	0	0
hugin-PH left 2	0	0	0	0

5.

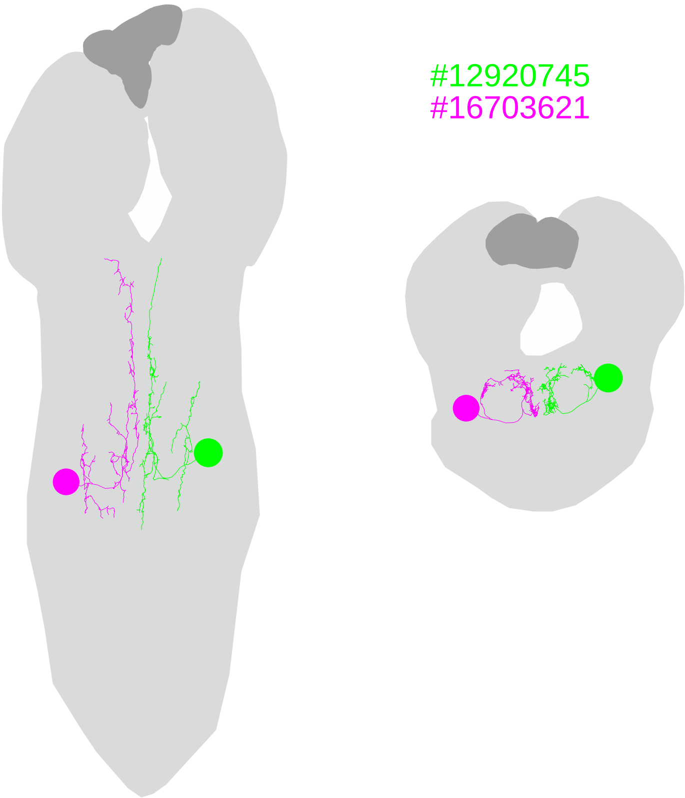

	synapses to		synapses from	
hugin-PC right 1	0	0	0	0
hugin-PC right 2	0	0	0	0
hugin-PC right 3	0	0	0	0
hugin-PC right 4	0	0	0	0
hugin-PC left 1	0	0	0	0
hugin-PC left 2	0	0	0	0
hugin-PC left 3	0	0	0	0
hugin-PC left 4	0	0	0	0
hugin-RG right 1	0	0	0	0
hugin-RG right 2	0	0	0	0
hugin-RG left 1	0	0	0	0
hugin-RG left 2	0	0	0	0
hugin-VNC right 1	0	0	0	5
hugin-VNC right 2	0	0	0	3
hugin-VNC left 1	0	0	5	0
hugin-VNC left 2	0	0	2	0
hugin-PH right 1	0	0	0	0
hugin-PH right 2	0	0	0	0
hugin-PH left 1	0	0	0	0
hugin-PH left 2	0	0	0	0

6.

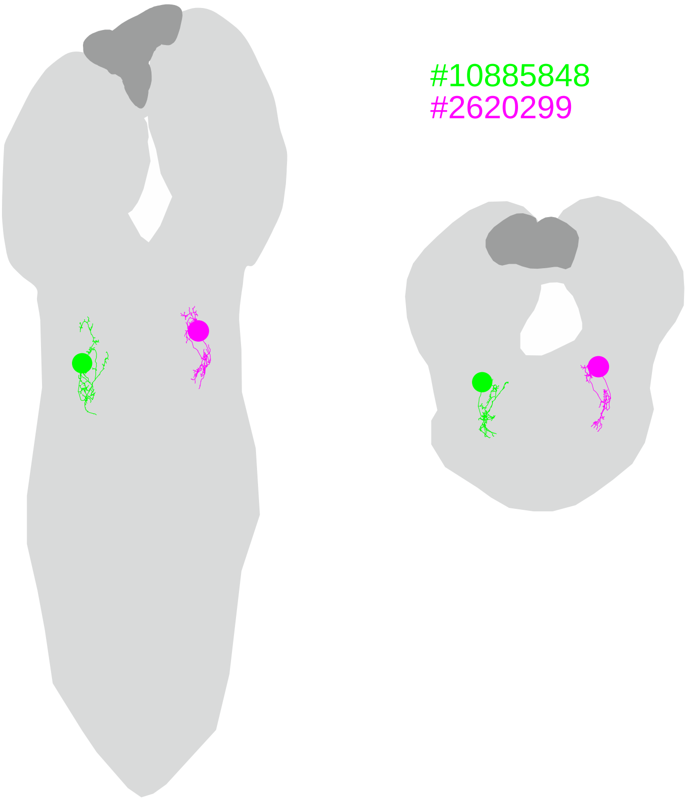

	synapses to		synapses from	
hugin-PC right 1	0	0	0	0
hugin-PC right 2	0	0	0	0
hugin-PC right 3	0	0	0	0
hugin-PC right 4	0	0	0	0
hugin-PC left 1	0	0	0	0
hugin-PC left 2	0	0	0	0
hugin-PC left 3	0	0	0	0
hugin-PC left 4	0	0	0	0
hugin-RG right 1	0	0	0	0
hugin-RG right 2	0	0	0	0
hugin-RG left 1	0	0	0	0
hugin-RG left 2	0	0	0	0
hugin-VNC right 1	0	0	4	0
hugin-VNC right 2	0	0	2	0
hugin-VNC left 1	0	0	0	4
hugin-VNC left 2	0	0	0	3
hugin-PH right 1	0	0	0	0
hugin-PH right 2	0	0	0	0
hugin-PH left 1	0	0	0	0
hugin-PH left 2	0	0	0	0

7.

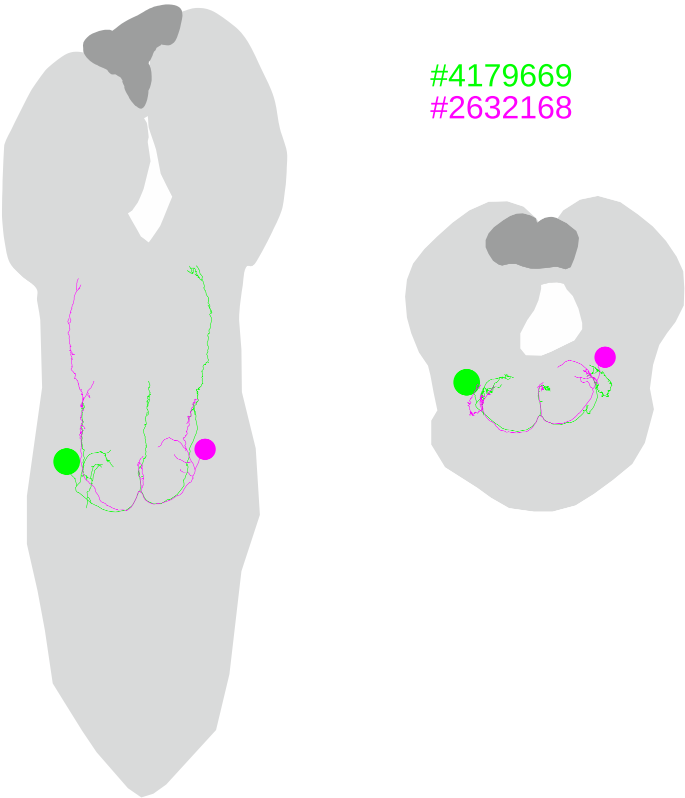

	synapses to		synapses from	
hugin-PC right 1	0	0	0	0
hugin-PC right 2	0	0	0	0
hugin-PC right 3	0	0	0	0
hugin-PC right 4	0	0	0	0
hugin-PC left 1	0	0	0	0
hugin-PC left 2	0	0	0	0
hugin-PC left 3	0	0	0	0
hugin-PC left 4	0	0	0	0
hugin-RG right 1	0	0	0	0
hugin-RG right 2	0	0	0	0
hugin-RG left 1	0	0	0	0
hugin-RG left 2	0	0	0	0
hugin-VNC right 1	0	1	2	1
hugin-VNC right 2	0	1	1	0
hugin-VNC left 1	0	0	1	2
hugin-VNC left 2	4	0	1	2
hugin-PH right 1	0	0	0	0
hugin-PH right 2	0	0	0	0
hugin-PH left 1	0	0	0	0
hugin-PH left 2	0	0	0	0

8.

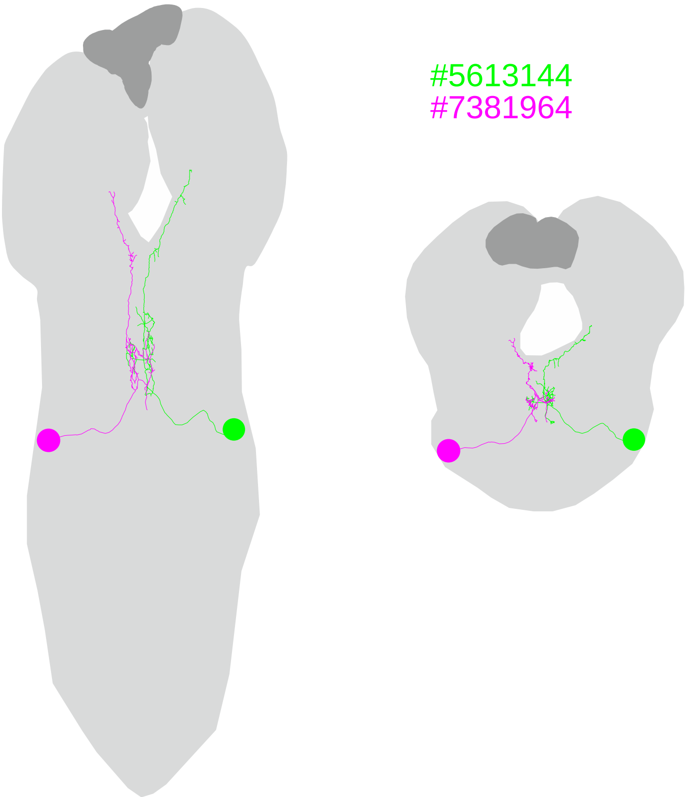

	synapses to		synapses from	
hugin-PC right 1	0	0	0	0
hugin-PC right 2	0	0	0	0
hugin-PC right 3	0	0	0	0
hugin-PC right 4	0	0	0	0
hugin-PC left 1	0	0	0	0
hugin-PC left 2	0	0	0	0
hugin-PC left 3	0	1	0	0
hugin-PC left 4	0	0	0	0
hugin-RG right 1	12	4	0	0
hugin-RG right 2	15	6	0	0
hugin-RG left 1	6	9	0	0
hugin-RG left 2	6	14	0	0
hugin-VNC right 1	0	0	0	0
hugin-VNC right 2	0	1	0	0
hugin-VNC left 1	0	0	0	0
hugin-VNC left 2	0	0	0	0
hugin-PH right 1	0	1	0	0
hugin-PH right 2	0	1	0	0
hugin-PH left 1	0	0	0	0
hugin-PH left 2	0	0	0	0

9.

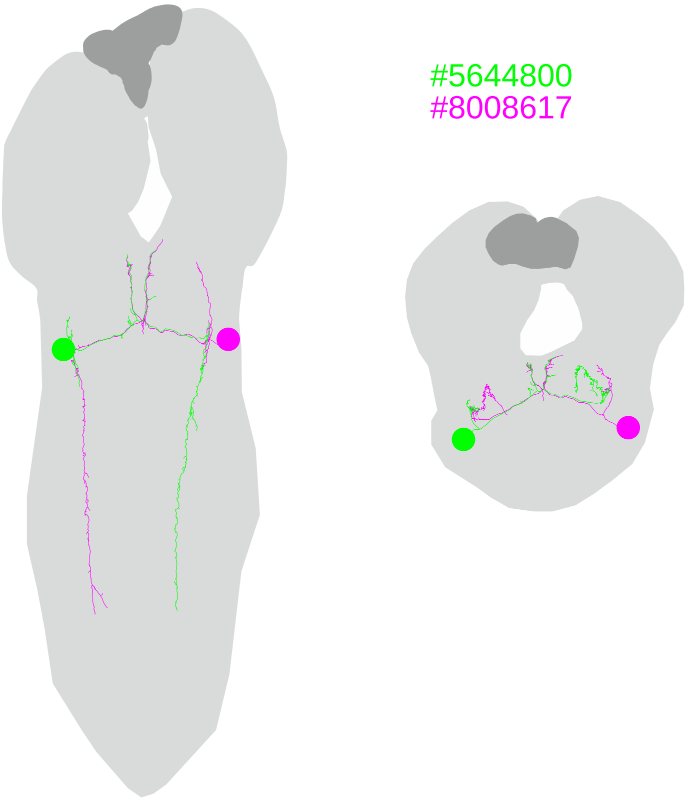

	synapses to		synapses from	
hugin-PC right 1	0	0	0	0
hugin-PC right 2	0	0	0	0
hugin-PC right 3	0	0	0	0
hugin-PC right 4	0	0	0	0
hugin-PC left 1	0	0	0	0
hugin-PC left 2	0	0	0	0
hugin-PC left 3	0	0	0	0
hugin-PC left 4	0	0	0	0
hugin-RG right 1	0	0	0	0
hugin-RG right 2	0	0	0	0
hugin-RG left 1	0	0	0	0
hugin-RG left 2	0	0	0	0
hugin-VNC right 1	0	1	4	5
hugin-VNC right 2	0	0	2	3
hugin-VNC left 1	1	0	7	4
hugin-VNC left 2	1	0	5	6
hugin-PH right 1	0	0	0	0
hugin-PH right 2	0	0	0	0
hugin-PH left 1	0	0	0	0
hugin-PH left 2	0	0	0	0

10.

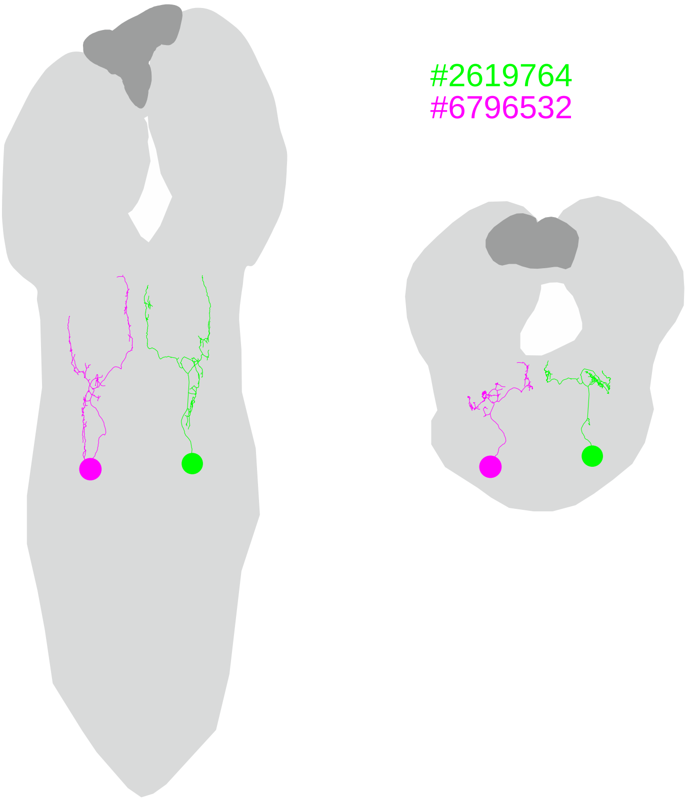

	synapses to		synapses from	
hugin-PC right 1	0	0	0	0
hugin-PC right 2	0	0	0	0
hugin-PC right 3	0	0	0	0
hugin-PC right 4	0	0	0	0
hugin-PC left 1	0	0	0	0
hugin-PC left 2	0	0	0	0
hugin-PC left 3	0	0	0	0
hugin-PC left 4	0	0	0	0
hugin-RG right 1	0	0	0	0
hugin-RG right 2	0	0	0	0
hugin-RG left 1	0	0	0	0
hugin-RG left 2	0	0	0	0
hugin-VNC right 1	0	0	0	7
hugin-VNC right 2	0	0	0	4
hugin-VNC left 1	0	0	1	0
hugin-VNC left 2	0	0	3	0
hugin-PH right 1	0	0	0	0
hugin-PH right 2	0	0	0	0
hugin-PH left 1	0	0	0	0
hugin-PH left 2	0	0	0	0

11.

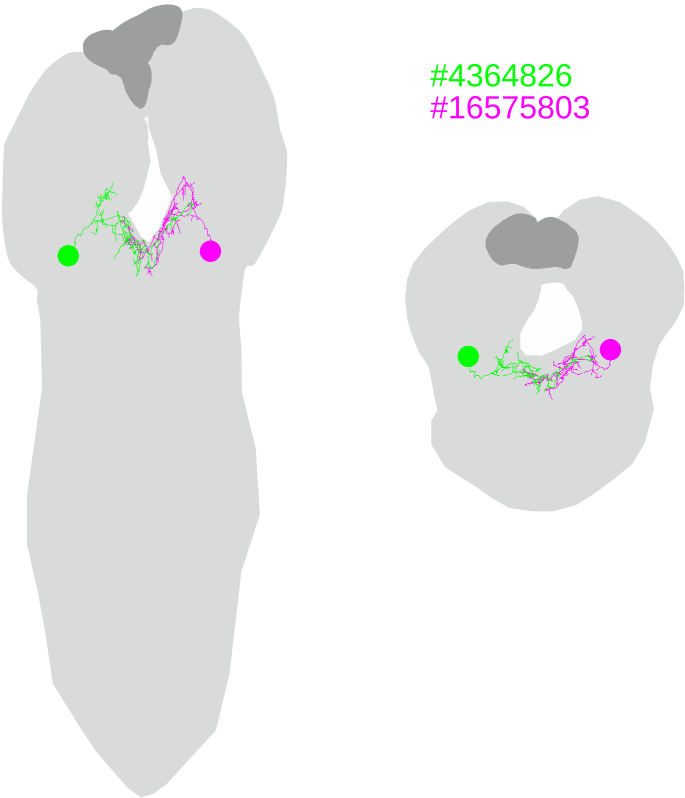

	synapses to		synapses from	
hugin-PC right 1	0	3	0	0
hugin-PC right 2	0	0	0	0
hugin-PC right 3	4	0	0	0
hugin-PC right 4	2	3	1	3
hugin-PC left 1	2	2	3	1
hugin-PC left 2	2	4	0	0
hugin-PC left 3	0	0	0	2
hugin-PC left 4	0	2	0	0
hugin-RG right 1	0	0	0	0
hugin-RG right 2	0	0	0	0
hugin-RG left 1	0	0	0	0
hugin-RG left 2	0	0	0	0
hugin-VNC right 1	0	0	0	0
hugin-VNC right 2	0	0	0	0
hugin-VNC left 1	0	0	0	0
hugin-VNC left 2	0	0	0	0
hugin-PH right 1	0	0	0	0
hugin-PH right 2	0	0	0	0
hugin-PH left 1	0	0	0	0
hugin-PH left 2	0	0	0	0

12.

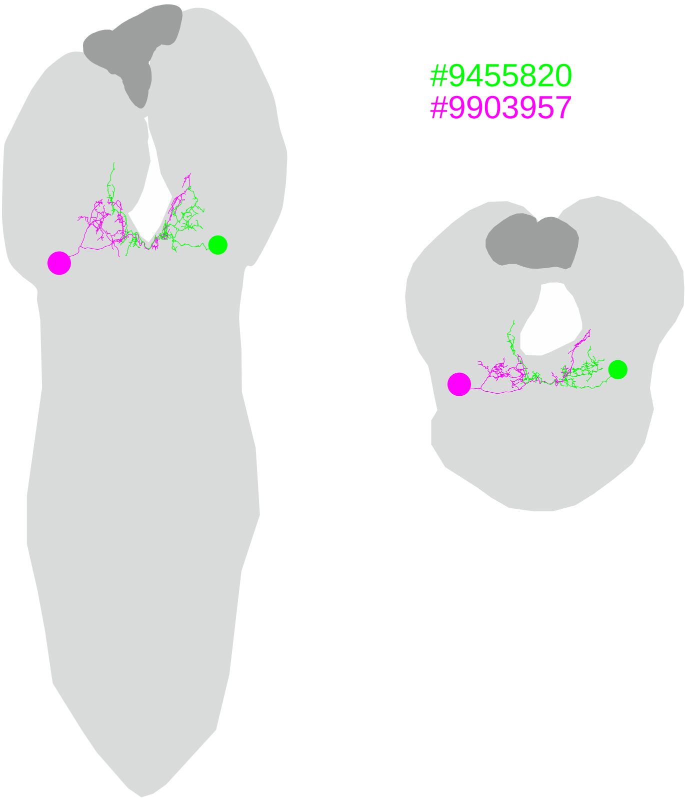

	synapses to		synapses from	
hugin-PC right 1	0	2	2	0
hugin-PC right 2	0	1	0	0
hugin-PC right 3	0	2	2	0
hugin-PC right 4	0	1	2	0
hugin-PC left 1	4	0	1	0
hugin-PC left 2	2	0	1	1
hugin-PC left 3	5	0	2	1
hugin-PC left 4	5	1	0	1
hugin-RG right 1	0	0	0	0
hugin-RG right 2	0	0	0	0
hugin-RG left 1	0	0	0	0
hugin-RG left 2	0	0	0	0
hugin-VNC right 1	0	0	0	0
hugin-VNC right 2	0	0	0	0
hugin-VNC left 1	0	0	0	0
hugin-VNC left 2	0	0	0	0
hugin-PH right 1	0	0	0	0
hugin-PH right 2	0	0	0	0
hugin-PH left 1	0	0	0	0
hugin-PH left 2	0	0	0	0

13.

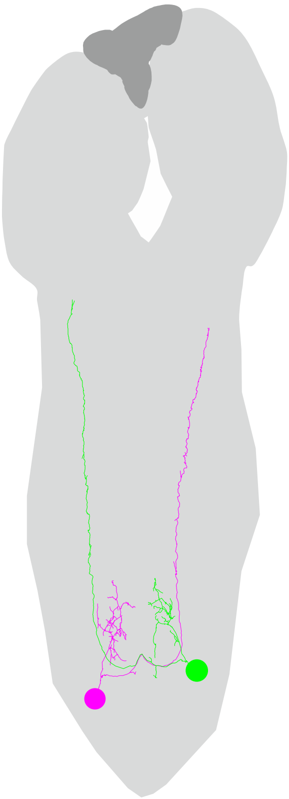

#9563254
#11517136

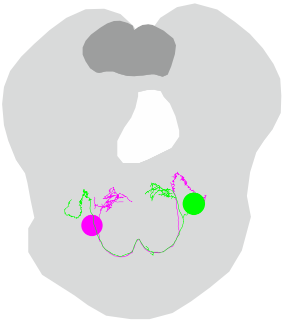

	synapses to		synapses from	
hugin-PC right 1	0	0	0	0
hugin-PC right 2	0	0	0	0
hugin-PC right 3	0	0	0	0
hugin-PC right 4	0	0	0	0
hugin-PC left 1	0	0	0	0
hugin-PC left 2	0	0	0	0
hugin-PC left 3	0	0	0	0
hugin-PC left 4	0	0	0	0
hugin-RG right 1	0	0	0	0
hugin-RG right 2	0	0	0	0
hugin-RG left 1	0	0	0	0
hugin-RG left 2	0	0	0	0
hugin-VNC right 1	1	0	3	0
hugin-VNC right 2	4	0	0	0
hugin-VNC left 1	0	6	0	1
hugin-VNC left 2	0	1	0	1
hugin-PH right 1	0	0	0	0
hugin-PH right 2	0	0	0	0
hugin-PH left 1	0	0	0	0
hugin-PH left 2	0	0	0	0

14.

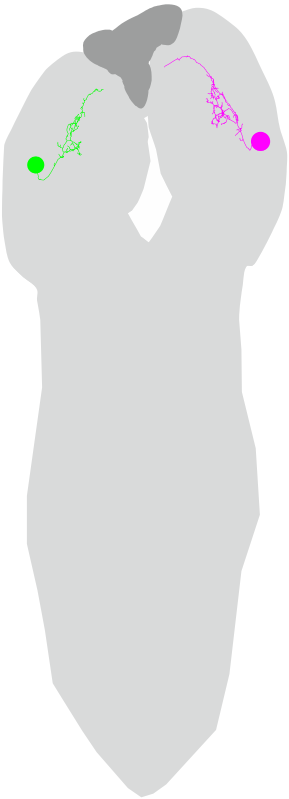

#3946432
#2935173

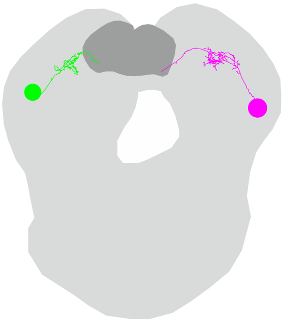

	synapses to		synapses from	
hugin-PC right 1	0	4	0	0
hugin-PC right 2	0	2	0	0
hugin-PC right 3	0	0	0	0
hugin-PC right 4	0	3	0	1
hugin-PC left 1	0	0	0	0
hugin-PC left 2	5	0	0	0
hugin-PC left 3	0	0	0	0
hugin-PC left 4	0	0	0	0
hugin-RG right 1	0	0	0	0
hugin-RG right 2	0	0	0	0
hugin-RG left 1	0	0	0	0
hugin-RG left 2	0	0	0	0
hugin-VNC right 1	0	0	0	0
hugin-VNC right 2	0	0	0	0
hugin-VNC left 1	0	0	0	0
hugin-VNC left 2	0	0	0	0
hugin-PH right 1	0	0	0	0
hugin-PH right 2	0	0	0	0
hugin-PH left 1	0	0	0	0
hugin-PH left 2	0	0	0	0

15.

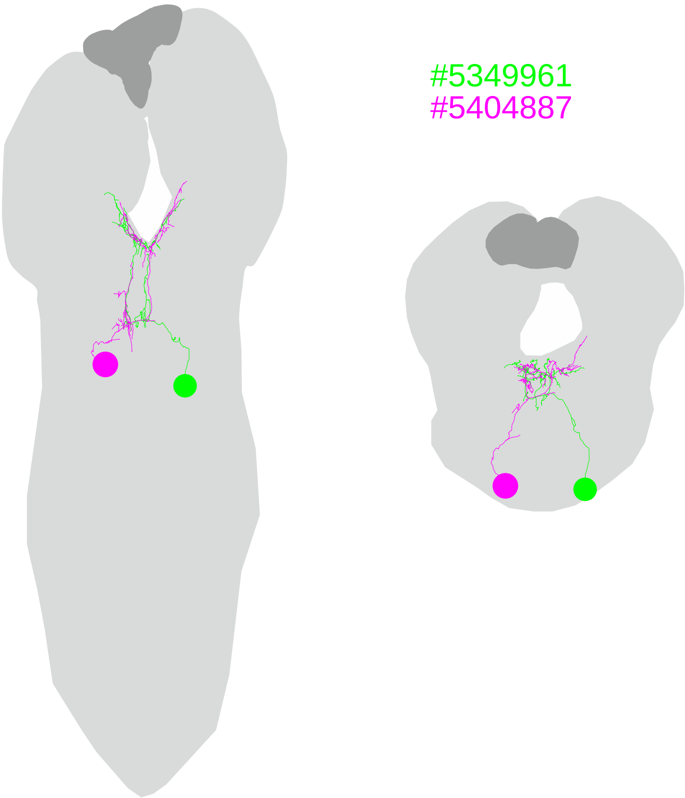

	synapses to		synapses from	
hugin-PC right 1	0	0	0	0
hugin-PC right 2	0	0	0	0
hugin-PC right 3	0	0	0	0
hugin-PC right 4	0	1	0	0
hugin-PC left 1	0	0	1	0
hugin-PC left 2	0	1	0	1
hugin-PC left 3	0	0	0	0
hugin-PC left 4	0	0	0	0
hugin-RG right 1	0	0	0	0
hugin-RG right 2	0	0	0	0
hugin-RG left 1	0	0	0	0
hugin-RG left 2	0	0	0	0
hugin-VNC right 1	1	5	0	0
hugin-VNC right 2	3	2	0	0
hugin-VNC left 1	3	4	0	0
hugin-VNC left 2	1	0	0	0
hugin-PH right 1	0	0	0	0
hugin-PH right 2	0	0	0	0
hugin-PH left 1	0	0	0	0
hugin-PH left 2	0	0	0	0

16.

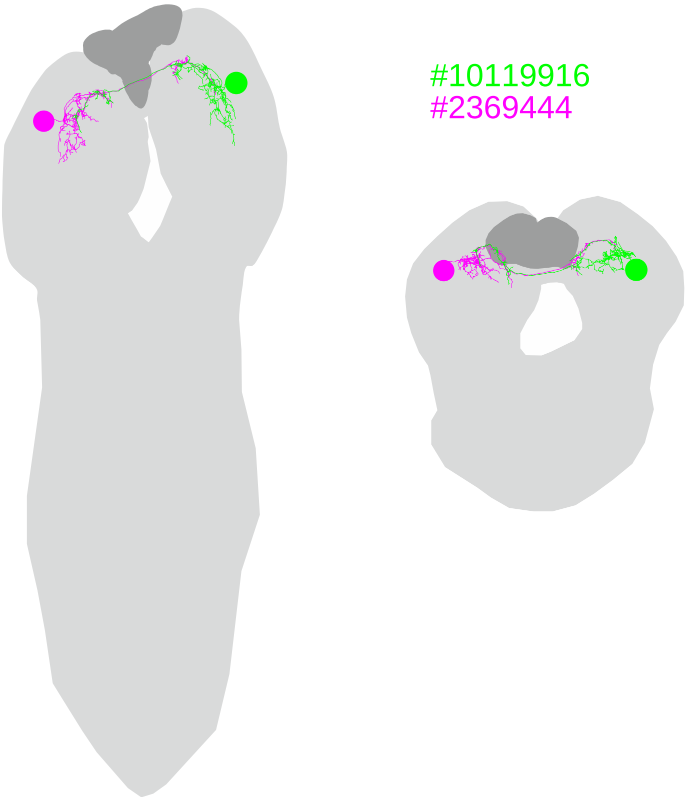

	synapses to		synapses from	
hugin-PC right 1	9	12	1	0
hugin-PC right 2	4	6	0	1
hugin-PC right 3	6	4	0	0
hugin-PC right 4	13	7	2	3
hugin-PC left 1	12	4	1	3
hugin-PC left 2	7	10	1	2
hugin-PC left 3	3	0	0	2
hugin-PC left 4	7	8	2	1
hugin-RG right 1	0	0	0	0
hugin-RG right 2	0	0	0	0
hugin-RG left 1	0	0	0	0
hugin-RG left 2	0	0	0	0
hugin-VNC right 1	0	0	0	0
hugin-VNC right 2	0	0	0	0
hugin-VNC left 1	0	0	0	0
hugin-VNC left 2	0	0	0	0
hugin-PH right 1	0	0	0	0
hugin-PH right 2	0	0	0	0
hugin-PH left 1	0	0	0	0
hugin-PH left 2	0	0	0	0

17.

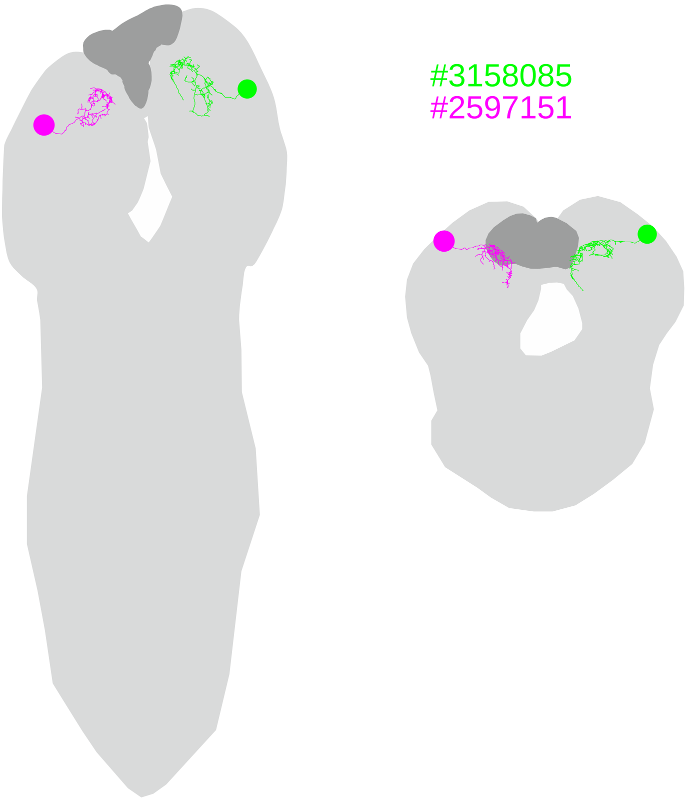

	synapses to		synapses from	
hugin-PC right 1	5	0	1	0
hugin-PC right 2	2	0	0	0
hugin-PC right 3	2	0	0	0
hugin-PC right 4	1	0	1	0
hugin-PC left 1	0	9	0	0
hugin-PC left 2	0	7	0	3
hugin-PC left 3	0	3	0	0
hugin-PC left 4	0	5	0	0
hugin-RG right 1	0	0	0	0
hugin-RG right 2	0	0	0	0
hugin-RG left 1	0	0	0	0
hugin-RG left 2	0	0	0	0
hugin-VNC right 1	0	0	0	0
hugin-VNC right 2	0	0	0	0
hugin-VNC left 1	0	0	0	0
hugin-VNC left 2	0	0	0	0
hugin-PH right 1	0	0	0	0
hugin-PH right 2	0	0	0	0
hugin-PH left 1	0	0	0	0
hugin-PH left 2	0	0	0	0

18.

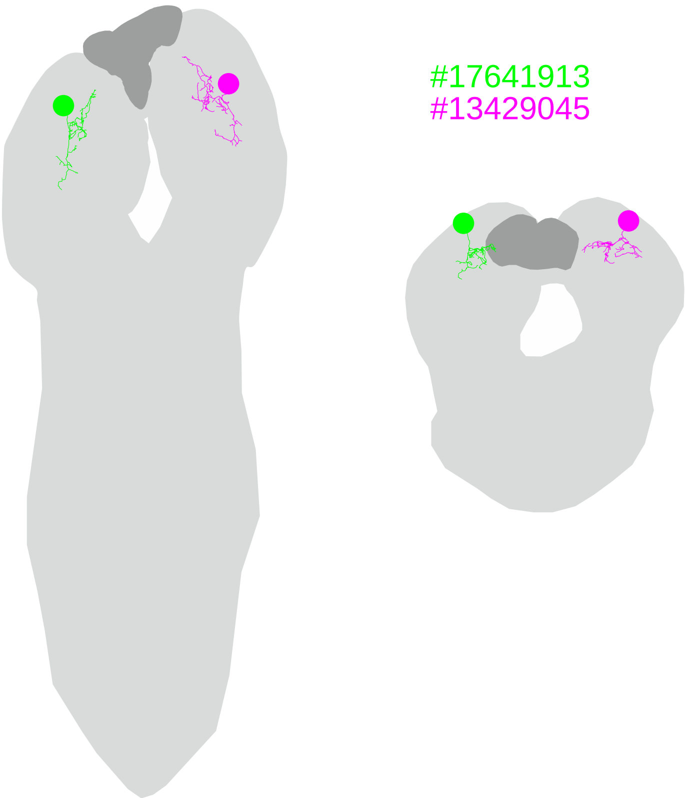

	synapses to		synapses from	
hugin-PC right 1	0	1	0	0
hugin-PC right 2	0	2	0	0
hugin-PC right 3	0	0	0	0
hugin-PC right 4	0	2	0	2
hugin-PC left 1	2	0	1	0
hugin-PC left 2	4	0	2	0
hugin-PC left 3	0	0	0	0
hugin-PC left 4	3	0	4	0
hugin-RG right 1	0	0	0	0
hugin-RG right 2	0	0	0	0
hugin-RG left 1	0	0	0	0
hugin-RG left 2	0	0	0	0
hugin-VNC right 1	0	0	0	0
hugin-VNC right 2	0	0	0	0
hugin-VNC left 1	0	0	0	0
hugin-VNC left 2	0	0	0	0
hugin-PH right 1	0	0	0	0
hugin-PH right 2	0	0	0	0
hugin-PH left 1	0	0	0	0
hugin-PH left 2	0	0	0	0

19.

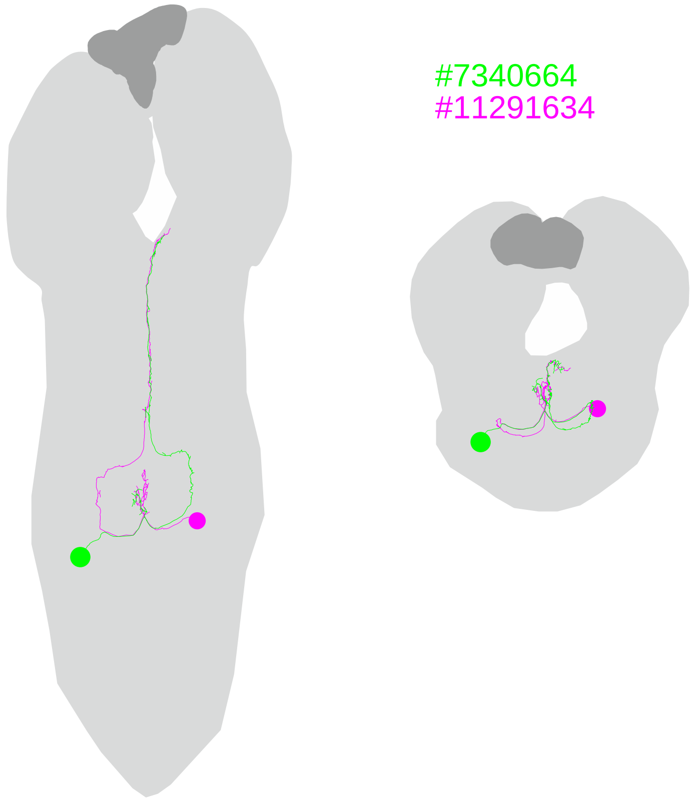

	synapses to (green)	synapses to (magenta)	synapses from (green)	synapses from (magenta)
hugin-PC right 1	0	0	0	0
hugin-PC right 2	0	0	0	0
hugin-PC right 3	0	0	0	0
hugin-PC right 4	0	0	0	0
hugin-PC left 1	0	0	0	0
hugin-PC left 2	0	0	0	0
hugin-PC left 3	0	0	0	0
hugin-PC left 4	0	0	0	0
hugin-RG right 1	1	0	0	0
hugin-RG right 2	0	0	0	0
hugin-RG left 1	0	0	0	0
hugin-RG left 2	0	0	0	0
hugin-VNC right 1	2	4	0	0
hugin-VNC right 2	0	0	0	0
hugin-VNC left 1	3	2	0	0
hugin-VNC left 2	1	2	0	0
hugin-PH right 1	0	0	0	0
hugin-PH right 2	0	0	0	0
hugin-PH left 1	0	0	0	0
hugin-PH left 2	0	0	0	0

20.

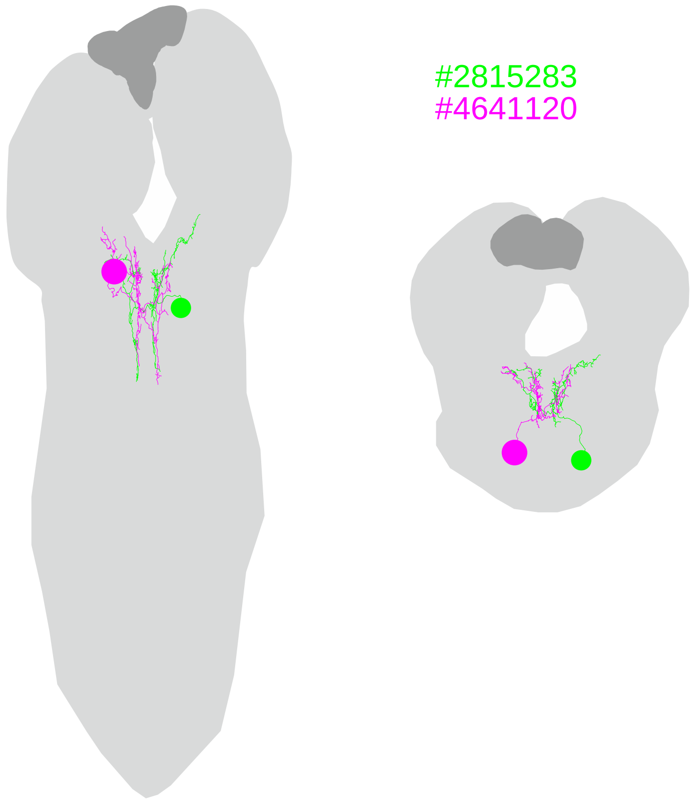

	synapses to (green)	synapses to (magenta)	synapses from (green)	synapses from (magenta)
hugin-PC right 1	0	0	0	0
hugin-PC right 2	0	0	0	0
hugin-PC right 3	0	0	0	0
hugin-PC right 4	0	0	0	0
hugin-PC left 1	0	0	0	0
hugin-PC left 2	0	0	0	0
hugin-PC left 3	0	0	0	0
hugin-PC left 4	0	0	0	0
hugin-RG right 1	0	0	0	0
hugin-RG right 2	0	0	0	0
hugin-RG left 1	0	0	0	0
hugin-RG left 2	0	0	0	0
hugin-VNC right 1	0	0	0	0
hugin-VNC right 2	0	0	0	0
hugin-VNC left 1	0	0	0	0
hugin-VNC left 2	0	0	0	0
hugin-PH right 1	10	0	0	0
hugin-PH right 2	7	4	0	0
hugin-PH left 1	3	3	0	0
hugin-PH left 2	3	4	0	0

21.

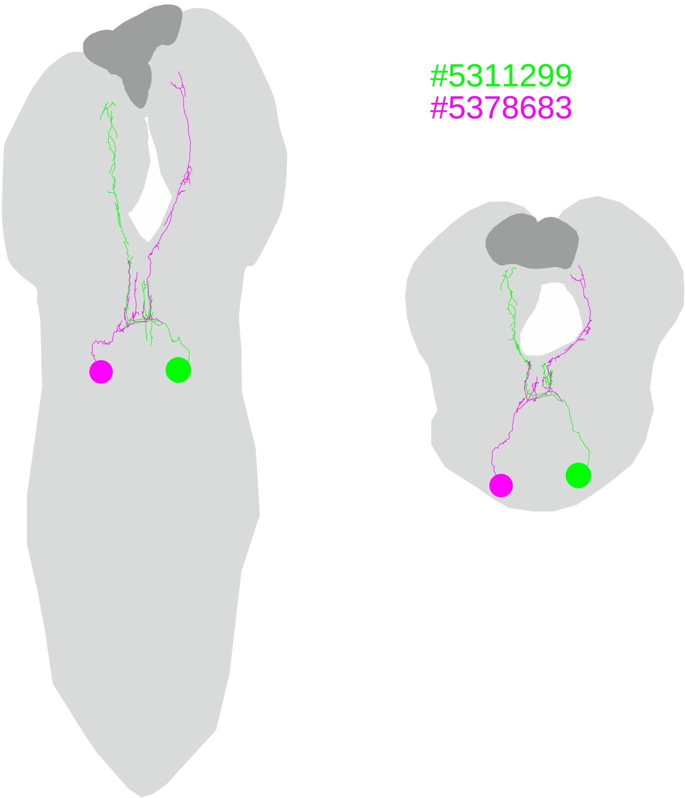

	synapses to		synapses from	
hugin-PC right 1	0	0	0	0
hugin-PC right 2	0	0	0	0
hugin-PC right 3	0	0	0	0
hugin-PC right 4	0	0	0	0
hugin-PC left 1	0	0	0	0
hugin-PC left 2	0	0	0	0
hugin-PC left 3	0	0	0	0
hugin-PC left 4	0	0	0	0
hugin-RG right 1	0	0	0	0
hugin-RG right 2	0	0	0	0
hugin-RG left 1	0	0	0	0
hugin-RG left 2	0	0	0	0
hugin-VNC right 1	0	1	0	0
hugin-VNC right 2	4	0	0	0
hugin-VNC left 1	4	0	0	0
hugin-VNC left 2	0	0	0	0
hugin-PH right 1	0	0	0	0
hugin-PH right 2	0	0	0	0
hugin-PH left 1	0	0	0	0
hugin-PH left 2	0	0	0	0

22.

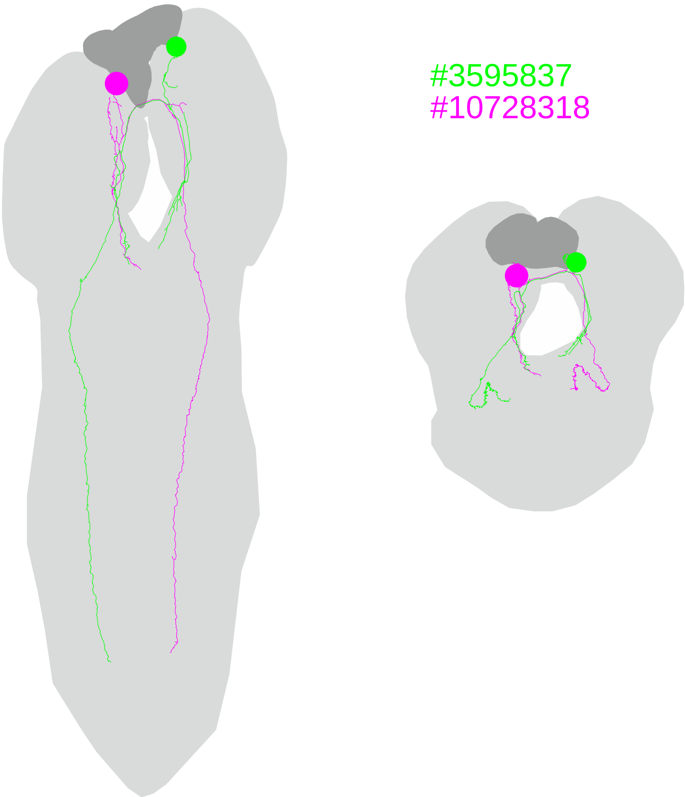

	synapses to		synapses from	
hugin-PC right 1	0	0	0	0
hugin-PC right 2	0	0	0	0
hugin-PC right 3	0	0	0	0
hugin-PC right 4	0	0	0	0
hugin-PC left 1	0	0	0	0
hugin-PC left 2	0	0	0	0
hugin-PC left 3	0	0	0	0
hugin-PC left 4	0	0	0	0
hugin-RG right 1	0	0	0	0
hugin-RG right 2	0	0	0	0
hugin-RG left 1	0	0	0	0
hugin-RG left 2	0	0	0	0
hugin-VNC right 1	4	0	0	0
hugin-VNC right 2	7	0	1	0
hugin-VNC left 1	0	5	0	0
hugin-VNC left 2	0	3	0	1
hugin-PH right 1	0	0	0	0
hugin-PH right 2	0	0	0	0
hugin-PH left 1	0	0	0	0
hugin-PH left 2	0	0	0	0

23.

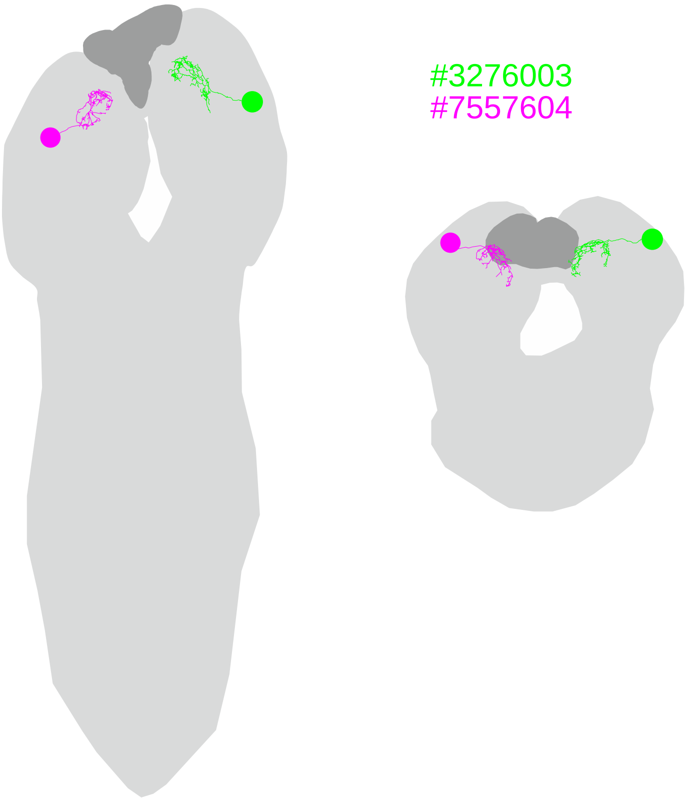

	synapses to		synapses from	
hugin-PC right 1	4	0	2	0
hugin-PC right 2	3	0	0	0
hugin-PC right 3	3	0	0	0
hugin-PC right 4	5	0	0	0
hugin-PC left 1	0	4	0	0
hugin-PC left 2	0	1	0	0
hugin-PC left 3	0	1	0	0
hugin-PC left 4	0	6	0	0
hugin-RG right 1	0	0	0	0
hugin-RG right 2	0	0	0	0
hugin-RG left 1	0	0	0	0
hugin-RG left 2	0	0	0	0
hugin-VNC right 1	0	0	0	0
hugin-VNC right 2	0	0	0	0
hugin-VNC left 1	0	0	0	0
hugin-VNC left 2	0	0	0	0
hugin-PH right 1	0	0	0	0
hugin-PH right 2	0	0	0	0
hugin-PH left 1	0	0	0	0
hugin-PH left 2	0	0	0	0

24.

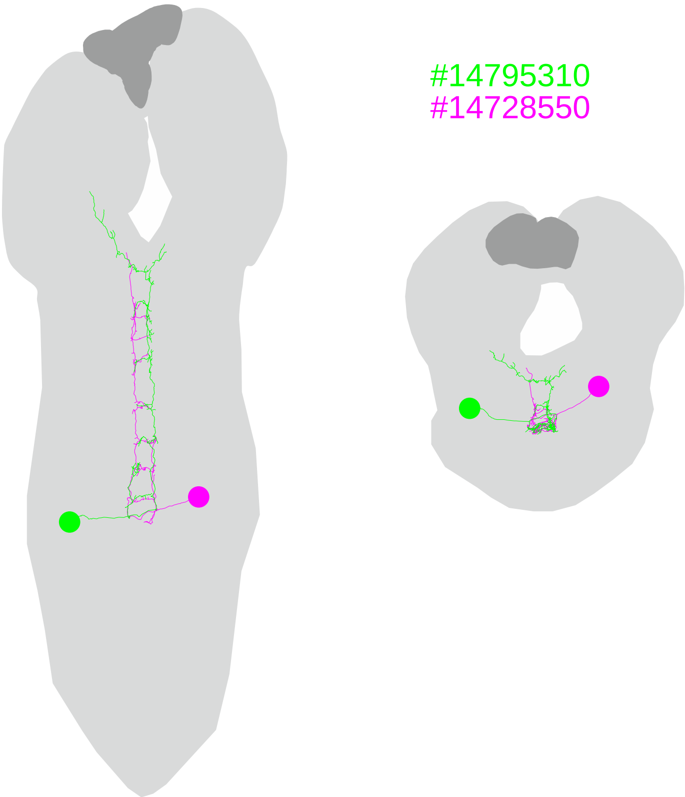

	synapses to		synapses from	
hugin-PC right 1	0	0	0	0
hugin-PC right 2	0	0	0	0
hugin-PC right 3	0	0	0	0
hugin-PC right 4	0	0	0	0
hugin-PC left 1	0	0	0	0
hugin-PC left 2	0	0	0	0
hugin-PC left 3	0	0	0	0
hugin-PC left 4	0	0	0	0
hugin-RG right 1	0	0	0	0
hugin-RG right 2	0	0	0	0
hugin-RG left 1	0	0	0	0
hugin-RG left 2	0	0	0	0
hugin-VNC right 1	0	0	0	0
hugin-VNC right 2	0	0	0	0
hugin-VNC left 1	0	0	0	0
hugin-VNC left 2	0	0	0	0
hugin-PH right 1	3	0	0	0
hugin-PH right 2	0	0	0	0
hugin-PH left 1	2	0	0	0
hugin-PH left 2	0	2	0	0

25.

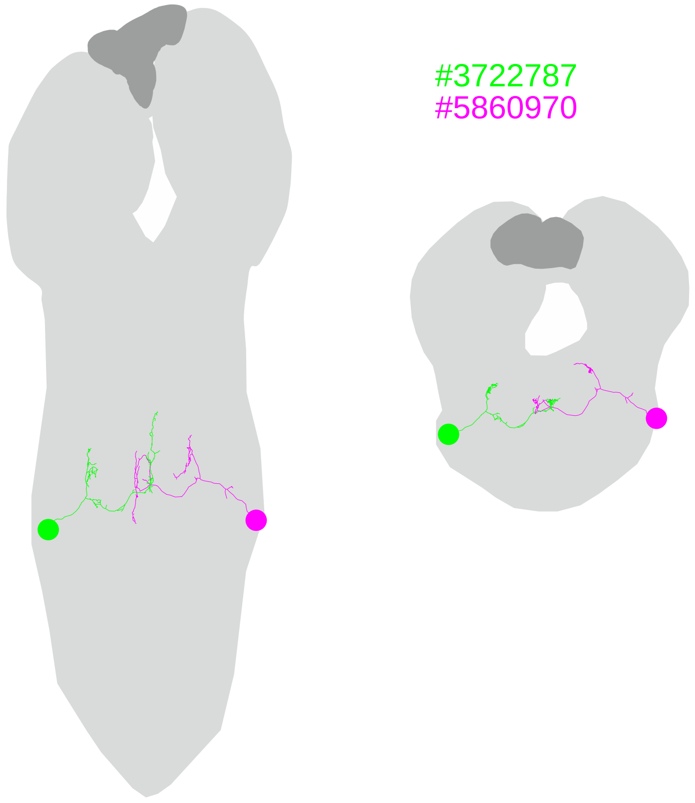

	synapses to		synapses from	
hugin-PC right 1	0	0	0	0
hugin-PC right 2	0	0	0	0
hugin-PC right 3	0	0	0	0
hugin-PC right 4	0	0	0	0
hugin-PC left 1	0	0	0	0
hugin-PC left 2	0	0	0	0
hugin-PC left 3	0	0	0	0
hugin-PC left 4	0	0	0	0
hugin-RG right 1	0	0	0	0
hugin-RG right 2	0	0	0	0
hugin-RG left 1	0	0	0	0
hugin-RG left 2	0	0	0	0
hugin-VNC right 1	0	0	5	0
hugin-VNC right 2	0	0	1	0
hugin-VNC left 1	0	0	0	6
hugin-VNC left 2	0	0	0	4
hugin-PH right 1	0	0	0	0
hugin-PH right 2	0	0	0	0
hugin-PH left 1	0	0	0	0
hugin-PH left 2	0	0	0	0

26.

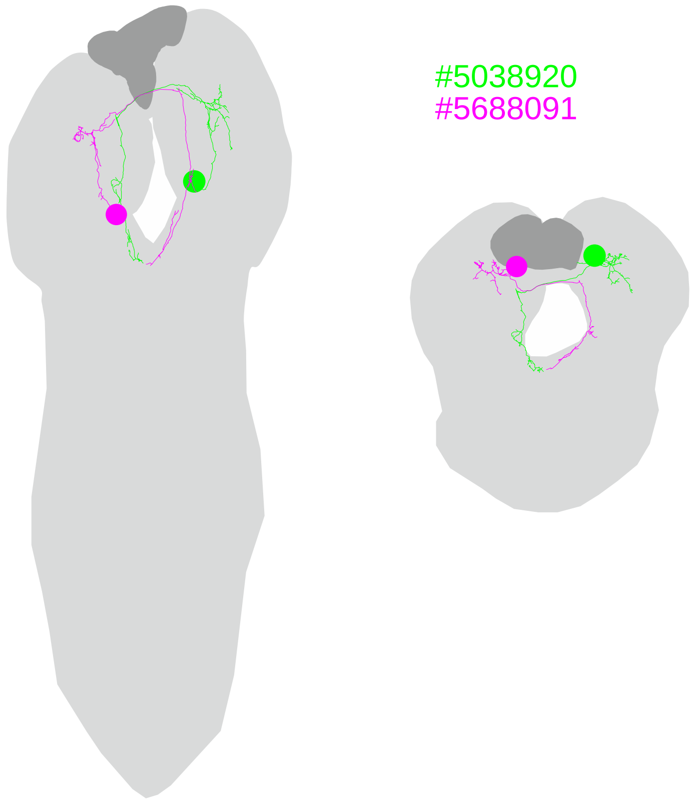

	synapses to		synapses from	
hugin-PC right 1	0	0	0	0
hugin-PC right 2	0	0	0	0
hugin-PC right 3	0	0	0	0
hugin-PC right 4	0	0	0	0
hugin-PC left 1	0	0	0	0
hugin-PC left 2	0	0	0	0
hugin-PC left 3	2	0	0	0
hugin-PC left 4	0	0	0	0
hugin-RG right 1	5	6	0	0
hugin-RG right 2	5	8	0	0
hugin-RG left 1	11	2	0	0
hugin-RG left 2	12	2	0	0
hugin-VNC right 1	0	0	0	0
hugin-VNC right 2	2	0	0	0
hugin-VNC left 1	0	0	0	0
hugin-VNC left 2	0	0	0	0
hugin-PH right 1	0	0	0	0
hugin-PH right 2	0	0	0	0
hugin-PH left 1	0	0	0	0
hugin-PH left 2	0	0	0	0

27.

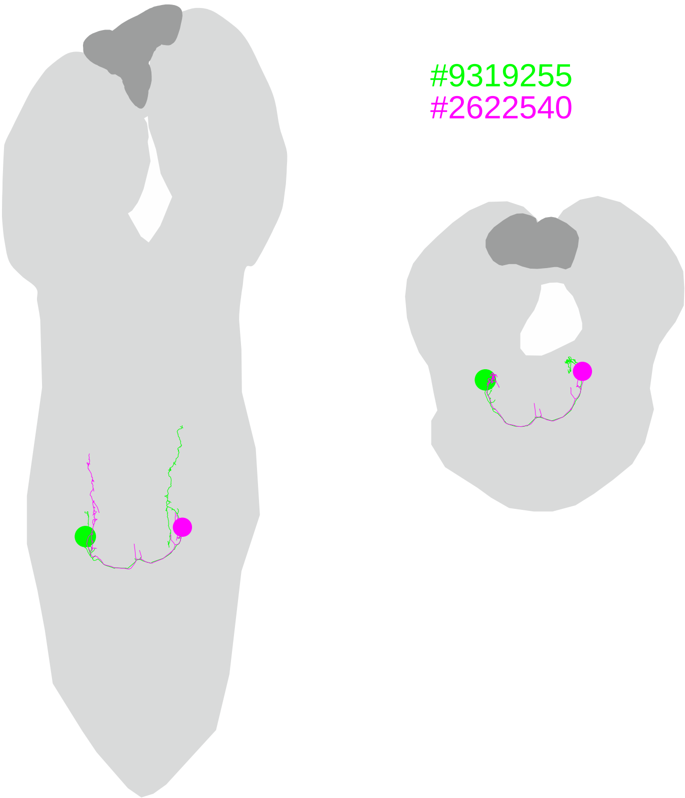

	synapses to		synapses from	
hugin-PC right 1	0	0	0	0
hugin-PC right 2	0	0	0	0
hugin-PC right 3	0	0	0	0
hugin-PC right 4	0	0	0	0
hugin-PC left 1	0	0	0	0
hugin-PC left 2	0	0	0	0
hugin-PC left 3	0	0	0	0
hugin-PC left 4	0	0	0	0
hugin-RG right 1	0	0	0	0
hugin-RG right 2	0	0	0	0
hugin-RG left 1	0	0	0	0
hugin-RG left 2	0	0	0	0
hugin-VNC right 1	0	0	7	0
hugin-VNC right 2	0	0	0	0
hugin-VNC left 1	0	0	0	2
hugin-VNC left 2	0	0	0	1
hugin-PH right 1	0	0	0	0
hugin-PH right 2	0	0	0	0
hugin-PH left 1	0	0	0	0
hugin-PH left 2	0	0	0	0

28.

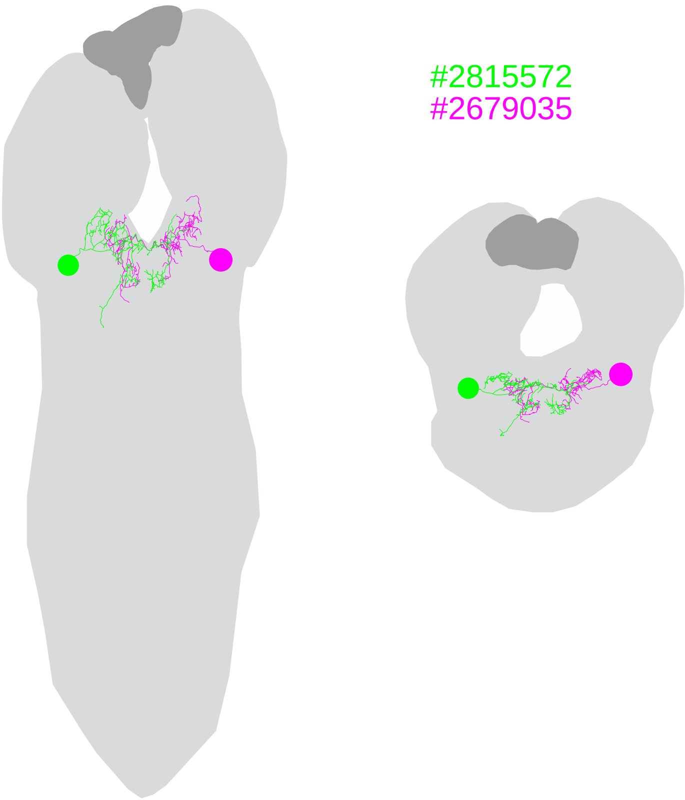

	synapses to		synapses from	
hugin-PC right 1	0	0	0	0
hugin-PC right 2	0	0	0	0
hugin-PC right 3	0	0	0	0
hugin-PC right 4	0	1	0	0
hugin-PC left 1	0	0	0	0
hugin-PC left 2	0	0	0	0
hugin-PC left 3	0	1	1	0
hugin-PC left 4	0	0	0	0
hugin-RG right 1	0	0	0	0
hugin-RG right 2	0	0	0	0
hugin-RG left 1	0	0	0	0
hugin-RG left 2	0	0	0	0
hugin-VNC right 1	0	0	0	0
hugin-VNC right 2	0	0	0	0
hugin-VNC left 1	0	0	0	0
hugin-VNC left 2	0	0	0	0
hugin-PH right 1	3	0	0	0
hugin-PH right 2	2	0	0	0
hugin-PH left 1	0	1	0	0
hugin-PH left 2	1	2	0	0

29.

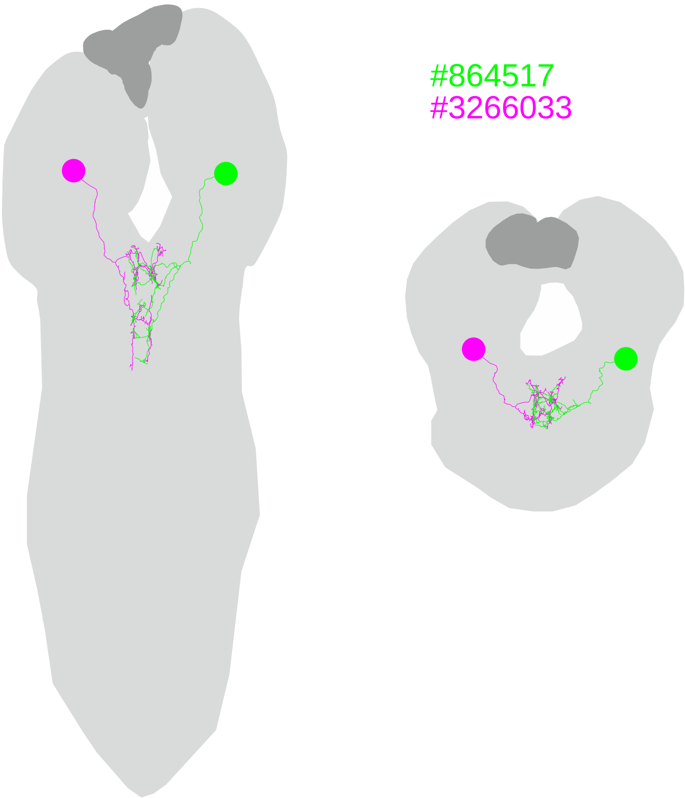

	synapses to		synapses from	
hugin-PC right 1	0	0	0	0
hugin-PC right 2	0	0	0	0
hugin-PC right 3	0	0	0	0
hugin-PC right 4	0	0	0	0
hugin-PC left 1	0	0	0	0
hugin-PC left 2	0	0	0	0
hugin-PC left 3	0	0	0	0
hugin-PC left 4	0	0	0	0
hugin-RG right 1	0	0	0	0
hugin-RG right 2	0	0	0	0
hugin-RG left 1	0	0	0	0
hugin-RG left 2	0	0	0	0
hugin-VNC right 1	0	0	0	0
hugin-VNC right 2	0	0	0	0
hugin-VNC left 1	0	0	0	0
hugin-VNC left 2	0	0	0	0
hugin-PH right 1	0	2	0	0
hugin-PH right 2	0	1	0	0
hugin-PH left 1	0	0	0	0
hugin-PH left 2	5	2	0	0

30.

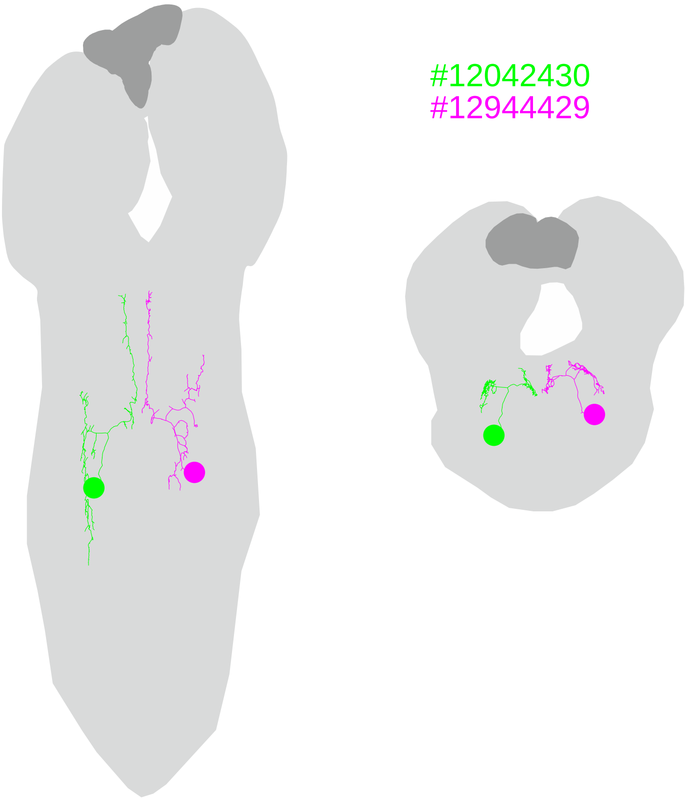

	synapses to		synapses from	
hugin-PC right 1	0	0	0	0
hugin-PC right 2	0	0	0	0
hugin-PC right 3	0	0	0	0
hugin-PC right 4	0	0	0	0
hugin-PC left 1	0	0	0	0
hugin-PC left 2	0	0	0	0
hugin-PC left 3	0	0	0	0
hugin-PC left 4	0	0	0	0
hugin-RG right 1	0	0	0	0
hugin-RG right 2	0	0	0	0
hugin-RG left 1	0	0	0	0
hugin-RG left 2	0	0	0	0
hugin-VNC right 1	0	0	7	0
hugin-VNC right 2	0	0	7	0
hugin-VNC left 1	0	0	0	3
hugin-VNC left 2	0	0	0	3
hugin-PH right 1	0	0	0	0
hugin-PH right 2	0	0	0	0
hugin-PH left 1	0	0	0	0
hugin-PH left 2	0	0	0	0

31.

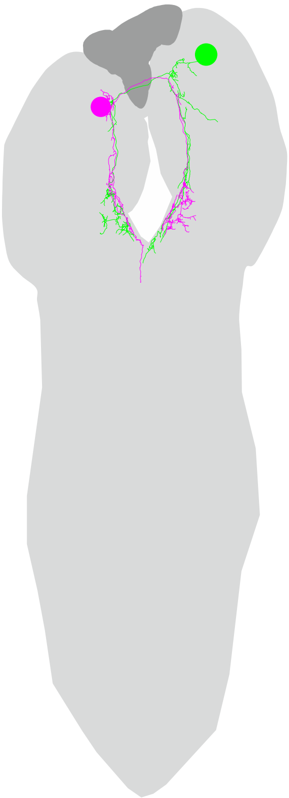

#3436420
#6445994

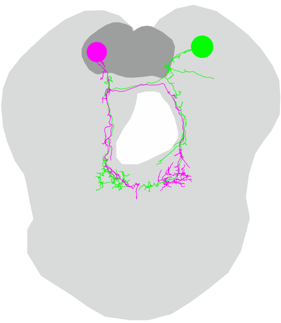

	synapses to		synapses from	
hugin-PC right 1	0	0	0	2
hugin-PC right 2	0	0	0	2
hugin-PC right 3	0	1	2	0
hugin-PC right 4	0	0	3	1
hugin-PC left 1	0	0	0	2
hugin-PC left 2	1	0	1	0
hugin-PC left 3	0	0	1	2
hugin-PC left 4	2	1	1	1
hugin-RG right 1	0	0	0	0
hugin-RG right 2	0	0	0	0
hugin-RG left 1	0	0	0	0
hugin-RG left 2	0	0	0	0
hugin-VNC right 1	0	0	0	0
hugin-VNC right 2	1	0	0	0
hugin-VNC left 1	1	0	0	0
hugin-VNC left 2	0	0	0	0
hugin-PH right 1	0	0	0	0
hugin-PH right 2	0	0	0	0
hugin-PH left 1	0	0	0	0
hugin-PH left 2	0	0	0	0

32.

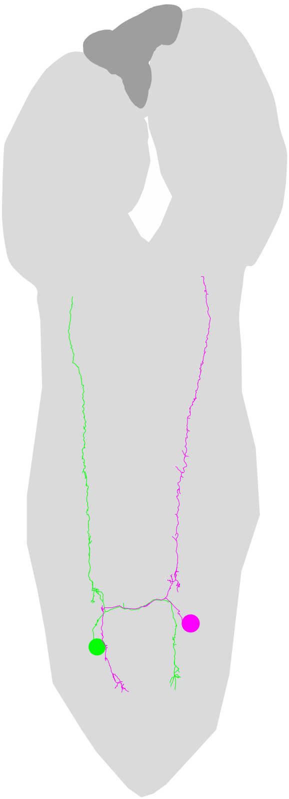

#8010889
#2622502

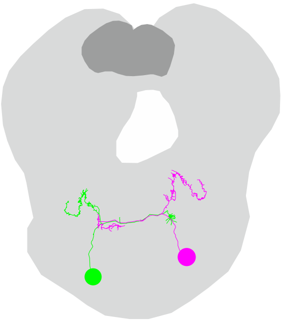

	synapses to		synapses from	
hugin-PC right 1	0	0	0	0
hugin-PC right 2	0	0	0	0
hugin-PC right 3	0	0	0	0
hugin-PC right 4	0	0	0	0
hugin-PC left 1	0	0	0	0
hugin-PC left 2	0	0	0	0
hugin-PC left 3	0	0	0	0
hugin-PC left 4	0	0	0	0
hugin-RG right 1	0	0	0	0
hugin-RG right 2	0	0	0	0
hugin-RG left 1	0	0	0	0
hugin-RG left 2	0	0	0	0
hugin-VNC right 1	8	0	2	0
hugin-VNC right 2	8	0	1	0
hugin-VNC left 1	0	8	0	4
hugin-VNC left 2	0	13	0	3
hugin-PH right 1	0	0	0	0
hugin-PH right 2	0	0	0	0
hugin-PH left 1	0	0	0	0
hugin-PH left 2	0	0	0	0

33.

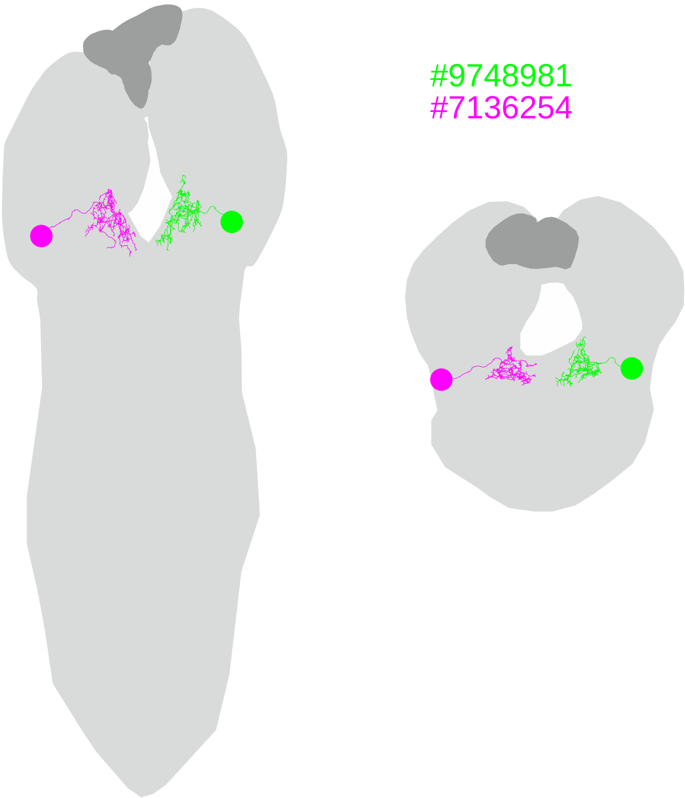

	synapses to		synapses from	
hugin-PC right 1	1	0	0	0
hugin-PC right 2	0	0	0	0
hugin-PC right 3	2	0	3	0
hugin-PC right 4	0	0	2	0
hugin-PC left 1	0	0	0	2
hugin-PC left 2	0	0	0	1
hugin-PC left 3	0	0	0	0
hugin-PC left 4	0	0	0	1
hugin-RG right 1	0	0	0	0
hugin-RG right 2	0	0	0	0
hugin-RG left 1	0	0	0	0
hugin-RG left 2	0	0	0	0
hugin-VNC right 1	0	0	0	0
hugin-VNC right 2	0	1	0	0
hugin-VNC left 1	0	0	0	0
hugin-VNC left 2	0	0	0	0
hugin-PH right 1	0	0	0	0
hugin-PH right 2	0	0	0	0
hugin-PH left 1	0	0	0	0
hugin-PH left 2	0	0	0	0

34.

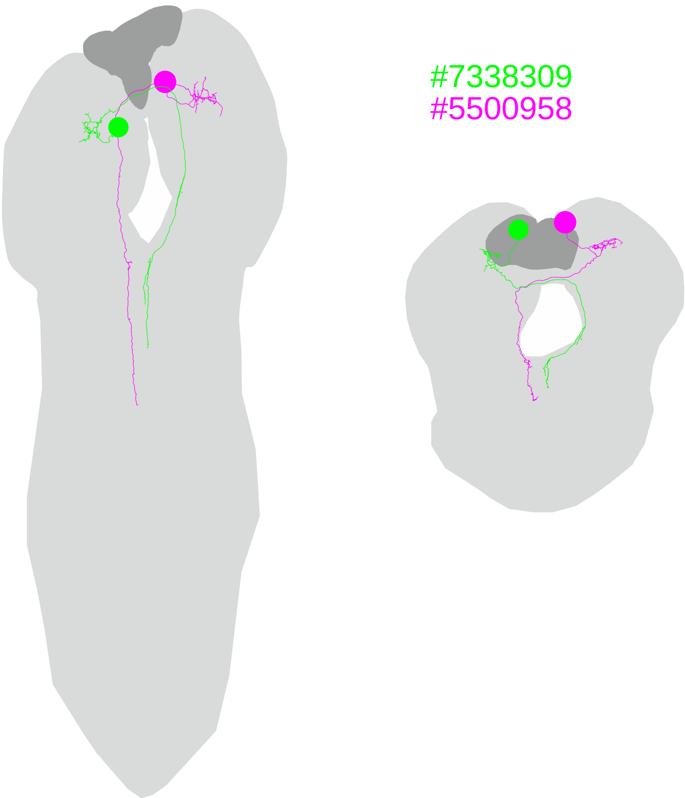

	synapses to		synapses from	
hugin-PC right 1	0	0	0	0
hugin-PC right 2	0	0	0	0
hugin-PC right 3	0	0	0	0
hugin-PC right 4	0	0	0	0
hugin-PC left 1	0	0	0	0
hugin-PC left 2	0	0	0	0
hugin-PC left 3	0	0	0	0
hugin-PC left 4	0	0	0	0
hugin-RG right 1	0	0	0	0
hugin-RG right 2	0	0	0	0
hugin-RG left 1	0	0	0	0
hugin-RG left 2	0	0	0	0
hugin-VNC right 1	0	0	0	0
hugin-VNC right 2	0	0	0	0
hugin-VNC left 1	3	0	0	0
hugin-VNC left 2	0	0	0	0
hugin-PH right 1	0	0	0	0
hugin-PH right 2	0	0	0	0
hugin-PH left 1	0	0	0	0
hugin-PH left 2	0	0	0	0

35.

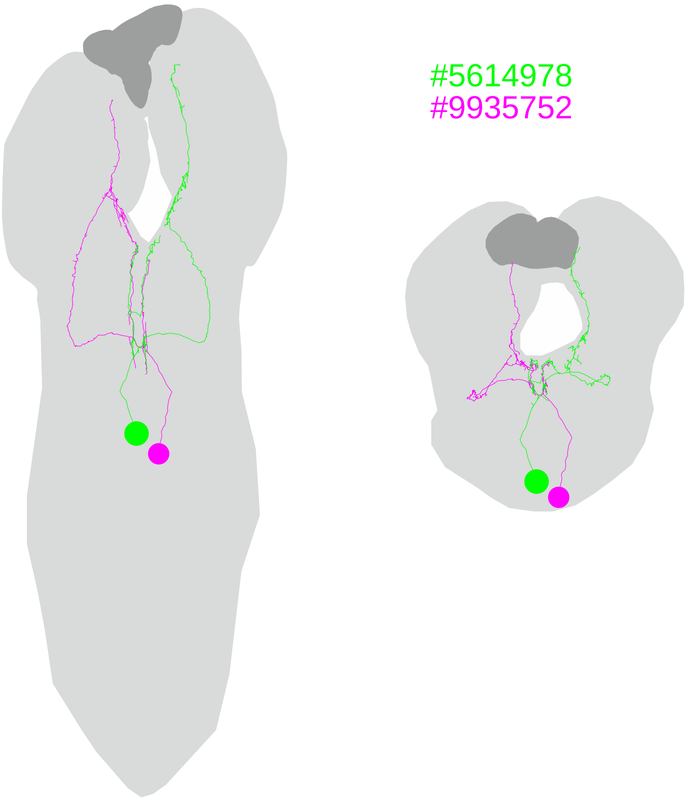

	synapses to		synapses from	
hugin-PC right 1	0	0	0	0
hugin-PC right 2	7	0	1	0
hugin-PC right 3	2	0	2	0
hugin-PC right 4	2	0	0	0
hugin-PC left 1	0	0	0	0
hugin-PC left 2	0	0	0	0
hugin-PC left 3	0	1	0	0
hugin-PC left 4	0	4	0	0
hugin-RG right 1	1	0	0	0
hugin-RG right 2	3	0	0	0
hugin-RG left 1	0	0	0	0
hugin-RG left 2	0	1	0	0
hugin-VNC right 1	0	0	0	1
hugin-VNC right 2	0	2	0	0
hugin-VNC left 1	0	0	0	0
hugin-VNC left 2	0	0	0	0
hugin-PH right 1	0	0	0	0
hugin-PH right 2	0	0	0	0
hugin-PH left 1	0	0	0	0
hugin-PH left 2	0	0	0	0

36.

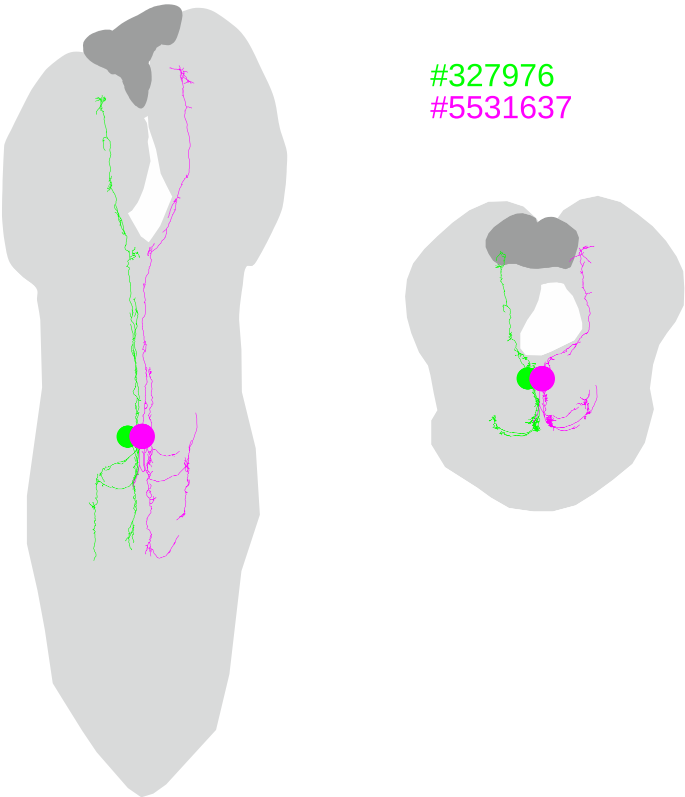

	synapses to		synapses from	
hugin-PC right 1	0	0	0	0
hugin-PC right 2	0	0	0	0
hugin-PC right 3	0	0	0	0
hugin-PC right 4	0	0	0	0
hugin-PC left 1	0	0	0	0
hugin-PC left 2	1	0	0	0
hugin-PC left 3	4	0	0	0
hugin-PC left 4	0	0	0	0
hugin-RG right 1	1	2	0	0
hugin-RG right 2	1	3	0	0
hugin-RG left 1	8	2	0	0
hugin-RG left 2	0	0	0	0
hugin-VNC right 1	2	5	0	0
hugin-VNC right 2	10	0	0	0
hugin-VNC left 1	5	4	0	0
hugin-VNC left 2	0	1	0	0
hugin-PH right 1	0	0	0	0
hugin-PH right 2	0	0	0	0
hugin-PH left 1	0	0	0	0
hugin-PH left 2	0	0	0	0

37.

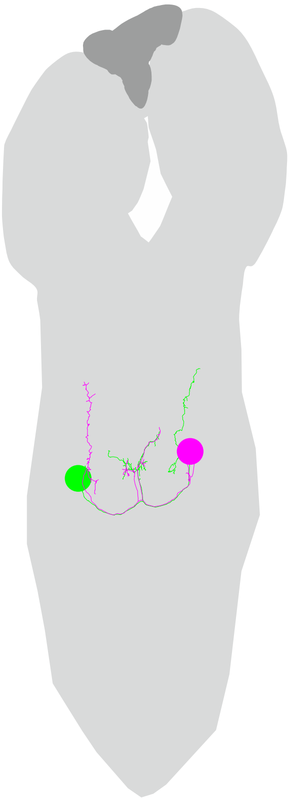

#8748462
#4284599

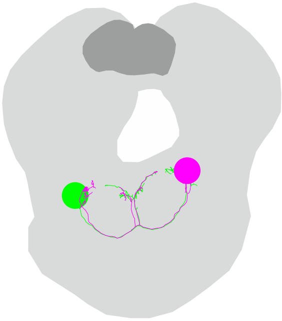

	synapses to		synapses from	
hugin-PC right 1	0	0	0	0
hugin-PC right 2	0	0	0	0
hugin-PC right 3	0	0	0	0
hugin-PC right 4	0	0	0	0
hugin-PC left 1	0	0	0	0
hugin-PC left 2	0	0	0	0
hugin-PC left 3	0	0	0	0
hugin-PC left 4	0	0	0	0
hugin-RG right 1	0	0	0	0
hugin-RG right 2	0	0	0	0
hugin-RG left 1	0	0	0	0
hugin-RG left 2	0	0	0	0
hugin-VNC right 1	0	0	3	0
hugin-VNC right 2	0	0	1	0
hugin-VNC left 1	0	0	0	3
hugin-VNC left 2	0	0	0	1
hugin-PH right 1	0	0	0	0
hugin-PH right 2	0	0	0	0
hugin-PH left 1	0	0	0	0
hugin-PH left 2	0	0	0	0

38.

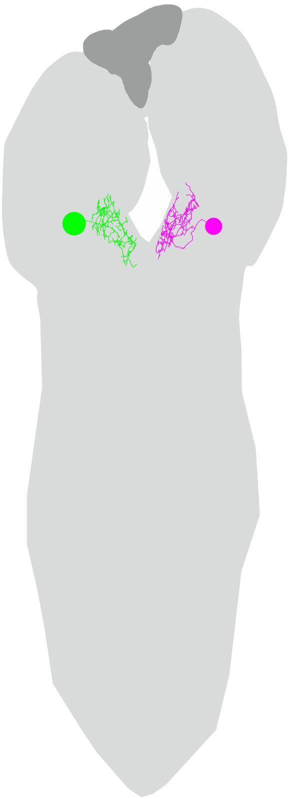

#4284106
#9758503

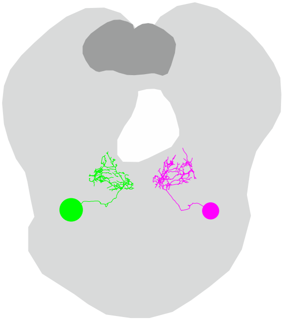

	synapses to		synapses from	
hugin-PC right 1	0	0	0	2
hugin-PC right 2	0	0	0	0
hugin-PC right 3	0	2	0	0
hugin-PC right 4	0	1	0	0
hugin-PC left 1	1	0	1	0
hugin-PC left 2	0	0	3	0
hugin-PC left 3	0	0	1	0
hugin-PC left 4	3	0	0	0
hugin-RG right 1	0	0	0	0
hugin-RG right 2	0	0	0	0
hugin-RG left 1	0	0	0	0
hugin-RG left 2	0	0	0	0
hugin-VNC right 1	0	0	0	0
hugin-VNC right 2	0	0	0	0
hugin-VNC left 1	0	0	0	0
hugin-VNC left 2	0	0	0	0
hugin-PH right 1	0	0	0	0
hugin-PH right 2	0	0	0	0
hugin-PH left 1	0	0	0	0
hugin-PH left 2	0	0	0	0

39.

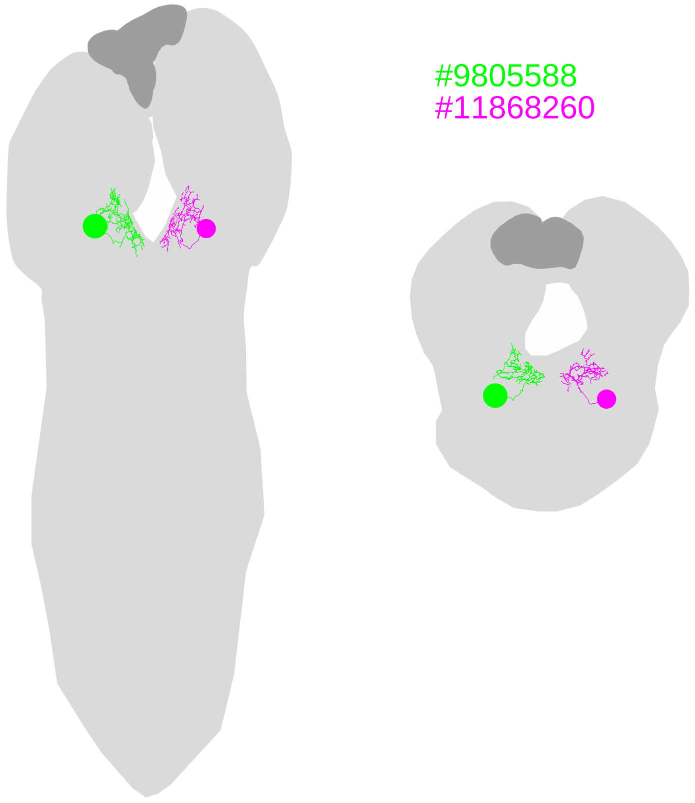

	synapses to		synapses from	
hugin-PC right 1	0	0	0	0
hugin-PC right 2	0	2	0	0
hugin-PC right 3	0	1	0	0
hugin-PC right 4	0	0	0	0
hugin-PC left 1	5	0	2	0
hugin-PC left 2	1	0	1	0
hugin-PC left 3	0	0	1	0
hugin-PC left 4	1	0	1	0
hugin-RG right 1	0	0	0	0
hugin-RG right 2	0	0	0	0
hugin-RG left 1	0	0	0	0
hugin-RG left 2	0	0	0	0
hugin-VNC right 1	0	0	0	0
hugin-VNC right 2	0	0	0	0
hugin-VNC left 1	0	0	0	0
hugin-VNC left 2	0	0	0	0
hugin-PH right 1	0	0	0	0
hugin-PH right 2	0	0	0	0
hugin-PH left 1	0	0	0	0
hugin-PH left 2	0	0	0	0

40.

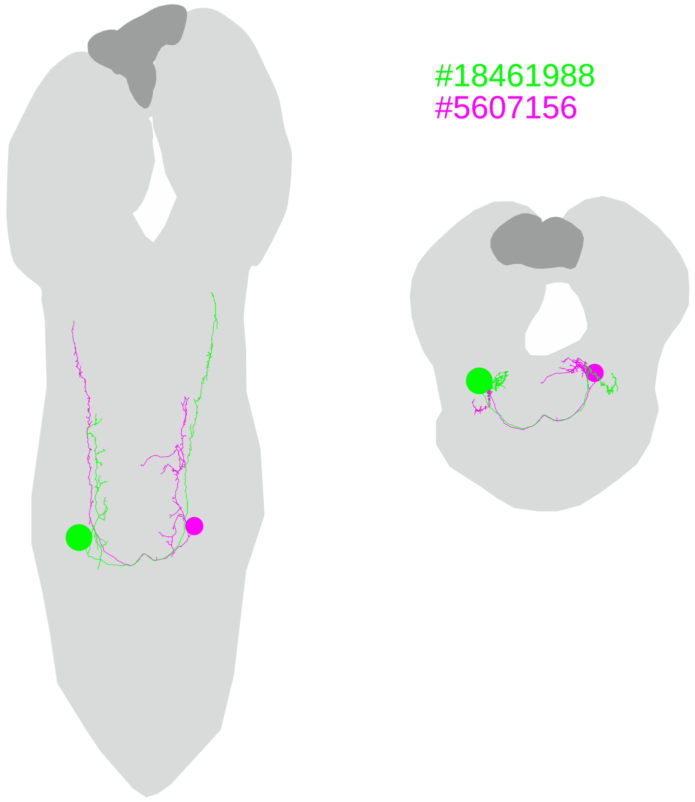

	synapses to		synapses from	
hugin-PC right 1	0	0	0	0
hugin-PC right 2	0	0	0	0
hugin-PC right 3	0	0	0	0
hugin-PC right 4	0	0	0	0
hugin-PC left 1	0	0	0	0
hugin-PC left 2	0	0	0	0
hugin-PC left 3	0	0	0	0
hugin-PC left 4	0	0	0	0
hugin-RG right 1	0	0	0	0
hugin-RG right 2	0	0	0	0
hugin-RG left 1	0	0	0	0
hugin-RG left 2	0	0	0	0
hugin-VNC right 1	0	3	1	1
hugin-VNC right 2	0	2	1	5
hugin-VNC left 1	1	0	4	0
hugin-VNC left 2	5	0	2	1
hugin-PH right 1	0	0	0	0
hugin-PH right 2	0	0	0	0
hugin-PH left 1	0	0	0	0
hugin-PH left 2	0	0	0	0

41.

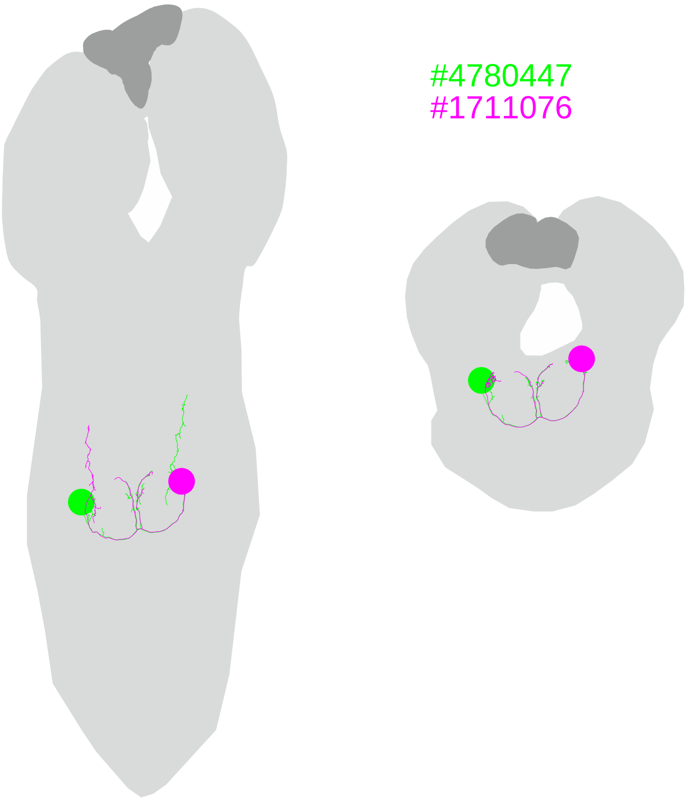

	synapses to		synapses from	
hugin-PC right 1	0	0	0	0
hugin-PC right 2	0	0	0	0
hugin-PC right 3	0	0	0	0
hugin-PC right 4	0	0	0	0
hugin-PC left 1	0	0	0	0
hugin-PC left 2	0	0	0	0
hugin-PC left 3	0	0	0	0
hugin-PC left 4	0	0	0	0
hugin-RG right 1	0	0	0	0
hugin-RG right 2	0	0	0	0
hugin-RG left 1	0	0	0	0
hugin-RG left 2	0	0	0	0
hugin-VNC right 1	0	0	3	0
hugin-VNC right 2	0	0	0	0
hugin-VNC left 1	0	0	0	2
hugin-VNC left 2	0	0	0	1
hugin-PH right 1	0	0	0	0
hugin-PH right 2	0	0	0	0
hugin-PH left 1	0	0	0	0
hugin-PH left 2	0	0	0	0

42.

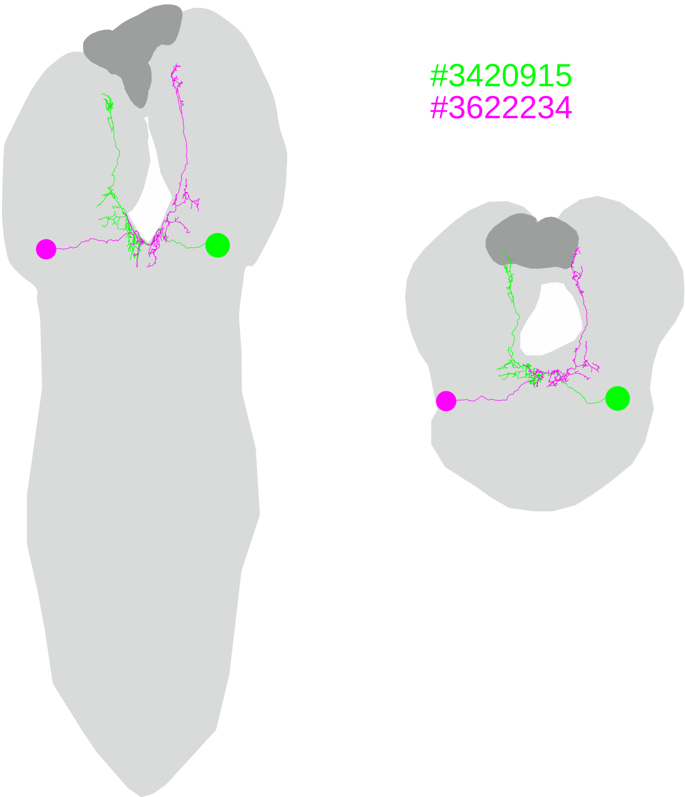

	synapses to		synapses from	
hugin-PC right 1	0	0	0	0
hugin-PC right 2	0	0	0	1
hugin-PC right 3	0	1	0	1
hugin-PC right 4	0	0	0	2
hugin-PC left 1	2	0	0	0
hugin-PC left 2	0	0	2	0
hugin-PC left 3	2	0	4	0
hugin-PC left 4	0	0	1	1
hugin-RG right 1	0	0	0	0
hugin-RG right 2	0	0	0	0
hugin-RG left 1	0	0	0	0
hugin-RG left 2	0	0	0	0
hugin-VNC right 1	0	2	0	0
hugin-VNC right 2	1	0	0	0
hugin-VNC left 1	4	0	1	0
hugin-VNC left 2	0	2	0	0
hugin-PH right 1	0	0	0	0
hugin-PH right 2	0	0	0	0
hugin-PH left 1	0	0	0	0
hugin-PH left 2	0	0	0	0

43.

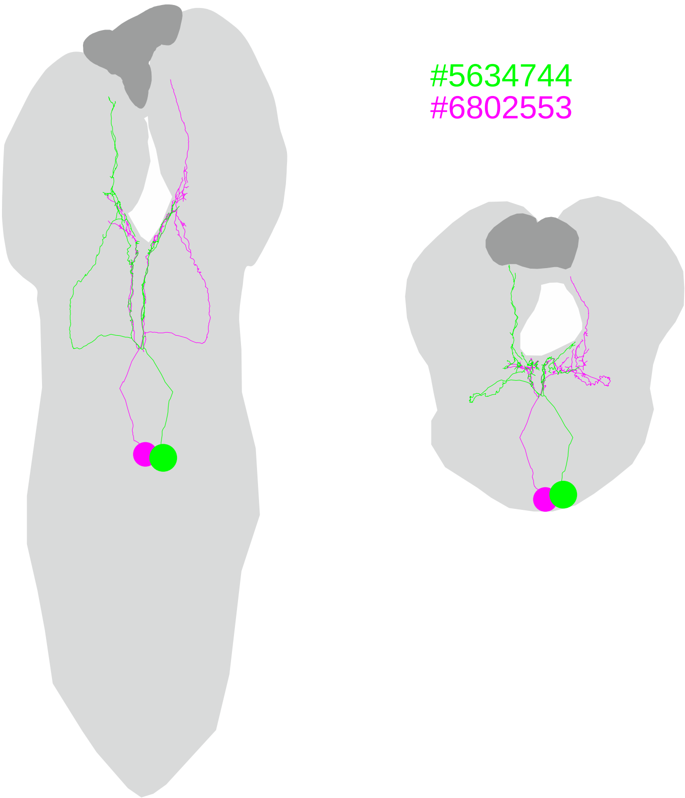

	synapses to		synapses from	
hugin-PC right 1	0	10	0	2
hugin-PC right 2	0	11	0	4
hugin-PC right 3	0	7	0	1
hugin-PC right 4	0	2	0	0
hugin-PC left 1	6	0	2	1
hugin-PC left 2	5	0	1	0
hugin-PC left 3	8	0	2	0
hugin-PC left 4	5	0	0	0
hugin-RG right 1	0	0	0	0
hugin-RG right 2	0	0	0	0
hugin-RG left 1	0	0	0	0
hugin-RG left 2	0	0	0	0
hugin-VNC right 1	0	0	0	0
hugin-VNC right 2	0	0	0	0
hugin-VNC left 1	0	0	0	0
hugin-VNC left 2	0	0	0	0
hugin-PH right 1	0	0	0	0
hugin-PH right 2	0	0	0	0
hugin-PH left 1	0	0	0	0
hugin-PH left 2	0	0	0	0

K Unpaired medial interneurons

1.

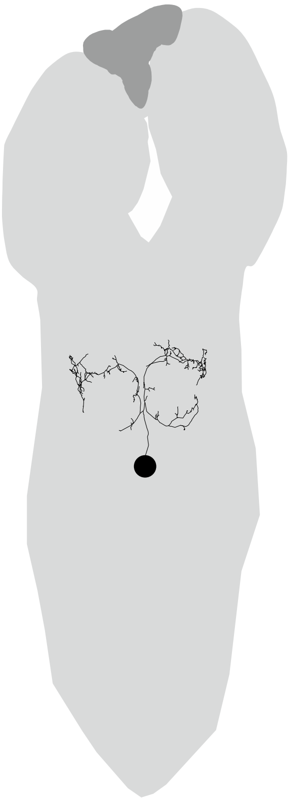

#7165165

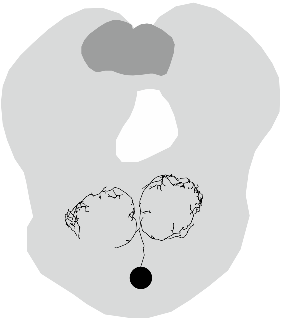

	synapses to	synapses from
hugin-PC right 1	0	0
hugin-PC right 2	0	0
hugin-PC right 3	0	0
hugin-PC right 4	0	0
hugin-PC left 1	0	0
hugin-PC left 2	0	0
hugin-PC left 3	0	0
hugin-PC left 4	0	0
hugin-RG right 1	0	0
hugin-RG right 2	0	0
hugin-RG left 1	0	0
hugin-RG left 2	0	0
hugin-VNC right 1	0	5
hugin-VNC right 2	0	4
hugin-VNC left 1	0	2
hugin-VNC left 2	0	3
hugin-PH right 1	0	0
hugin-PH right 2	0	0
hugin-PH left 1	0	0
hugin-PH left 2	0	0

2.

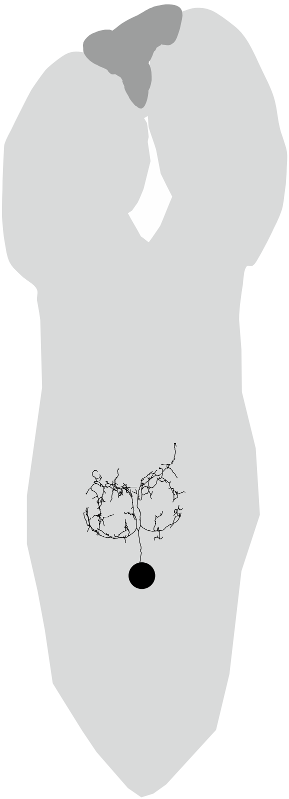

#14091666

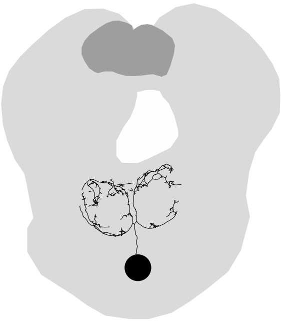

	synapses to	synapses from
hugin-PC right 1	0	0
hugin-PC right 2	0	0
hugin-PC right 3	0	0
hugin-PC right 4	0	0
hugin-PC left 1	0	0
hugin-PC left 2	0	0
hugin-PC left 3	0	0
hugin-PC left 4	0	0
hugin-RG right 1	0	0
hugin-RG right 2	0	0
hugin-RG left 1	0	0
hugin-RG left 2	0	0
hugin-VNC right 1	0	0
hugin-VNC right 2	0	3
hugin-VNC left 1	0	0
hugin-VNC left 2	0	0
hugin-PH right 1	0	0
hugin-PH right 2	0	0
hugin-PH left 1	0	0
hugin-PH left 2	0	0
